# Microbial enzyme-responsive inulin nanoparticles for colon-targeted oral delivery of 5-ASA toward improved therapy of inflammatory bowel disease

**DOI:** 10.7150/thno.127560

**Published:** 2026-08-24

**Authors:** Haena Park, Panmo Son, Chiwoo Oh, Hyeryeon Oh, Jee-Eun Hwang, Seo Young Kim, Jeongcheol Lee, Jae Kyoo Lee, Won Il Choi, Hyung-Jun Im

**Affiliations:** 1 Research Institute for Convergence Science, Seoul National University, Seoul 08826, Republic of Korea.; 2 Bio-Convergence Materials R&D Division, Korea Institute of Ceramic Engineering and Technology, 202, Osongsaengmyeong 1-ro, Osong-eup, Heungdeok-gu, Cheongju, Chungbuk 28160, Republic of Korea.; 3 Department of Applied Bioengineering, Graduate School of Convergence Science and Technology, Seoul National University, Seoul 08826, Republic of Korea.; 4 Precision Vaccine Program, Department of Pediatrics, Boston Children’s Hospital, Boston, MA, USA.; 5 Harvard Medical School, Boston, MA, USA.; 6 Department of Molecular Medicine and Biopharmaceutical Sciences, Graduate School of Convergence Science and Technology, Seoul National University, Seoul 03080, Republic of Korea.; 7 Cancer Research Institute, Seoul National University, Seoul 03080, Republic of Korea.

**Keywords:** inflammatory bowel disease (IBD), inulin nanoparticles (INP), 5-aminosalicylic acid (5-ASA), colon-targeted delivery, enzyme-responsive release

## Abstract

Colon-targeted delivery of 5-aminosalicylic acid (5-ASA) offers an effective strategy for treating inflammatory bowel disease (IBD) while minimizing systemic side effects. However, conventional oral 5-ASA formulations suffer from premature drug release and poor colon specificity. To overcome these limitations, we developed a biodegradable, prebiotic, and immunomodulatory inulin nanoparticle (INP) platform enabling microbial enzyme-responsive and site-specific release of 5-ASA in the colon.

**Methods:**

INP was fabricated via a mild nanoprecipitation method and loaded with 5-ASA (5-ASA@INP). Physicochemical characteristics, drug-loading performance, and enzyme-responsive sequential release profiles under simulated gastrointestinal fluids were evaluated. Antioxidant and anti-inflammatory effects were assessed *in vitro*. Biodistribution of DiR-labeled INPs was analyzed to demonstrate colon-targeting efficacy. Therapeutic efficacy, inflammatory cytokine expression, Treg responses in the colon and gut-associated lymphoid tissues, and microbiota-related effects, including short-chain fatty acid (SCFA) production, were evaluated in a colitis mouse model.

**Results:**

5-ASA@INP showed 97.02% drug-loading efficiency. The cumulative release of 5-ASA was 7.6% in simulated gastric fluid, 18.7% in simulated intestinal fluid, and 99.2% in simulated colonic fluid. In contrast, only 35.6% of 5-ASA was released under enzyme-free simulated colonic conditions. Compared with free 5-ASA, 5-ASA@INP showed enhanced antioxidant and anti-inflammatory effects *in vitro*. *In vivo*, INPs preferentially accumulated in the colon with minimal off-target distribution. In colitic mice, 5-ASA@INP significantly attenuated body weight loss (*p* = 0.0067) and colon shortening (*p* = 0.0002). Histological analysis showed that the colonic tissue damage was minimized and the inflammation scores were reduced (*p* < 0.001). Treatment increased the number of FOXP3+ cells in the colonic tissue and suppressed the expression of IL-17A, TNF-α, and IL-1β. The gut microbial profile was restored toward that of the normal group and fecal SCFA levels were increased.

**Conclusions:**

This study demonstrates that the INP platform enables microbial enzyme-responsive, colon-targeted delivery of 5-ASA while complementarily harnessing the intrinsic prebiotic and immunomodulatory properties of inulin. This dual-function nanoplatform provides a promising strategy for efficient oral treatment of IBD.

## Introduction

Inflammatory bowel disease (IBD), specifically including both ulcerative colitis (UC) and Crohn’s disease, is an inflammatory gastrointestinal (GI) tract disorder lasting indefinitely 1,2. Approximately 6.8 million people were afflicted with IBD as of 2017, and it was found to have a prevalence rate of approximately 84.3 individuals per 100,000 3. The mucosa of the colon is commonly inflamed in UC and the inflammation starts at the rectum and continues proximally up the colon. Symptoms often include abdominal pain, weight loss, diarrhea, fever, bloody stool. Although the exact mechanism of UC remains unclear, its pathogenesis involves interactions between environmental factors, genetic predisposition, alterations in the gut microbiome, and a dysregulated immune response 4,5.

Current therapeutic strategies for IBD consist of 5-aminosalicylates (5-ASA) and corticosteroids, and novel immunomodulatory agents that include tumor necrosis factor-alpha (TNF-α) inhibitors, antiinterleukin-23 (IL-23) and IL-12 biologics, and Janus kinase (JAK) inhibitors 6. Among the anti-inflammatory agents, 5-ASA is the mainstay of treatment for UC. One of its key mechanisms of action is the suppression of inflammatory cytokine production, such as interleukin-1β (IL-1β) and TNF-α, including inhibition of nuclear factor-κB (NF-κB) transcription factor activation. Furthermore, 5-ASA exhibits antioxidant properties by scavenging reactive oxygen species (ROS), such as peroxyl radicals, and modulating various inflammatory mediators within the inflamed colonic environment 7.

Although oral administration of 5-ASA is effective in managing UC 8-10, its therapeutic efficacy is limited by physicochemical instability and premature absorption in the upper GI tract. The chemical degradation of 5-ASA can be influenced by environmental factors such as temperature, pH, oxygen, and light 11,12. Furthermore, exposure to the upper GI tract prior to the colon can lead to premature absorption and systemic distribution, resulting in side effects such as headache, dyspepsia, nausea or allergic reactions. This may also result in lower concentrations of the drug in the colon reducing its therapeutic efficacy against IBD 13,14.

Colon-targeted drug delivery systems have been proposed as an effective strategy to overcome the physicochemical instability and premature upper gastrointestinal absorption of 5-ASA. Ahmad *et al.* developed 5-ASA-loaded gelatin nanoparticles coated with Eudragit S100, an enteric polymer. This enteric-coated nanoparticle formulation reduced colonic inflammation and lowered the expression levels of IL-1β and TNF-α 15. Park *et al.* developed prodrug nanoassemblies comprising 5-ASA and cathepsin B-cleavable peptides via an amide bond reaction in which carbonyldiimidazole was added to dimethylformamide containing 5-ASA and the peptide. These nanoassemblies demonstrated cellular uptake and *in vitro* anti-inflammatory effects. They were also retained in the GI tract, enhancing therapeutic efficacy by reduction in the number of pro-inflammatory cytokines; specifically, TNF-α, IL-1β, IL-6, and Interferon-γ (IFN-γ) 16. However, there remain challenges for the use of these carriers such as the complexity of the fabrication processes, insufficient efficacy, and passive delivery without additional benefits.

Inulin, a naturally occurring fermentable polysaccharide found in more than 36,000 plant species, presents a promising alternative. Recognized as a prebiotic fiber by the US Food and Drug Administration in 2018, inulin exerts multiple beneficial effects on colon health, including anti-inflammatory activity, mucosal layer restoration, gut microbiome regulation, and immune modulation 17,18. Notably, inulin resists digestion in the upper GI tract and is selectively fermented by colonic bacteria that produce inulinase 19. This fermentation process yields short-chain fatty acids (SCFAs)―such as butyric acid and propionic acid―which serve as critical mediators in the microbiota-SCFA-immune axis. By promoting the induction of regulatory T cells (Tregs) and suppressing proinflammatory responses, these SCFAs play a pivotal role in modulating the intestinal immune homeostasis 20. Owing to these favorable properties, inulin is a promising candidate for colon-targeted drug delivery, offering both protective and therapeutic functionalities.

**Figure 1:**
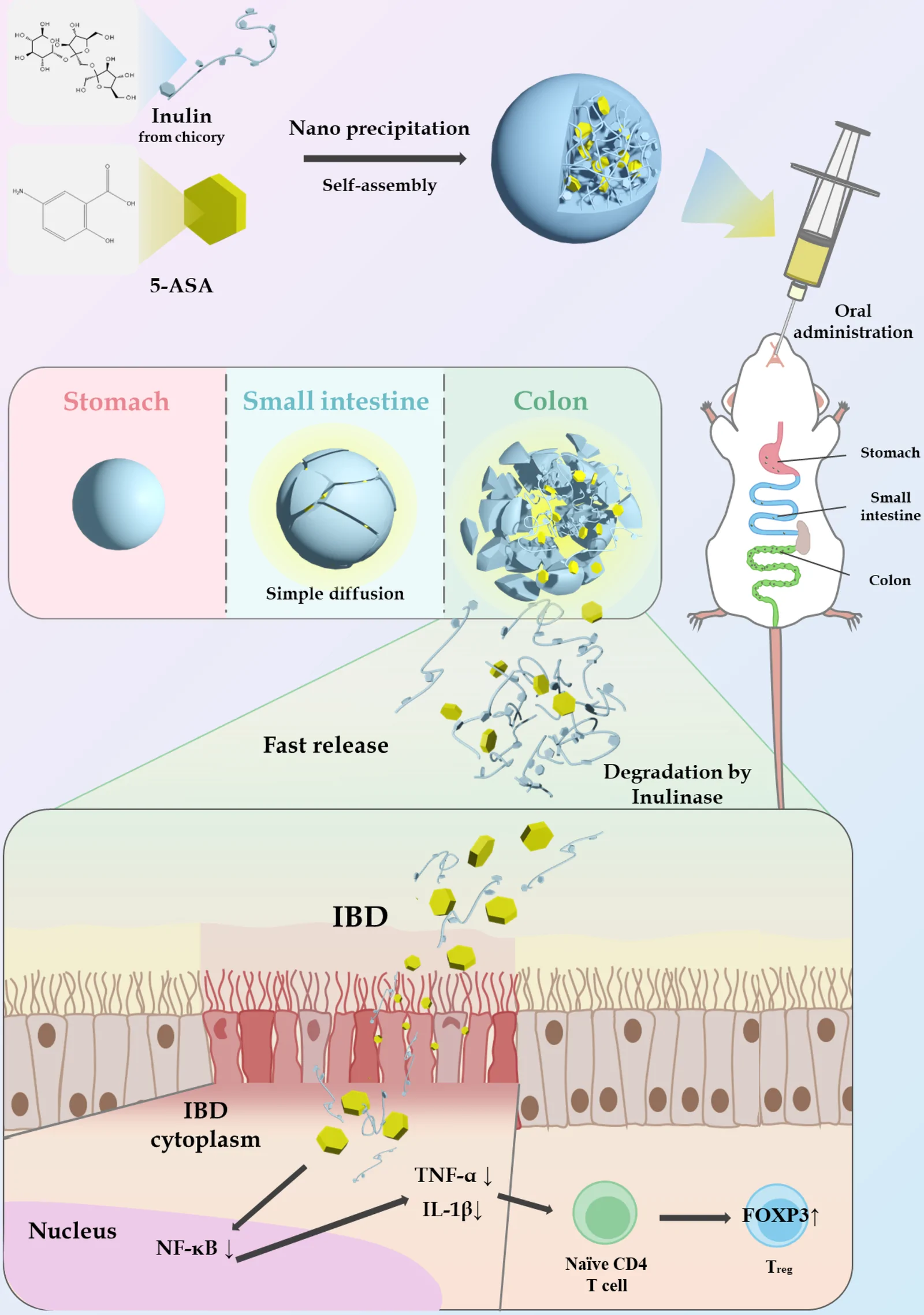
** Schematic illustration of 5-ASA@INP preparation and its therapeutic mechanism involving colon-targeted delivery and immune modulation**.

In this study, we developed colon-targeted inulin nanoparticles (INP) to enhance the oral delivery and therapeutic efficacy of 5-ASA for UC, as illustrated in **Figure 1**. INP was prepared via a simple nanoprecipitation method utilizing the intermolecular interactions of the fructose-rich structure of inulin, without any chemical conjugation or toxic crosslinking. The physicochemical properties, colloidal stability, and loading efficiency of 5-ASA-loaded INP (5-ASA@INP) were evaluated, and their microbial enzyme-responsive release examined under simulated colonic conditions. Furthermore, the therapeutic efficacy and microbiota-related effects of 5-ASA@INP were assessed in a dextran sulfate sodium (DSS)-induced UC mouse model. Collectively, this study demonstrates the potential of enzyme-responsive, inulin-based nanoparticles as a prebiotic oral delivery platform for colon-targeted therapy and explores their possible anti-inflammatory and mucosal protective effects for improved treatment of UC.

## Materials and Methods

### Materials

Inulin from chicory (Cat. No. I2255, Lot No. SLCG2442, ~6 kDa), dimethyl sulfoxide (DMSO), pepsin (from porcine gastric mucosa), pancreatin (from porcine pancreas), inulinase (from *Aspergillus niger*), and Griess reagent were purchased from Sigma-Aldrich (St. Louis, MO, USA). Hyclone™ deionized water (DIW) and phosphate-buffered saline (PBS) were obtained from GE Healthcare Life Sciences (Little Chalfont, USA). 5-ASA was purchased from Tokyo Chemical Industry (Tokyo, Japan). 1,1'-Dioctadecyl-3,3,3',3'-tetramethylindotricarbocyanine iodide (DiR), IL-1β antibody, TNF-α antibody, and tyramide conjugates were obtained from Thermo Fisher Scientific (Waltham, MA, USA). Acetonitrile (HPLC grade) was purchased from Honeywell (Charlotte, NC, USA).

NIH-3T3 fibroblasts were obtained from the American Type Culture Collection (Rockville, MD, USA). Dulbecco’s modified Eagle’s medium (DMEM), fetal bovine serum (FBS), penicillin-streptomycin (PS), CellROX™ Green (C10444), 3-(4,5-dimethylthiazol-2-yl)-2,5-diphenyltetrazolium bromide (MTT), and trypsin were purchased from Thermo Fisher Scientific (Waltham, MA, USA). The Cell Counting Kit-8 (CCK-8) was obtained from Dojindo Laboratories (Kumamoto, Japan).

### Preparation and characterization of INP and 5-ASA@INP

INP was fabricated by a simple nanoprecipitation process, leveraging the hydrophobic interactions within the fructose-rich structure of inulin. To optimize the formulation conditions, different inulin concentrations (10, 20, 50, 100, and 200 mg) were screened, and the resulting nanoparticles were assessed for particle size, size distribution, surface charge, and post-lyophilization redispersion properties. Inulin was dissolved in 1 mL of DMSO and shaken on a rotary shaker for 2 h (13 rpm, 25 ºC). To initiate nanoprecipitation, 1 mL of the inulin solution was added dropwise into 5 mL of DIW using a syringe pump (KD LEGATO 100 infuse single; KD Scientific; MA, USA) equipped with a 30-gauge needle at a constant flow rate of 0.1 mL/min, under continuous magnetic stirring at 530 rpm (25 ºC). After 1 h of stirring, the resulting nanoparticle dispersion was lyophilized using a freeze-dryer (FDCF-12015; Operon; Gimpo, Korea) for 144 h to remove residual DMSO. Following freeze-drying, INP was resuspended in DIW to a final concentration of 1 mg/mL, and the INP suspension was centrifuged for purification at 2,000 rpm and 25 ºC using an Amicon Ultra-15 spin filter (100 kDa MWCO; Merck Millipore, Billerica, MA, USA). The residual DMSO content in INP was quantified via an HPLC system equipped with a C18 column (5 μm; SunFire® C18 column, Waters Corp.) and UV detector (Waters 2487). A mixture of 70% acetonitrile and 30% water (v/v) was used as the mobile phase, with a flow rate of 0.1 mL/min for 10 min, and DMSO was detected at 210 nm. In addition, 5-ASA@INP was prepared by dissolving 5-ASA (0.5, 1, 2.5, and 5 mg) in DMSO (1 mL), together with 50 mg of inulin, followed by nanoprecipitation under the same conditions used for INPs.

To characterize INPs and 5-ASA@INPs, the freeze-dried INP and 5-ASA@INP were resuspended in DIW to a final concentration of 1 mg/mL. The physicochemical properties of the nanoparticles were analyzed at 25 ºC via dynamic light scattering (DLS; ELSZ-2000, Otsuka Electronics, Tokyo, Japan), including hydrodynamic diameter (size), particle size distribution, polydispersity index (PDI), and zeta potential. For morphological analysis, the INP and 5-ASA@INP suspensions were dropped onto 200-mesh carbon film grids, air-dried for 48 h, and observed by transmission electron microscopy (TEM; JEM-2100Plus HR; JEOL, Tokyo, Japan). In addition, FT-IR and UV-Vis spectroscopy were performed to further characterize 5-ASA@INP. For FT-IR analysis, freeze-dried inulin, INP, 5-ASA and 5-ASA@INP samples were analyzed over the range of 4000-400 cm⁻¹ using an FT-IR spectrometer (Nicolet™ summit X FT-IR, Thermo Fisher Scientific, Waltham, MA, USA). For UV-Vis analysis, the absorbance spectra of INP and 5-ASA@INP were measured using a UV-Vis spectrometer (Mega900; Scinco, Seoul, Republic of Korea). The loading efficiency and loading content of 5-ASA were determined by measuring the amount of non-loaded 5-ASA. To separate the unloaded 5-ASA, the 5-ASA@INP resuspension was subjected to centrifugal filtration at 1,800 rpm and 25 ℃ for 60 min using Amicon Ultra-15 centrifugal filters with MWCO of 100 kDa. The absorbance of 5-ASA in the filtrate was measured at 330 nm using a microplate reader (SpectraMax iD3; Molecular Devices; San Jose, CA, USA) 21. The loading content (L.C.) and loading efficiency (L.E.) values of the loaded 5-ASA in INP were calculated as follows:

Loading content (L.C., %) = (Weight of feeding 5-ASA – Weight of unloaded 5-ASA) / Weight of INP × 100

Loading efficiency (L.E., %) = (Weight of feeding 5-ASA – Weight of unloaded 5-ASA) / Weight of feeding 5-ASA × 100

### Stability and drug release of 5-ASA@INP

To evaluate the stability of INP after lyophilization, freeze-dried INP was redispersed in 5 mL of DIW and PBS. The dispersions were analyzed via DLS, as described in the previous section. To assess the long-term stability of INP, 5 mg of nanoparticles were redispersed in 5 mL of PBS (pH 7.4) and monitored via DLS at 37 ºC under gentle stirring. Physicochemical properties including particle size and PDI were monitored over three weeks via DLS. To further assess the stability of 5-ASA@INP under different storage conditions, the nanoparticles were redispersed and stored at 4 ºC, 25 ºC, 37 ºC, and 37 ºC in the presence of 10% FBS. Particle size, PDI and drug-loading retention were evaluated after storage under each condition. For all stability analyses, the 0-week time point was defined after 1 h of storage under each condition.

The drug release profile of 5-ASA from 5-ASA@INP was investigated under simulated gastrointestinal conditions, following previously reported methods 22-24. The release media were prepared according to the guidelines of the US Pharmacopeia for simulated gastric, intestinal, and colonic fluids (SGF, SIF, and SCF). SGF was prepared by dissolving 600 mg of sodium chloride in 300 mL of DIW. Pepsin (960 mg) was added to the sodium chloride solution and the pH was adjusted to 1.2 with hydrochloric acid. To prepare SIF, 2.04 g of potassium dihydrogen phosphate was dissolved in 300 mL of DIW, followed by addition of 3 g of pancreatin and adjustment of pH to 6.8 with sodium hydroxide. SCF was prepared by dissolving inulinase in PBS (pH 7.4) to obtain a final enzyme activity of 2.5 U/mL (0.1 mg/mL), and enzyme-free PBS (pH 7.4) was used as a control condition. The 5-ASA@INP solution (1 mL) was placed into a Float-A-Lyzer G2 Dialysis device (molecular weight cut off = 100 kDa; Spectra/Por Dialysis Membrane, Repligen, Waltham, MA, USA) and incubated sequentially in 10 mL of SGF for 2 h, followed by transfer to 10 mL of SIF for 4 h, and then to 10 mL of SCF for 18 h, under constant stirring at 37 ºC and 100 rpm. At predetermined time points, the medium was collected and replaced with an equal volume of fresh medium. To assess total drug recovery, the solution remaining inside the dialysis device was collected and analyzed at the end point of the release study. The released 5-ASA was quantified by measuring the absorbance (302 nm for SGF; 330 nm for SIF and SCF) using a microplate reader (SpectraMax iD3; Molecular Devices, San Jose, CA, USA) 23.

### Caco-2 cell culture

Caco-2 cells (RRID:CVCL_0025; human colon adenocarcinoma epithelial cells, male origin) were obtained from the Korean Cell Line Bank (KCLB No. 30037.1; Seoul, Korea) at passage 30. For consistency, only cells within passage 35 to 45 were used. The Caco-2 cells were cultured at 37 ºC in DMEM supplemented with 10% (v/v) FBS and a 1% (v/v) antibiotic-antimycotic mixture in a humidified atmosphere of 5% CO₂. The cells were passaged with 0.25% trypsin-EDTA when they reached 80% confluence. Mycoplasma contamination was not detected in the Caco-2 cells used.

### *In vitro* cytotoxicity

Cell viability was assessed using a modified MTT assay 25. Caco-2 cells were seeded at a density of 1 × 10⁵ cells per well of a 96-well plate and incubated for 48 h. Various concentrations of 5-ASA and INP were administered to the cells. After 24 h of incubation, the culture medium was replaced with fresh medium supplemented with MTT at a final concentration of 0.5 mg/mL. The MTT reaction was allowed to proceed for 3 h and was terminated by aspirating the medium. After formation of formazan crystals, they were dissolved in 150 μL of DMSO. The resulting formazan solution was then analyzed by measuring absorbance at 570 nm using a microplate reader. For each treatment group, cell viability was expressed as a percentage of viable cells relative to that of untreated controls.

### Measurement of intracellular reactive oxygen species levels

Intracellular reactive oxygen species (ROS) levels were measured using CellROX™ Green dye as a fluorescent ROS probe. Caco-2 cells were seeded in 24-well plates at a density of 1 × 10⁵ cells/cm². After the cells had reached 80% confluence, they were treated for 1 h with increasing concentrations of 5-ASA, 5-ASA@INP, or INP. A fresh H₂O₂ solution was prepared and added to the culture medium to achieve a final concentration of 9.6 mM, followed by incubation for 2 h. After incubation, CellROX Green was added at a final concentration of 5 μM, and the cells were incubated for an additional 30 min in the dark. After staining, the cells were rinsed with Dulbecco’s Phosphate-Buffered Saline (DPBS) and detached using trypsin. ROS levels were quantified via flow cytometry (Guava® easyCyte™5, Cytek®; Fremont, CA, USA) by measuring the mean fluorescence intensity (MFI). The representative flow cytometry histograms for each sample at various concentrations are provided in Supplementary Information (**Figure S12**). ROS levels are expressed as the relative fluorescence intensity (RFI) normalized to the positive control group. The positive control group consisted of cells treated with H₂O₂ alone, whereas the negative control group consisted of untreated cells.

### Measurement of nitric oxide production

Caco-2 cells were cultured for 21 days to allow full differentiation. To assess nitric oxide (NO) production, the cells were stimulated with lipopolysaccharide (LPS; 100 μg/mL) for 24 h. The total nitrate/nitrite levels were determined using the Total Nitric Oxide Assay Kit (Biomax), following the manufacturer’s instructions. Briefly, the culture medium was enzymatically processed with nitrate reductase, and the resulting nitrite concentrations were quantified colorimetrically using the Griess reagent.

### Cellular uptake

Cellular uptake was evaluated using confocal laser scanning microscopy (CLSM; AR HD25; Nikon, Tokyo, Japan). Cells were incubated with the samples under serum-free conditions for 3 h. After incubation, the cells were washed with PBS and fixed with 70% ethanol. CLSM images were acquired at 350 nm to visualize the intrinsic fluorescence of 5-ASA. The fluorescence intensity was analyzed using ImageJ software and expressed as mean fluorescence intensity (MFI, a.u.) for relative comparison among groups.

### Animals and colitis model

Female C57BL/6 mice (8 weeks old) were obtained from Orient Bio Inc. (Gapyeong Center, Gyeonggi-do, Korea). All animal experiments were reviewed and approved by the Institutional Animal Care and Use Committee (IACUC) of Seoul National University (SNU-240501-6-3). The mice were maintained under specific pathogen-free (SPF) housing conditions. Colitis was induced by administering drinking water containing 3% (w/v) DSS (36–50 kDa) for 7 days, followed by 2 days of normal drinking water.

### Biodistribution study of orally administered DiR-loaded INP

For biodistribution analysis, 8-week-old female C57BL/6 mice were randomly assigned to two groups at each time point: DiR solution (n = 4 biologically independent mice per time point) and DiR@INP (n = 6 biologically independent mice per time point). DiR-loaded nanoparticles (DiR@INP) were prepared using the same method described previously for nanoparticle formulation. The mice were orally administered either a DiR solution or DiR@INP at an equivalent DiR dose of 2 mg/kg (200 μL per mouse) via oral gavage. At designated time points (2, 4, 8, and 12 h post-administration), the mice were anesthetized with isoflurane and euthanized. The entire GI tract was excised, and the major digestive organs, including the stomach, small intestine, and colon, were collected. Fluorescent images of the harvested organs were acquired using an *in vivo* imaging system (IVIS; PerkinElmer, Waltham, MA, USA). Fluorescence emission was detected at an excitation wavelength of 754 nm and an emission filter of 778 nm. Living Image software was used to measure the fluorescence intensity within manually selected regions of interest (ROI). The fluorescence intensity within the colon was expressed as a percentage of the total fluorescence signal across the GI tract (Colon Target %). To minimize interference from tissue autofluorescence, fluorescence signals were analyzed under identical imaging settings using blank tissues as background references, and the data were interpreted as relative fluorescence intensity rather than absolute biodistribution.

### *In vivo* therapeutic effect of 5-ASA@INP

A DSS-induced colitis model was established using 8-week-old female C57BL/6 mice. Two independent experiments were conducted under the same experimental conditions. In the confirmatory experiment, mice (n = 5 per group) were randomly assigned to the following groups: normal, saline, 5-ASA, INP, and 5-ASA@INP. Data from the two independent cohorts were combined for the analysis of therapeutic efficacy. The group-specific sample sizes used for the combined analysis were as follows: normal (n = 8), saline (n = 11), 5-ASA (n = 9), INP (n = 8), and 5-ASA@INP (n = 10).

To induce colitis, 3% DSS was provided in the drinking water for 6 days to all groups except the normal control. The test formulations were orally administered every 2 days. Body weight changes (%) were recorded daily to monitor disease progression. Upon completion of the experiment, the mice were euthanized in a CO₂ chamber, and the colons were harvested to measure their lengths and assess inflammation-induced shortening. The distal 3 cm portion of each colon was collected for weight measurement, reflecting granular immune cell infiltration, and for histological analysis via hematoxylin and eosin (H&E) staining. Histopathological changes were evaluated in the H&E-stained sections by a blinded observer, and inflammation severity was scored on a scale from 0 to 11 according to established criteria, incorporating criteria for inflammation severity (1–4), extent (1–3), epithelial hyperplasia (0 or 1), and ulceration (0 or 3). The oral dose of INP (2 g/kg) was selected based on previous literature demonstrating the high tolerability of inulin in murine models 26-28. To evaluate the systemic toxicity of this dose, serum was collected from mice after sacrifice, and biochemical markers including alanine aminotransferase (ALT), aspartate aminotransferase (AST), blood urea nitrogen (BUN), and creatinine (Crea) were measured.

### Immunofluorescence

Mouse colons were fixed in 4% paraformaldehyde (PFA), embedded in paraffin, and sectioned at a thickness of 4 μm. Deparaffinization and rehydration were performed by sequential incubation in xylene followed by ethanol at decreasing concentrations. For antigen retrieval, the sections were heated in 10 mM sodium citrate buffer (pH 6.0) at 100 ºC for 20 min. The sections were then washed with PBS and permeabilized in 0.5% Triton X-100 for 5 min.

Mouse Peyer’s patches (PPs) and mesenteric lymph nodes (MLNs) were embedded in Optimal Cutting Temperature (OCT) compound, cryosectioned at a thickness of 6 μm, and fixed with pre-cooled acetone at -20 ºC for 10 min. For immunofluorescence staining, all tissue sections were blocked with anti-mouse CD16/CD32 (eBioscience, 14-0161-85) to prevent nonspecific Fc-mediated binding.

The primary antibodies used were as follows: anti-CD3 (Invitrogen, 11-0032-82), anti-FOXP3 (Invitrogen, lot 2349824), anti-IL-17A (Affinity, DF6127), anti-TNF-α (Invitrogen, lot ZK4531382A), and anti-IL-1β (Invitrogen, lot ZJ4505521), anti-occludin (Invitrogen, A32790TR), anti-ZO-1 (Invitrogen, 41-9776-82). For TNF-α and IL-1β detection, tyramide signal amplification (TSA) was performed using Alexa Fluor™ 555 Tyramide (Invitrogen, lot 2881766). Fluorescence images were acquired using a confocal laser scanning microscope (CLSM; STELLARIS 5, Leica Microsystems, USA) and the fluorescence intensity was quantified using ImageJ software.

### Gut microbiota analysis

Fecal samples were collected from each mouse at the end of the therapeutic experiment and immediately stored at -80 ºC until analysis. Total microbial DNA was extracted from the samples and used for 16S rRNA gene sequencing. The bacterial 16S rRNA gene was amplified by PCR using barcoded primers targeting the V3―V4 hypervariable regions. The amplified PCR products were purified, and libraries were prepared using the Ion 520^TM^ & Ion 530^TM^ ExT Kit (Thermo Fisher Scientific, USA). Sequencing was performed on an Ion GeneStudioTM S5 system. Alpha diversity was assessed using Shannon and Simpson diversity indices. Beta diversity was evaluated based on Bray-Curtis distance matrices, and principal coordinate analysis (PCoA) was performed to visualize differences in microbial community composition among the experimental groups. Statistical differences in microbial community structure were further assessed using permutational multivariate analysis of variance (PERMANOVA).

### Short-chain fatty acid (SCFA) determination

The extraction procedure for short-chain fatty acids (SCFAs) from fecal samples was slightly modified from Zhang et al. 29. In brief, 50 mg of fecal samples were homogenized in 1.5 mL of 1 M HCl solution and centrifuged at 9,710 × g for 15 min. The supernatant was mixed with an equal volume of ethyl acetate, followed by centrifugation. The organic phase was collected and used for GC-MS analysis. SCFA concentrations were determined using an Orbitrap Exploris GC mass spectrometer (Thermo Fisher Scientific, Waltham, MA, USA) equipped with a TraceGOLD TG-WaxMS column (30 m × 0.25 mm i.d., 1.0 μm film thickness; Thermo Fisher Scientific). Helium was used as the carrier gas at a constant flow rate of 1.0 mL/min, and samples were injected in split mode with a split flow of 30 mL/min. The GC oven was programmed from 40 ºC (held for 0.5 min) to 60 ºC at 40 ºC/min (held for 2.0 min), then to 120 ºC at 4 ºC /min (held for 0.5 min), and finally to 230 ºC at 20 ºC /min (held for 10.0 min). The mass spectrometer was operated using electron ionization, and the ion source temperature was set to 250 ºC. The major fragment ions of the SCFA standards are shown in Supplementary Information (**Figure S25**). Based on these spectra, m/z 73.02840 and 60.02052 were selected for the quantitative analysis of propionic acid and butyric acid, respectively. To validate the extraction method, the recovery efficiency was evaluated by spiking standard solutions into fecal samples prior to extraction. The recovery rates of propionic acid and butyric acid were 88.16 ± 10.34% and 91.42 ± 2.65%, respectively.

### Statistical analysis

All data are expressed as mean ± standard deviation (SD). For comparisons involving more than two groups, one-way or two-way analysis of variance (ANOVA) followed by Tukey’s post-hoc test was employed. For comparison between two groups, Student’s T-test was employed. All statistical analyses were conducted using IBM SPSS Statistics software, version 29.0.1.0. Statistical significance was defined as *ns* (not significant, *p* > 0.05), *p < 0.05, **p < 0.01, and ***p < 0.001.

### Use of generative AI in the writing process

ChatGPT (OpenAI; GPT-4o and GPT-5 series, web interface version) was used for language and grammar editing throughout the manuscript during the writing and revision stages between March 2025 and July 2026, with default setting. Only author-written manuscript text was provided as input. The tool was not used for study design, data collection, data analysis, figure or image generation, literature searching or reference selection, and no AI-generated scientific content, interpretation, or conclusion is included in this manuscript. All AI-assisted output was reviewed and verified by Hyung-Jun Im, and text was revised or discarded where the tool altered the inaccurate or biased text and therefore verified all content against the primary data and the cited literature. The authors declare no financial or other relationship with any AI vendor. No AI is listed as an author or meets authorship criteria, and the authors take full responsibility for the accuracy and integrity of the manuscript.

## Results

### Physicochemical characteristics of 5-ASA@INP

**Figure 2:**
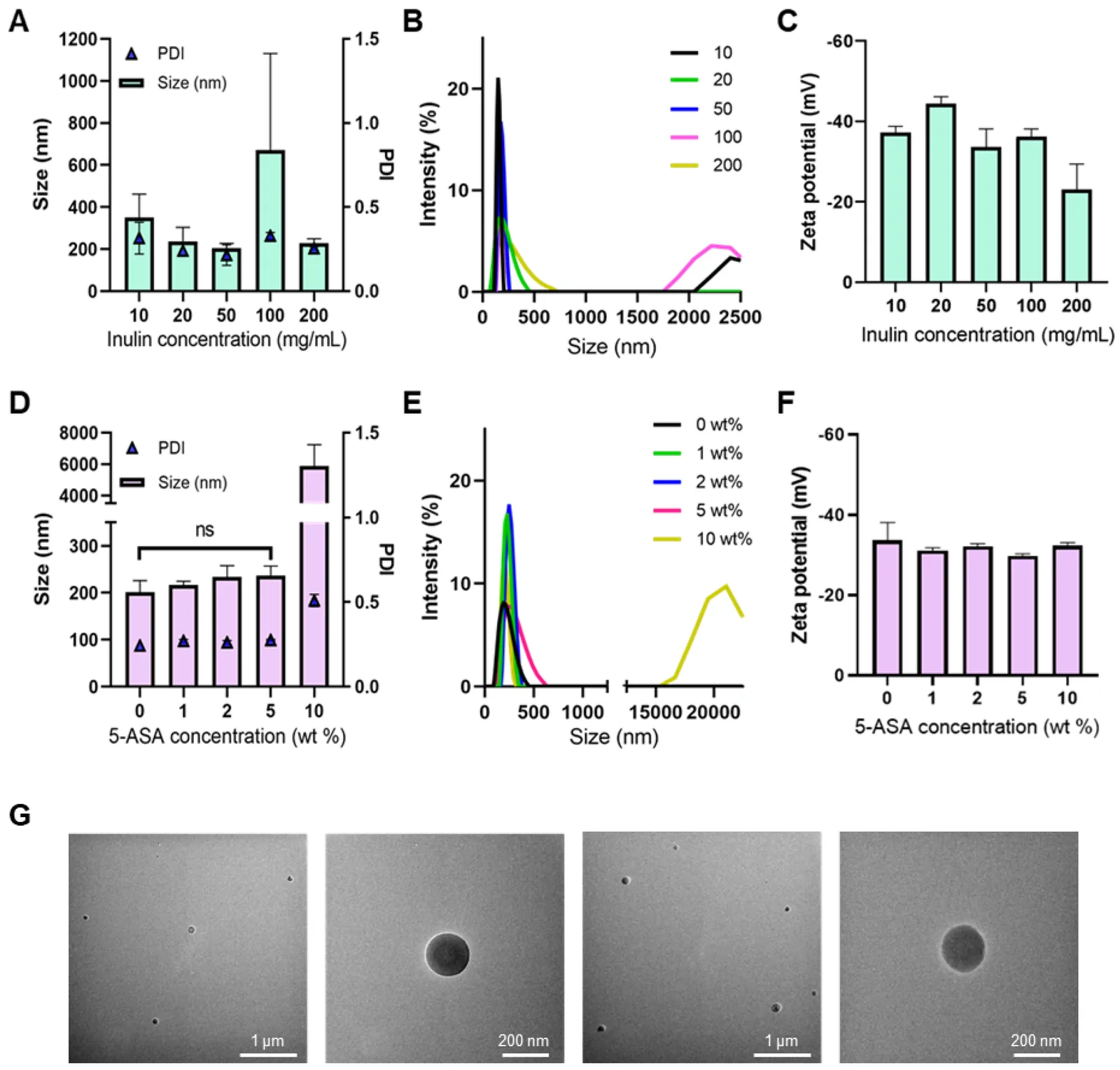
** Physicochemical characterization of INP and 5-ASA@INP.** (A) Size and polydispersity index (PDI), (B) size distribution, and (C) zeta potential of INPs by concentration of inulin. (D) Size and PDI, (E) size distribution, and (F) zeta potential of 5-ASA@INPs with increasing 5-ASA. (G) TEM images of INP (left two images) and 5-ASA@INP (right two images) at low and high magnification. Scale bars = 1 μm for low-magnification images and 200 nm for high magnification images. Data are presented as mean ± standard deviation (SD, n = 3). Statistical significance was analyzed using one-way ANOVA followed by Tukey’s post hoc test. *ns* = not significant (*p* > 0.05)

INPs prepared using different inulin concentrations were compared in terms of particle size, size distribution, zeta potential, and redispersion behavior after lyophilization. The mean particle size decreased as the inulin concentration increased up to 50 mg/mL, reaching 204 ± 23 nm. The size increased to 671 ± 460 nm at 100 mg/mL and then decreased to 229 ± 20 nm at 200 mg/mL (**Figure 2A**). The 50 mg/mL formulation showed a narrow size distribution, whereas additional peaks above 2,000 nm were detected in the formulations prepared at 10 and 100 mg/mL (**Figure 2B**). The zeta potentials of INP and 5-ASA@INP remained approximately -30 mV, independent of the inulin concentration and the amount of encapsulated 5-ASA (**Figures 2C and 2F**). All samples prepared with different concentrations of inulin were evaluated for their rehydration behavior after lyophilization. At inulin concentrations of 100 mg/mL or higher, the samples were difficult to redisperse due to the high viscosity of the lyophilized mass (**Figure S1**). The 50 mg/mL formulation was readily redispersed, and the size distribution after rehydration exhibited a uniform profile. Therefore, the 50 mg/mL formulation was used for subsequent studies.

5-ASA was encapsulated into the nanoparticles during INP formation (**Figure 1**), and the physicochemical properties of 5-ASA@INP were analyzed via DLS. The particle size increased slightly with the initial 5-ASA feeding ratio, which was consistent with the quantitative increase in drug loading. L.E. and L.C were calculated for each 5-ASA feeding ratio. The L.E. remained consistently high (98.21%, 98.38%, 97.02%, and 99.08% for 1, 2, 5, and 10 wt% feeding ratios, respectively), while the L.C. increased proportionally to 0.98, 1.97, 4.85, and 9.91 wt%. These results indicate that the gradual increase in particle size at lower feeding ratios is attributed to the successful incorporation of an increased drug mass within the inulin matrix. However, at a 10 wt% feeding ratio, the particle size increased drastically to 5,900 ± 1329 nm, compared with those of particles loaded at lower drug concentrations (**Figure 2D**), suggesting significant particle aggregation despite the high encapsulation efficiency. Size distribution analysis indicated that all 5-ASA@INP formulations, except those containing 10 wt% 5-ASA, showed narrow size distributions with PDI < 0.3 (**Figures 2D-E**). The zeta potential was close to -30 mV for all 5-ASA@INP (**Figure 2F**). TEM imaging revealed spherical morphologies for both INP and 5-ASA@INP (**Figure 2G**). The residual DMSO content was 0.004% in INP and 5-ASA@INP (**Figure S3**). Among the formulations that retained physicochemical properties comparable to those of unloaded INP, the 5 wt% formulation exhibited the highest loading content. Therefore, the 5 wt% 5-ASA@INP was selected for subsequent experiments. For the selected 5 wt% formulation, additional FT-IR and UV-Vis analyses were performed to provide additional evidence for the incorporation of 5-ASA into the INPs (**Figure S4, S5**). These analyses supported the presence of 5-ASA in the nanoparticle formulation and were consistent with its physical encapsulation within the inulin matrix.

### Stability and drug release of 5-ASA@INP in the gastrointestinal tract

**Figure 3:**
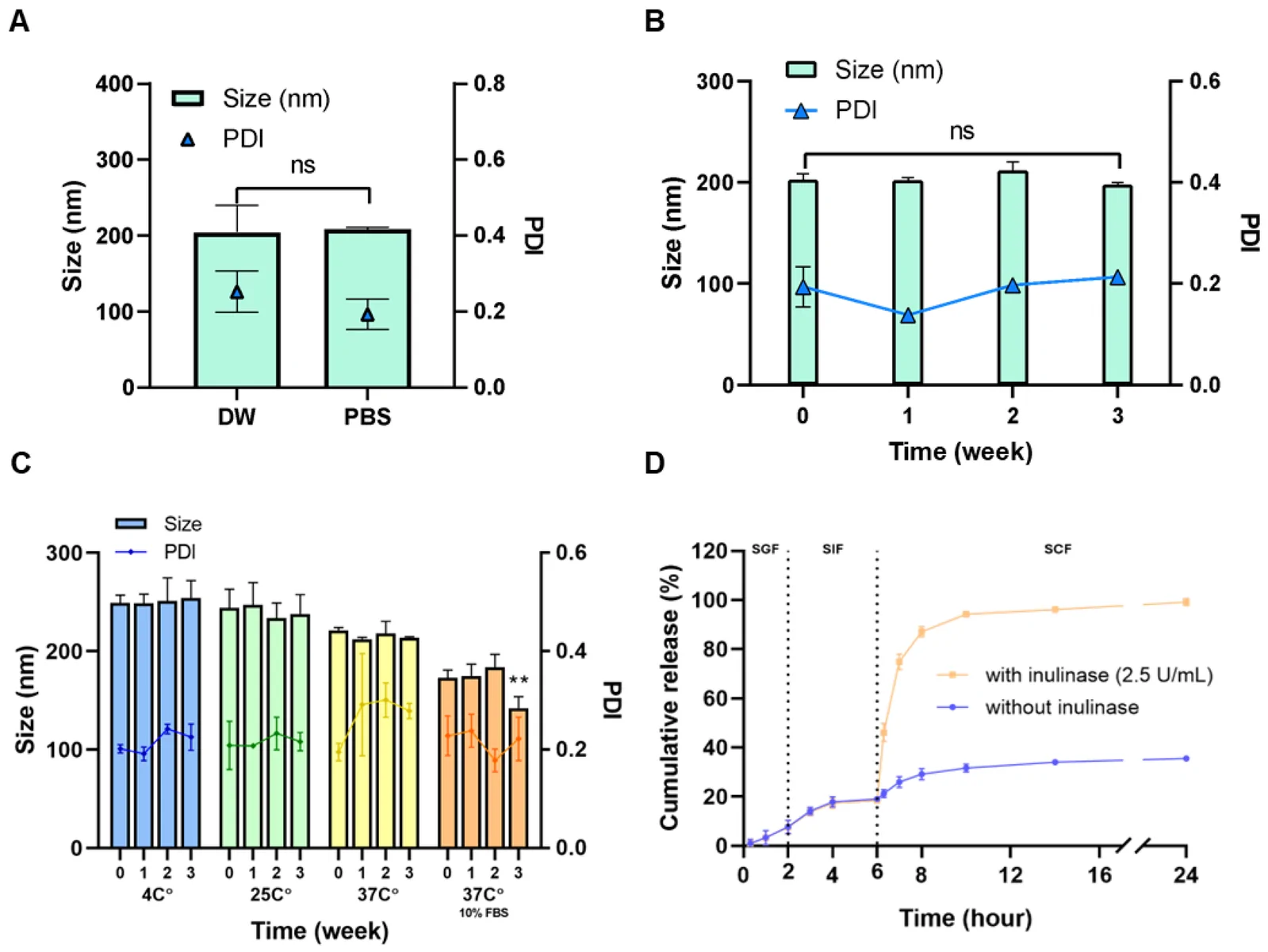
** Stability and drug-release profiles of INP and 5-ASA@INP.** (A) Hydrodynamic diameter (size) and polydispersity index (PDI) of INP redispersed in deionized water (DIW) and phosphate-buffered saline (PBS). (B) Stability of INP and (C) stability of 5-ASA@INP under various conditions (4 ℃, 25 ℃, 37 ℃, and 37 ℃ supplemented with 10% FBS) under constant stirring over 3 weeks. The 0-week time point represents the measurement taken after 1 hour of incubation in each respective condition to ensure environmental equilibration. (D) Sequential cumulative drug-release profiles of 5-ASA@INP in simulated gastric fluid (SGF, pH 1.2), simulated intestinal fluid (SIF, pH 6.8), and simulated colonic fluid (SCF, pH 7.4) with or without inulinase (2.5 U/mL). Data are presented as mean ± standard deviation (SD, n = 3). Statistical significance was analyzed using Student’s t-test for (A), and one-way ANOVA followed by Tukey’s post hoc test for (B) and (C), respectively. *ns* = not significant (*p* > 0.05), **p < 0.01

To evaluate the stability of INP and 5-ASA@INP under simulated physiological conditions, their particle sizes and PDIs were monitored. INP was first redispersed in DIW and PBS to compare its dispersion behavior. There was no significant difference in the particle size and PDI between the two media (**Figure 3A**). This uniform size distribution indicates that INP was well-dispersed and stabilized right after rehydration in both aqueous environments. Subsequently, the long-term stability of INP was examined by redispersing the nanoparticles in 5 mL of PBS and incubating them at 37 ºC with gentle shaking at 100 rpm for 3 weeks. To further assess the stability of 5-ASA@INP, the nanoparticles were stored under different temperature conditions (4 ºC, 25 ºC, and 37 ºC) and at 37 ºC supplemented with 10% FBS. Minor differences in the initial particle size among the storage groups were noted. Since the 0-week values were measured after 1 h of storage under each condition, these differences likely reflect early condition-dependent dispersion states arising from differences in temperature and medium composition, including the presence of serum proteins, rather than any intrinsic formulation inconsistency. The particle sizes of INP remained relatively stable throughout the incubation period, and the PDI of INP remained around 0.2 with minimal fluctuation (**Figure 3B**). In contrast, 5-ASA@INP maintained a relatively stable nanoscale size distribution under all tested storage conditions, although a decrease in particle size was observed at later time points under 37 ºC + 10% FBS condition. The PDI of 5-ASA@INP remained within a comparable range under the tested conditions. Although the PDI under the 37 ºC condition increased to approximately 0.3, this value remained within a generally stable colloidal range. In addition, the retained drug content of 5-ASA@INP remained at 80.36%, 78.26%, 79.13%, and 77.94% at 4 ºC, 25 ºC, 37 ºC, and 37 ºC + 10% FBS, respectively (**Figure S6**). Overall, these findings support the conclusion that both INPs and 5-ASA@INPs preserved their physicochemical stability under physiologically relevant conditions.

The cumulative release of 5-ASA from 5-ASA@INP was evaluated under conditions mimicking the sequential GI transit (**Figure 3D**). Drug release was monitored for 2 h in SGF, followed by 4 h in SIF, and then 18 h in SCF, for a total of 24 h. At the end of the release study, the solution remaining inside the dialysis device was collected to determine the total drug recovery. The total recovery of 5-ASA exceeded 95% in all groups, indicating negligible experimental loss. The cumulative release of 5-ASA reached 7.6 ± 2.7% after 2 h in SGF and gradually increased to 18.7 ± 0.95% after the subsequent 4 h in SIF, indicating limited premature drug release during upper GI transit. In the inulinase-containing SCF, cumulative 5-ASA release reached 46.0 ± 3.6% within 20 min and 87.0 ± 2.0% after 2 h. The results indicate rapid enzyme-triggered destabilization of the inulin matrix, leading to a cumulative release of 99.2 ± 1.5% at 24 h. In contrast, under the enzyme-free SCF condition, the cumulative release remained substantially lower, reaching only 35.6 ± 0.44% at 24 h. These findings demonstrate that 5-ASA@INP effectively restricts premature drug release under simulated gastric and intestinal conditions, while enzymatic degradation of inulin in the colonic environment triggers rapid and extensive drug release.

Consistent with these release results, DLS analysis under sequential GI conditions showed that 5-ASA@INP maintained its nanoscale size during the SGF and SIF stages. In contrast, upon exposure to SCF containing inulinase, both the apparent particle size and PDI increased markedly, suggesting enzyme-triggered destabilization of the inulin matrix (**Figure S7**). However, since DLS measurements in simulated GI fluids can be affected by salts and digestive components, these results served as supportive evidence and should be interpreted alongside the sequential release data.

### *In vitro* biological activity of INP

**Figure 4:**
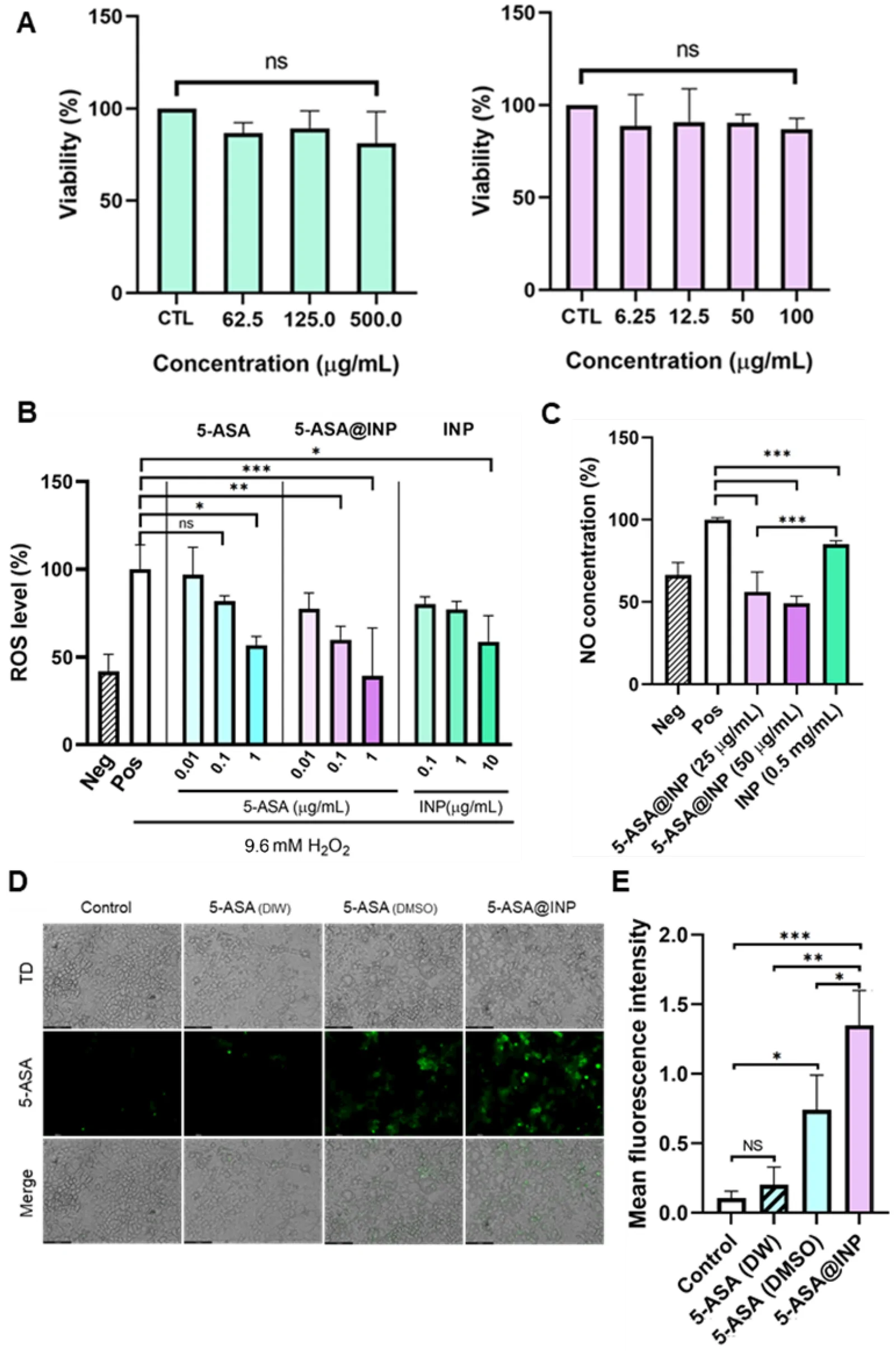
**Cytotoxicity, antioxidant, anti-inflammatory, and cellular uptake evaluation of 5-ASA@INP in Caco-2 cells**. (A) Cell viability of Caco-2 cells treated with INP (left) and 5-ASA (right). (B) ROS scavenging activity in H₂O₂-induced oxidative stress conditions. Caco-2 cells were treated with free 5-ASA (0.01-1 µg/mL), 5-ASA@INP (0.01-1 µg/mL, equivalent to 5-ASA), or INP alone (0.1-10 µg/mL). Bars are grouped left-to-right as free 5-ASA, 5-ASA@INP, and INP. “Neg” and “Pos” represent untreated and stimulated control groups, respectively. (C) Inhibition of NO production in LPS-stimulated Caco-2 cells treated with each formulation. “Neg” and “Pos” represent untreated and stimulated control groups, respectively. (D) Cellular association of free 5-ASA (DIW and DMSO) and 5-ASA@INP visualized by CLSM (Scale bar = 100 μm; magnification ×200). (E) Quantification of cell-associated 5-ASA fluorescence intensity. Data are presented as mean ± standard deviation (SD, n = 3). Statistical significance was analyzed using one-way ANOVA followed by Tukey’s post hoc test.* ns* = not significant, *p < 0.05, **p < 0.01, ***p < 0.001.

Cytotoxicity assays were conducted to determine the biocompatible concentration range prior to evaluating the biological activity of INPs. No significant cytotoxicity was observed in Caco-2 cells treated with INP (62.5–1,000 µg/mL) or 5-ASA (6.3–100 µg/mL) (**Figure 4A**). INPs also exhibited negligible cytotoxicity in NIH/3T3 cells at concentrations up to 5,000 µg/mL (**Figure S8**).

The antioxidant activity was evaluated under H₂O₂-induced oxidative stress by measuring the intracellular ROS levels in Caco-2 cells (**Figure 4B**). While free 5-ASA induced a modest reduction in ROS production, the values remained higher than those of the negative control even at 1 µg/mL. In contrast, 5-ASA@INP significantly reduced ROS levels in a concentration-dependent manner, with the highest concentration group exhibiting ROS levels comparable to those of the negative control. This enhanced effect was partly attributed to the intrinsic antioxidant properties of the INP, as evidenced by the ROS reductions observed following treatment with INP alone at equivalent concentrations (0.1, 1, and 10 µg/mL).

The anti-inflammatory effects of 5-ASA@INP were evaluated by measuring NO production in LPS-stimulated Caco-2 monolayers (**Figure 4C**). 5-ASA@INP at 25 and 50 µg/mL significantly reduced NO levels to 56.2% and 49.2% of the positive control, respectively (*p* < 0.001). The values were comparable to or lower than those of the negative control. INP alone also suppressed NO production relative to the positive control (*p* < 0.001). Similar antioxidant and anti-inflammatory results were observed in NIH/3T3 and RAW 264.7 cells, respectively (**Figure S9**), further supporting the enhanced therapeutic potential of 5-ASA when delivered via INP platform.

Cellular association of 5-ASA was visualized by CLSM (**Figure 4D**) and quantified based on its intrinsic fluorescence (**Figure 4E**). In addition, a standard fluorescence calibration curve of 5-ASA in PBS (pH 7.4) was established and is provided in **Figure S10** to confirm the linear relationship between concentration and fluorescence intensity under our experimental conditions. To evaluate the effect of solubility on cell-associated fluorescence, free 5-ASA was prepared in either DIW or DMSO at concentrations confirmed to be biocompatible. Among all groups, 5-ASA@INP demonstrated markedly higher cell-associated fluorescence intensity, indicating significantly enhanced cellular interaction. In contrast, free 5-ASA dissolved in DIW exhibited negligible fluorescence signals, which remained limited even when solubilized in DMSO. Quantitative analysis revealed that the cell-associated fluorescence of 5-ASA@INP was 12.82-fold and 1.95-fold higher than that of free 5-ASA dissolved in DIW and DMSO, respectively. These findings demonstrate that nanoparticle-mediated delivery increased the apparent cell-associated accumulation of 5-ASA under identical *in vitro* conditions. However, because the intrinsic fluorescence of 5-ASA can be influenced by the surrounding environment and this assay does not completely differentiate between membrane adsorption and true intracellular internalization, these results should be interpreted as relative MFI-based comparisons of cell-associated fluorescence between groups rather than as absolute quantification of intracellular internalization of 5-ASA.

### *In vivo* biodistribution

**Figure 5:**
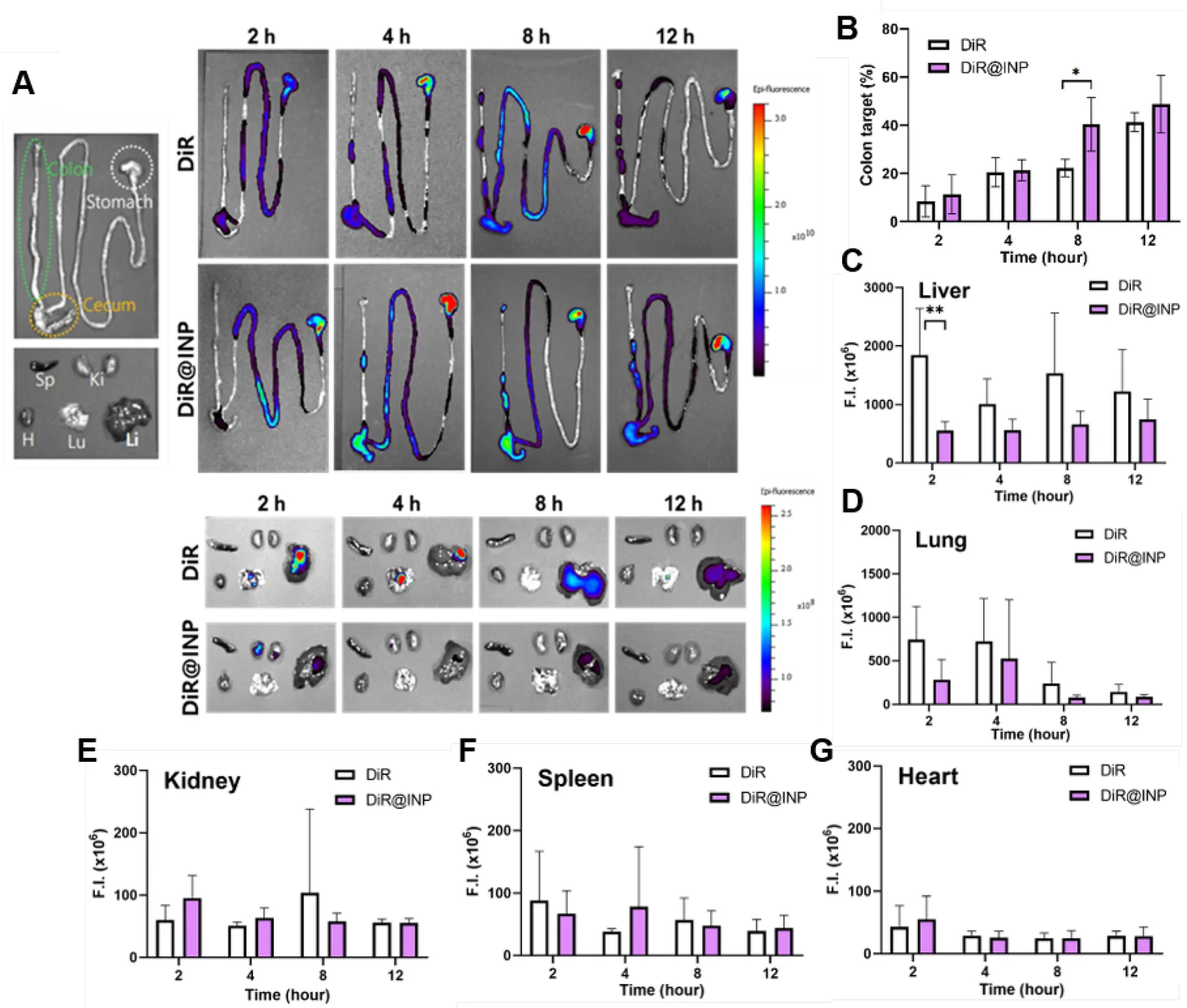
***In vivo*** **biodistribution of DiR@INP compared with free DiR following oral administration**. (A) Representative *ex vivo* fluorescence images of the gastrointestinal tract (GI) and major organs (liver, kidney, spleen, heart, and lung) collected at 2, 4, 8, and 12 h post-administration. (B) Quantitative analysis of the colon-to-total GI tract fluorescence intensity ratio. (C-G) Fluorescence intensity of the liver (C), lung (D), kidney (E), spleen (F), and heart (G) at each time point. IVIS imaging was performed using identical parameters (exposure time = 1 s; binning = medium). Data are presented as mean ± standard deviation (SD; DiR, n = 4; DiR@INP, n = 6 biologically independent mice per time point). Statistical significance was analyzed using two-way ANOVA followed by Sidak’s multiple comparison test. *p < 0.05, **p < 0.01, ***p < 0.001.

DiR@INP and free DiR were used to evaluate the *in vivo* biodistribution of the INP delivery system, following confirmation of the comparable physicochemical properties of INPs and DiR@INP (**Figure S11**). *Ex vivo* fluorescence images of the entire GI tract and major organs, including the kidneys, heart, spleen, and lungs, were acquired after oral administration to mice (**Figure 5A**). At 2 h post-administration, no detectable fluorescence was observed in the colon of either group, indicating that both formulations were still transitioning through the upper GI tract. From 4 h onward, the DiR@INP group exhibited a consistently stronger fluorescence intensity in the colon compared with the free DiR group. The ratio of colon-to-total GI tract fluorescence was higher in the DiR@INP group at all time points, with a statistically significant difference observed at 8 h (**Figure 5B**), demonstrating enhanced colonic retention.

To further assess distribution, fluorescence imaging of the major organs was conducted. At 2 h, pronounced fluorescence was detected in the liver of the free DiR group, suggesting rapid absorption of the dye through the upper GI tract (**Figure 5C**). In contrast, the DiR@INP group showed consistently lower fluorescence intensity in the liver at all time points. Similarly, the DiR@INP group exhibited minimal fluorescence in other major organs (**Figure 5D-G**), indirectly suggesting that the INP formulation helps mitigate the non-specific systemic absorption and off-target accumulation of 5-ASA.

### Therapeutic effect of 5-ASA@INP in a colitis animal model

**Figure 6:**
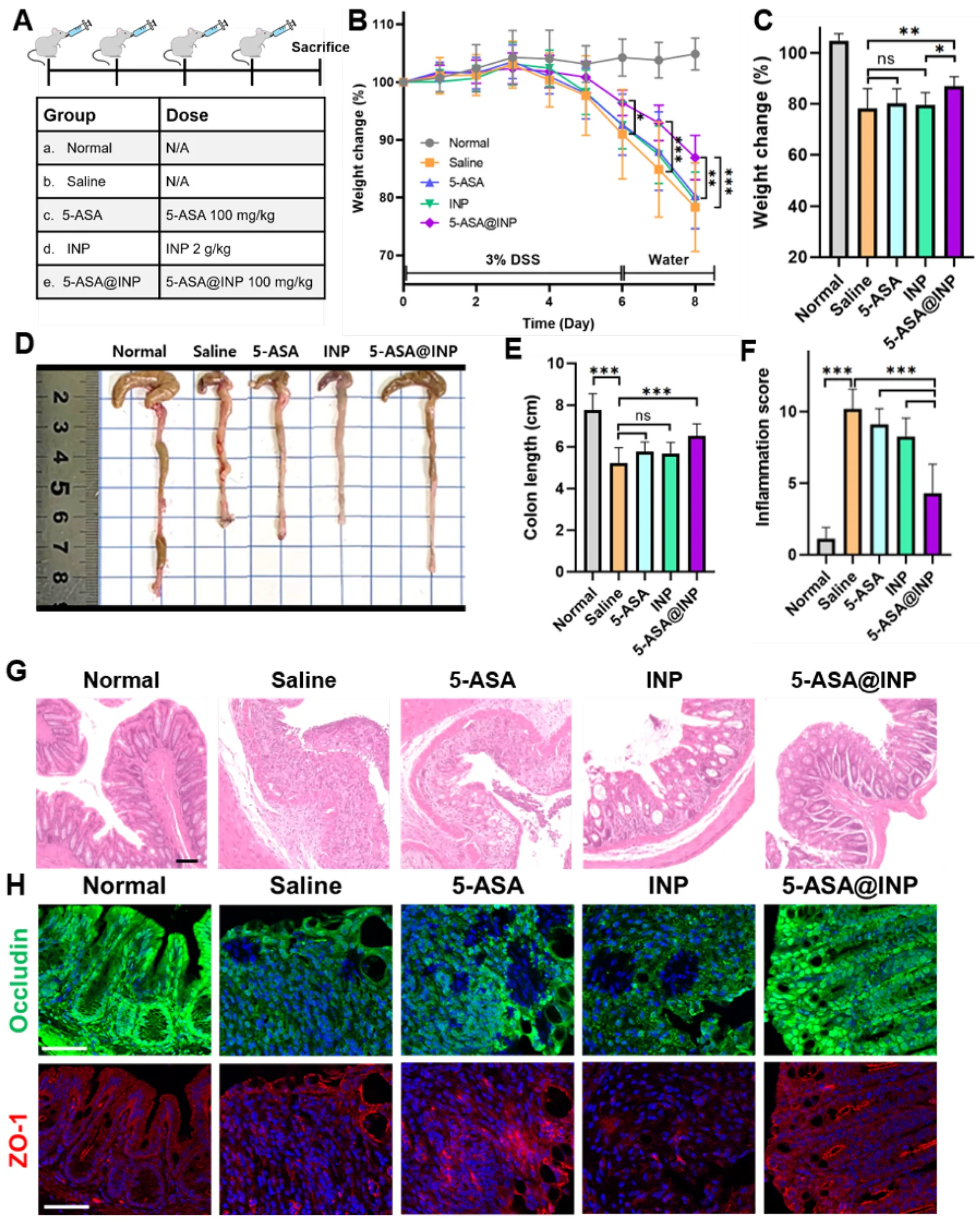
** Therapeutic efficacy of INP and 5-ASA@INP in a DSS-induced colitis mouse model.** (A) Schematic of the experimental design for DSS-induced colitis and treatment schedule. (B) Body-weight changes (%) during the 8-day treatment period. (C) Final body weight (%) on Day 8. (D) Gross morphology of the colon post-treatment and (E) colon length. (F) Quantitative inflammation scores based on histopathological evaluation. Data in (B), (C), (E), and (F) were obtained by combining two independent experimental cohorts and are presented as mean ± standard deviation (SD; normal, n = 8; saline, n = 11; 5-ASA, n = 9; INP, n = 8; 5-ASA@INP, n = 10 biologically independent mice). (G) Representative histological images (H&E staining) of colon sections from the confirmatory experiment (n = 5 per group; scale bars = 200 μm). (H) Representative immunofluorescence images of tight junction proteins in colon tissues from the confirmatory experiment (n = 5 per group): Occludin (green), ZO-1 (red), and nuclei counterstained with DAPI (blue) (scale bars = 50 μm). Body weight changes over time (B) were analyzed using two-way ANOVA followed by Sidak’s multiple comparison test. The endpoint body weight change (C), colon length (E), and inflammation score (F) were analyzed using one-way ANOVA followed by Tukey's post hoc multiple comparison tests. *ns =* not significant; *p < 0.05, **p < 0.01, ***p < 0.001.

The therapeutic effects of 5-ASA (100 mg/kg), INP (2 g/kg), and 5-ASA@INP (100 mg/kg and 2 g/kg) were evaluated in a DSS-induced colitis model (**Figure 6A**). On day 8, the body weight changes (%) relative to day 0 for the normal, saline, 5-ASA, INP, and 5-ASA@INP groups were 104.86%, 78.34%, 80.29%, 79.54%, and 86.93%, respectively (**Figure 6B**). The 5-ASA@INP group exhibited significantly less weight loss than the saline-treated group (*p* = 0.0067) (**Figure 6C**). The degree of inflammation-associated colonic shortening was also assessed (**Figure 6D**). Only the 5-ASA@INP-treated group showed significantly reduced colonic shortening compared to the saline-treated group (*p* = 0.0002) (**Figure 6E**). There were no significant differences in the colon weight-to-length ratio among all experimental groups (**Figure S14A**).

Histological evaluation of the inflammatory response in each group was performed using H&E-stained colonic sections (**Figure 6G**). In the saline-treated group, the colonic architecture was severely disrupted, with indistinct boundaries between the mucosa, submucosa, and muscularis mucosa, accompanied by substantial infiltration of immune cells. The 5-ASA- and INP-treated groups showed partial preservation of the mucosa compared with the saline group, however, the overall colonic structure remained largely disrupted. In contrast, the 5-ASA@INP-treated group exhibited a well-preserved colonic architecture, including intact goblet cells involved in mucus secretion. Morphological analysis was conducted using a histological scoring system (**Figure 6F**), based on five criteria: severity of inflammation indicated by leukocyte density in the lamina propria, extent of inflammation, presence of epithelial hyperplasia, crypt elongation, and presence of ulceration. The 5-ASA@INP-treated group exhibited significantly lower inflammation scores than the saline- (*p* < 0.0001), 5-ASA- (*p* < 0.0001), and INP-treated (*p* < 0.0001) groups. These morphological findings indicate that 5-ASA@INP reduced colonic inflammation. Furthermore, immunofluorescence staining of tight junction protein showed that occludin was better preserved in the 5-ASA@INP-treated group compared with the other treatment groups, indicating reduced colonic barrier disruption (**Figure 6H**). Notably, its expression was maintained at a level comparable to that of normal colonic tissue. Quantitative analysis of fluorescence intensity for the tight junction proteins is provided in the Supplementary Information (**Figure S14B and S14C**). Overall, 5-ASA@INP reduced colonic inflammation more effectively than free 5-ASA, supporting the ability of the INP system to limit premature drug release and improve colonic delivery.

**Figure 7:**
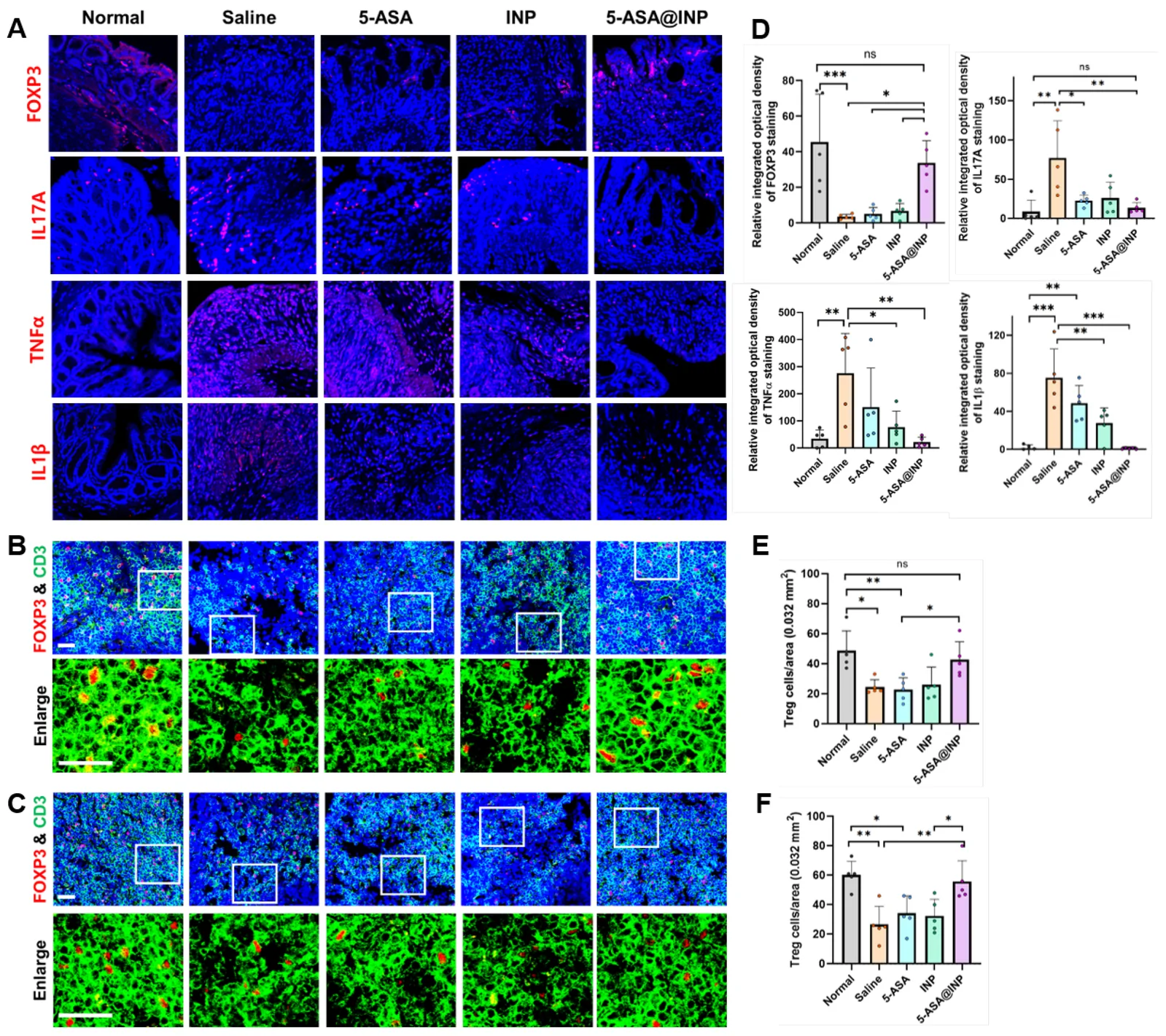
**Immunomodulatory effects of 5-ASA@INP on Treg/Th17 balance and pro-inflammatory cytokine expression in DSS-induced colitis mice.** (A) Representative immunofluorescence images of colonic sections stained for FOXP3, IL-17A, TNF-α, and IL-1β (Scale bars = 100 μm; magnification ×200). (B, C) Dual fluorescence staining of CD3 (green) and FOXP3 (red) in (B) Peyer’s patches (PPs) and (C) mesenteric lymph nodes (MLNs) (Scale bars = 25 μm; magnification ×400). (D) Quantitative analysis of the relative fluorescence intensity (RFI) for FOXP3, IL-17A, TNF-α, and IL-1β in colonic tissues. (E, F) Quantification of the number of CD3^+^FOXP3^+^ cells within a defined tissue area in (E) PPs and (F) MLNs. Data are presented as mean ± standard deviation (SD, n = 5 per group). Statistical significance was analyzed using one-way ANOVA followed by Tukey’s post hoc test.* ns* = not significant (*p >* 0.05), *p < 0.05, **p < 0.01, ***p < 0.001.

Immunofluorescence staining of colonic sections was used to examine FOXP3-positive cells and the inflammatory markers IL-17A, TNF-α, and IL-1β (**Figure 7A**). Quantitative analysis revealed that FOXP3 fluorescence intensity was significantly higher in the 5-ASA@INP group than in the saline- (*p* = 0.0154), 5-ASA- (*p* = 0.0230), and INP (*p* = 0.0351) groups (**Figure 7D**). Furthermore, IL-17A staining showed markedly reduced fluorescence intensity in the 5-ASA@INP group relative to the saline group (*p* = 0.0036), indicating Treg-Th17 balance restoration. Consistent with these findings, TNF-α and IL-1β expression followed a comparable trend, with both the INP and 5-ASA@INP groups exhibiting reduced cytokine levels compared with the saline group. Collectively, 5-ASA@INP alleviated colonic inflammation by enhancing Treg infiltration and suppressing pro-inflammatory cytokines, thereby contributing to improved therapeutic outcomes.

Treg responses in PPs and MLNs were examined by immunofluorescence staining to evaluate whether the observed Treg expansion in the colon is associated with immune regulation in gut-associated lymphoid tissues (GALT). Treg cells were identified by double immunofluorescence staining for CD3 (green) and FOXP3 (red), and the number of CD3^+^FOXP3^+^ cells was quantified within a defined area. The 5-ASA@INP-treated group significantly restored Treg cell levels in both PPs and MLNs to levels comparable to those of the normal group, whereas treatment with 5-ASA or INP alone resulted in only marginal increases compared with the saline group. These results suggest that 5-ASA@INP promotes immune regulation by enhancing Treg responses within the GALT.

### Restoration of gut microbiota composition and short-chain fatty acid production following 5-ASA@INP treatment

**Figure 8:**
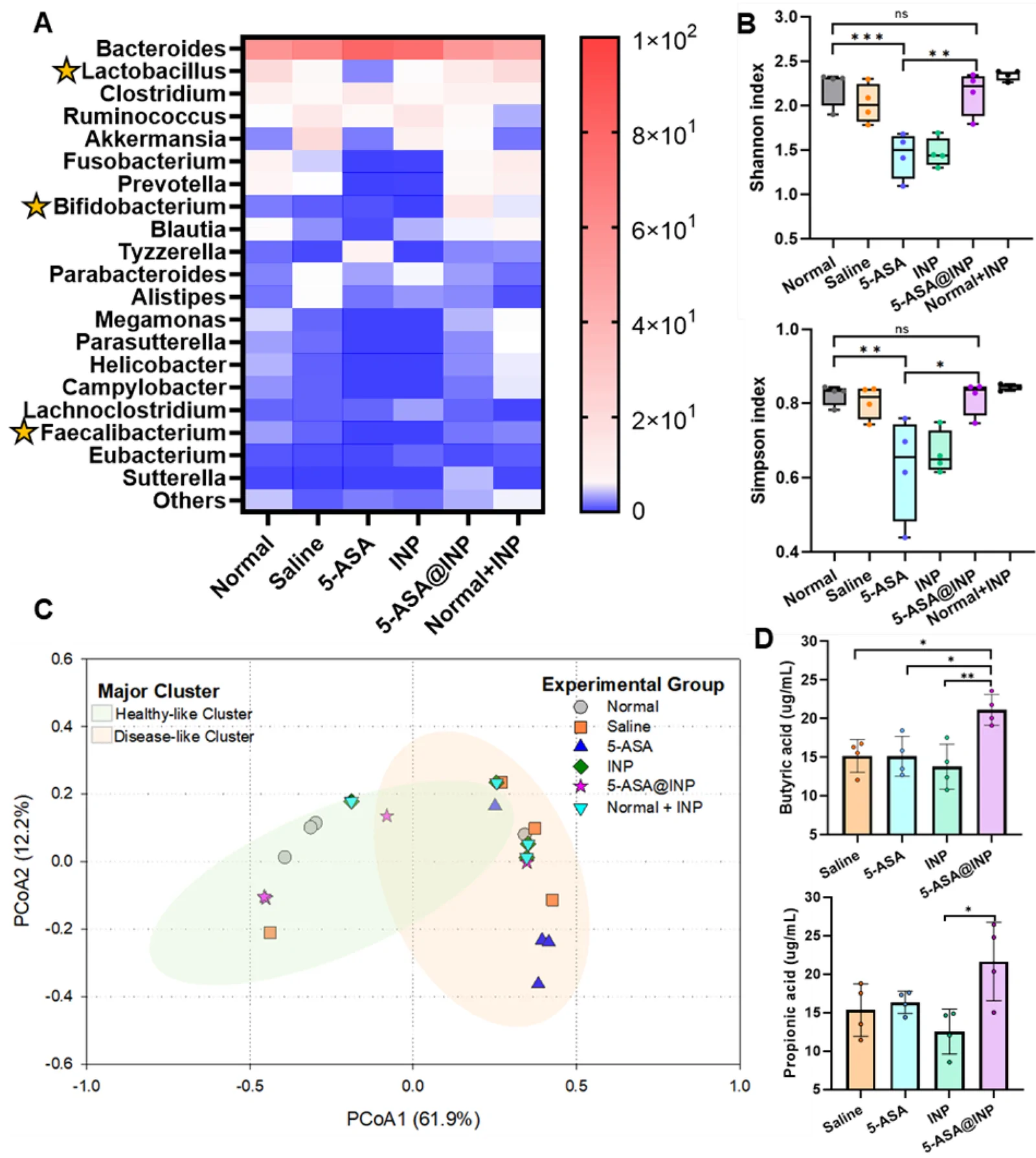
** Restoration of gut microbiota composition and short-chain fatty acid production following 5-ASA@INP treatment in a DSS-induced colitis mouse model**. (A) Analysis of microbial composition based on 16S rRNA sequencing data is shown as a heat map of the relative abundance at the genus level. Star symbols indicate representative beneficial bacterial genera associated with short-chain fatty acid (SCFA) production. (B) Alpha diversity of gut microbiota evaluated by Shannon and Simpson indices at the family level. (C) Principal coordinate analysis (PCoA) of beta diversity based on species-level. PERMANOVA analysis indicated significant differences in microbial community structure among groups (R² = 0.439, *p* = 0.01). Ellipses represent “healthy-like” (green) and “disease-like” (red) clusters. (D) Fecal concentrations of SCFAs (butyric acid and propionic acid) quantified by GC-MS. Data are presented as mean ± standard deviation (SD, n = 4 per group). Statistical significance was analyzed using one-way ANOVA followed by Tukey’s post hoc test.* ns* = not significant (*p >* 0.05), *p < 0.05, **p < 0.01, ***p < 0.001.

Gut microbial dysbiosis, a hallmark of IBD, is implicated in disease progression and complicates therapeutic outcomes. To investigate whether the therapeutic efficacy of 5-ASA@INP was mediated by restoration of gut microbiota driven by the intrinsic prebiotic properties of inulin, fecal microbial communities were analyzed using 16S rRNA sequencing across all experimental groups. An additional control group in which normal mice were administered inulin nanoparticles alone (Normal + INP) was included to confirm the intrinsic prebiotic effect of the INPs. At the genus level, relative microbial composition analysis revealed that several beneficial genera associated with SCFA production, including *Lactobacillus*, *Bifidobacterium*, and *Faecalibacterium*, were restored in the 5-ASA@INP-treated group to levels comparable to those observed in the normal group (**Figure 8A**). Species-level analysis further supported these findings. The representative probiotic species *Bifidobacterium longum* was reduced in the saline-treated group compared with the normal group, whereas its abundance partially recovered following 5-ASA@INP treatment (**Figure S24A**). Notably, administration of INPs alone to healthy mice increased the abundance of *B. longum*, indicating that inulin nanoparticles themselves exert probiotic-promoting effects. Similarly, the butyrate-producing bacterium *Faecalibacterium prausnitzii*, which is commonly depleted in IBD, showed increased abundance in the 5-ASA@INP-treated group compared with both the saline- and 5-ASA-treated groups (**Figure S24B**).

To evaluate microbial richness and evenness, alpha diversity was assessed using the Shannon and Simpson indices (**Figure 8B**). The 5-ASA group had significantly lower values than the normal group (Shannon,* p* = 0.0006; Simpson, *p* = 0.0079). Both indices were higher in the 5-ASA@INP group than in the 5-ASA group (Shannon,* p* = 0.0015; Simpson, *p* = 0.0108), while no significant difference was observed between normal and 5-ASA@INP groups. Additionally, the Normal + INP group showed similar indices to those of the normal group, suggesting that INP administration did not alter alpha diversity in healthy mice.

Beta diversity was examined to assess the overall microbial community using principal coordinate analysis (PCoA) based on Bray-Curtis distances. The first and second principal coordinates (PCoA1 and PCoA2) explained 61.9% and 12.2% of the total variance, respectively, and clearly separated the samples into two major clusters (**Figure 8C**). The ordination plot revealed a “healthy-like” cluster (green ellipse) and a “disease-like” cluster (red ellipse) on the left and right sides of the plot, respectively. Most samples from the saline, 5-ASA, and INP groups were located within the disease-like cluster. The samples from the normal and 5-ASA@INP groups mostly clustered within the healthy-like cluster. This distinct shift toward the healthy-like cluster suggests that 5-ASA@INP restored the microbial dysbiosis to a level comparable to the normal group. However, treatment with 5-ASA alone was insufficient to recover the normal microbial community.

Fecal butyric acid and propionic acid concentrations were measured using GC-MS (**Figure 8D**). Butyric acid levels were significantly higher in the 5-ASA@INP group than in the saline (p = 0.0210), 5-ASA (p = 0.0200), and INP (p = 0.0051) groups. This increase in butyric acid was consistent with the higher abundance of *F. prausnitzii* observed in the species-level analysis (**Figure S24B**). The concentration of propionic acid was also observed to be higher in the 5-ASA@INP group, compared with the other groups. These findings indicate that 5-ASA@INP treatment was associated with increased SCFA production by promoting the enrichment of SCFA-producing bacteria.

## Discussion

5-ASA is widely used in the clinic, yet it has several pharmacological limitations, such as poor aqueous solubility and extensive first-pass metabolism 30,31. These limitations restrict 5-ASA accumulation at therapeutically relevant concentrations in inflamed colonic tissues and often necessitate high doses and frequent administration, thereby reducing patient compliance and increasing the risk of systemic side effects. Consequently, there is a pressing need for advanced drug delivery systems that enhance colonic targeting, improve bioavailability, and reduce systemic toxicity. The 5-ASA@INP formulation addresses these challenges through a microbiota-responsive, colon-targeted delivery strategy. INPs are selectively degraded by bacterial inulinase present in the colon, enabling site-specific release of 5-ASA. This mechanism minimizes premature drug absorption in the upper GI tract and ensures targeted delivery to the inflamed regions, thereby improving therapeutic efficacy and reducing systemic exposure.

In addition to its role as a targeted drug carrier, the 5-ASA@INP system offers dual therapeutic benefits by incorporating the bioactive properties of inulin. INPs alone demonstrated antioxidant and anti-inflammatory effects, suggesting a combined therapeutic contribution with 5-ASA to mitigate mucosal inflammation. Furthermore, as a prebiotic, inulin promotes the proliferation of short-chain fatty acid-producing beneficial gut bacteria, such as *Faecalibacterium prausnitzii* 32 and *Bifidobacterium longum* 33, while suppressing colitis-associated genera, including *Turicibacter* and *Odoribacter* 34. In the present study, treatment with 5-ASA@INP significantly restored the abundance of beneficial gut microbial genera associated with SCFA production, including *Lactobacillus*, *Bifidobacterium*, and *Faecalibacterium*. Species-level analysis further revealed an enrichment of the probiotic bacterium *B. longum* and the butyrate-producing bacterium *F. prausnitzii* following 5-ASA@INP treatment. These microbiota changes were accompanied by significantly increased fecal levels of butyric and propionic acids, indicating enhanced microbial metabolic activity. SCFAs serve not only as metabolic substrates but also as immunomodulatory agents that suppress the growth of pathogenic bacteria by lowering the colonic pH 35,36. Among them, butyrate plays a key role in regulating host immune responses by promoting the differentiation of Treg cells, maintaining tight junction integrity, and suppressing the expression of inflammatory genes through inhibition of histone deacetylase (HDAC) 37. The increased SCFAs production in the 5-ASA@INP-treated group may thus have led to the enhanced Treg responses and improved intestinal barrier function observed in this study. In addition, inulin is generally recognized as safe (GRAS) 35 and offers both biocompatibility and biodegradability. These properties make it suitable for long-term use in IBD treatment. In agreement with these characteristics, histological evaluation (H&E staining), serum biochemical analyses (ALT, AST, BUN, and creatinine), and hemolysis assays showed excellent biocompatibility without detectable tissue damage or systemic toxicity (**Figure S26 and S27**).

In addition to inulin, other dietary fibers such as chitosan, pectin, and alginate exhibit pH responsiveness, indigestibility, and fermentation potential. Numerous studies have developed colon-targeting oral drug delivery systems using such dietary fibers and strategies (Table 1). Sun et al. used the ionic gelation method and developed alginate/chitosan nanoparticles (Ag-Cs NP) for the delivery of berberine to treat UC. Ag-Cs NP exhibited strong electrostatic interactions, with a loading efficiency (L.E.) of 83.6% and a loading content (L.C.) of 12.4 wt%. In SGF, the protonation of the carboxyl groups of alginate enhanced hydrogen bonding with chitosan. In SIF, the carboxyl groups of alginate remained bound to chitosan, preventing the release of berberine. However, in SCF, ionization of the carboxyl groups led to the release of 70% of the total berberine and 77.8% colonic berberine, indicating effective protection and colon-targeting delivery 38. Wu et al. used a desolvation and EDC/NHS coupling method to synthesize chitosan/pectin-based BSA nanoparticles (CP NP) for the delivery of tofacitinib in colitis therapy. CP NP achieved an L.C. of 30.9 wt% and an L.E. of 49.7%. In that study, acidic conditions protonated the carboxyl groups of pectin, enhancing its hydrogen bonding with chitosan. Under simulated colonic conditions, the slightly basic pH and the presence of bacterial enzymes triggered the dissociation of pectin from chitosan, releasing 58% of the total tofacitinib, 92% of which reached the colon. The dissociated pectin was subsequently fermented by the gut microbiota, contributing to its prebiotic effects 39. Cheng et al. developed alginate-protamine microcapsules (AP) as oral probiotic carriers using an electrostatic droplet method with alginate and protamine layers assembled via a layer-by-layer technique. AP remained stable in SGF, whereas in SIF, the protamine layers were enzymatically degraded by trypsin, exposing the alginate layers for further degradation in SCF. AP achieved 40% release of encapsulated probiotics 40. Cai et al. synthesized curcumin-loaded pectin gel beads via calcium-induced gelation, achieving an L.E. of 40% and an L.C. of 3 wt%. Calcium ions bound strongly to pectin and formed an “egg-box” network. This structure remained intact under upper GI conditions. The release profile showed that curcumin remained stable in SGF and SIF. In SCF, degradation by pectinase led to a 50.6% release of curcumin 41. Lv et al. synthesized hyaluronic acid-chitosan/Eudragit S100/PLGA nanoparticles (hCEP) for the colonic delivery of methotrexate (MTX). MTX@hCEP was fabricated using an emulsion-based method, followed by layer-by-layer surface modification, achieving an L.E. of 50.6% and an L.C. of 6.3 wt%. The release profile of MTX@hCEP exhibited a typical pH-dependent pattern, with 13.5% of MTX released in SGF and SIF, and up to 76.8% in SCF, corresponding to 73.18% of colonic MTX release 42. Hufnagel et al. developed 5-ASA-Acetylated inulin pellets (5-ASA-InAc) by levigating the components and dispersing the mixture in DMSO, followed by stirring in DW to induce aggregation. 5-ASA-InAc achieved an L.E. of 92% and an L.C. of 54.6%. During transit through SGF and SIF, only 17.4% of 5-ASA was released, while 75.9% of the remaining drug was released in SCF 23.

A comparison with previously reported dietary fiber-based colon-targeted systems highlights several distinguishing features of our 5-ASA@INP platform beyond the performance differences summarized in Table 1. While various systems have shown potential, some remain limited by relatively low drug-loading efficiency or incomplete colonic release. Unlike multi-component or layer-by-layer systems, 5-ASA@INP is based on a single dietary polysaccharide matrix, which may offer advantages in formulation simplicity, reproducibility, and manufacturing scalability 43,44. Its nanoscale size also distinguishes it from larger gel bead- or pellet-type carriers and may facilitate more efficient enzymatic access and drug diffusion under colonic conditions 45,46. Furthermore, because drug release from 5-ASA@INP is governed primarily by microbiota-responsive enzymatic degradation rather than luminal pH, this system may retain its functionality under the altered pH conditions commonly observed in IBD 42,47. These structural and mechanistic features are likely to contribute to the high drug-loading efficiency (97.02%) and nearly complete colonic release (99%) observed in this study. Moreover, inulin is a well-established prebiotic substrate that is readily fermented by colonic microbiota to produce SCFAs, including butyrate 48-50. These metabolites support epithelial integrity and immune regulation, thereby providing additional biological value beyond its role as a passive drug carrier 51. Collectively, these findings suggest that 5-ASA@INP is a structurally simple, microbiota-responsive, and biologically active platform for effective colon-targeted drug delivery. It should be noted, however, that the inulinase-containing simulated colonic fluid used in this study represents a comparative *in vitro* model and does not fully reproduce the complexity of the *in vivo* colonic enzymatic environment. Therefore, the release results should be interpreted as supportive evidence of microbiota-responsive degradability rather than as a direct quantitative predictor of *in vivo* release kinetics.

In line with this rationale, recent work evaluating orally administered inulin-based colonic delivery systems has similarly employed inulinase-responsive release behavior together with cecum/colon localization and retention as key readouts 52. In this context, our findings provide a mechanistic linkage between the specific formulation design of 5-ASA@INP, its enzyme-triggered behavior *in vitro* and its preferential localization in the lower intestine *in vivo*. This multi-level validation effectively highlights the advantages of our inulin-based nanoparticles in achieving microbiota-responsive colonic targeting while minimizing premature drug loss in the upper GI tract, thereby supporting the overall mechanistic rationale of our delivery platform.

Even so, certain limitations remain in the present *in vitro* model. While our current findings clearly demonstrate enhanced cellular accumulation efficiency, we have not yet identified which endocytic pathways drive this internalization. Nanoparticles are typically internalized via multiple parallel mechanisms, including clathrin-mediated endocytosis, caveolae/lipid raft-mediated endocytosis, macropinocytosis, and phagocytosis. Future studies should systematically investigate these pathways using pathway-specific chemical inhibitors 53,54. Furthermore, the present cellular uptake model should be regarded as a simplified *in vitro* system focused on comparing formulation-dependent interactions rather than a complex representation of the microbiota-driven colonic environment *in vivo*. In addition, the cell model used in this study was a conventional two-dimensional Caco-2 culture system, which is useful for initial cell-level screening but could not sufficiently recapitulate the pathological environment of the disease. Therefore, LPS stimulation was applied to induce inflammatory conditions. Moreover, this model has inherent limitations in reproducing the *in vivo* microenvironment and the actual exposure concentrations of the nanoparticles and drug. This limitation restricts direct interpretation of the relationship between *in vitro* and *in vivo* findings. Future studies using more physiologically relevant *in vitro* models, such as intestinal organoids, may help improve the evaluation of microbiota-responsive and colon-targeted delivery systems 55.

The GI tract is a complex environment in which the immune system, intestinal barrier, and gut microbiota interact, shaping both local and systemic disease outcomes 56-58. Approximately 70% of the body’s lymphocytes reside in the GI tract, which is broadly divided into inductive and effector sites. Inductive sites include GALT, such as PPs and MLNs, while the main effector site, the lamina propria, contains T cells, plasma cells, and antigen-presenting cells 59. Under constant antigen exposure, the GI tract maintains immune homeostasis through both defensive responses and immunosuppressive mechanisms involving Treg, IL-10, transforming growth factor-beta (TGF-β), and retinoic acid 60. Among the inductive sites within GALT, PPs in the small intestine play a pivotal role in initiating adaptive immune responses 61. Within the PPs, the overlying M cells detect luminal antigens and deliver them to the underlying dendritic cells (DCs). The DCs then activate T cells and drive plasma cell differentiation, leading to strong immunogenic responses 62. Therefore, PPs are considered suitable targets for oral vaccine delivery, where inducing immunogenicity is prioritized over immune tolerance. The lamina propria, by contrast, plays a critical role in maintaining immune homeostasis through pathogen defense and immune tolerance 60,63. Pattern recognition receptors (PRRs) in the intestinal epithelium detect pathogens and initiate innate immune responses to protect the host. Ly6C^high^ monocytes mediated early-stage defense by differentiating into CX3CR1^int^ macrophages, which activate inflammatory signals such as TNF-α and recruit neutrophils. Neutrophils eliminate pathogens via mechanisms involving ROS and antimicrobial peptides. However, excessive accumulation of inflammatory immune cells and overproduction of TNF-α can exacerbate IBD 64. Furthermore, increased bacterial exposure resulting from disruption of the mucus layer, impairment of epithelial tight junctions, and dysfunction of Paneth cells further aggravate IBD by promoting the production of pro-inflammatory cytokines such as IL-6, IL-23, IL-17, and IFN-γ 5. Therefore, inducing immune tolerance is essential for preventing excessive immune responses. In the lamina propria, the Ly6C^high^ monocytes differentiate into anti-inflammatory CX3CR1^high^ macrophages, while resident CD103⁺ dendritic cells promote the differentiation of Tregs through IL-10 and retinoic acid, and contribute to maintaining immune homeostasis via TGF-β secretion 65-67. Accordingly, targeting the lamina propria is a promising strategy for oral nanomedicines to induce immune tolerance. In summary, the GI tract functions as a complex immune organ, in which GALT and lamina propria play distinct but complementary roles in immune defense and homeostasis, respectively.

## Conclusions

In the present study, we developed 5-ASA-loaded inulin nanoparticles to enhance colon-targeted delivery for the oral treatment of UC, using DiR fluorescence as an indirect indicator to trace the colonic accumulation of the nanocarriers. We used inulin, a GRAS dietary fiber, as a platform for oral 5-ASA delivery. Its biocompatibility and biodegradability have the potential to overcome the limitations associated with conventional 5-ASA treatment. We characterized the physicochemical properties of the nanoparticles, confirming their nanoscale size, colloidal stability, and high drug-loading performance. The developed 5-ASA@INP exhibited colon-responsive drug release under simulated gastrointestinal conditions. *In vitro* studies demonstrated good biocompatibility and bioavailability, including ROS-scavenging and anti-inflammatory effects. *In vivo*, the targeted biodistribution, therapeutic efficacy, and microbiota-related effects of 5-ASA@INP were assessed in a mouse model of DSS-induced colitis. 5-ASA@INP showed preferential localization in the lower intestine and significant therapeutic efficacy, accompanied by reduced pro-inflammatory cytokine expression and modulation of inflammatory responses. Furthermore, 5-ASA@INP contributed to the restoration of the beneficial gut microbiota and increased SCFA levels, further supporting the added biological value of the inulin-based delivery platform. Taken together, these results suggest that 5-ASA@INP is a promising platform for the colon-targeted therapy of UC. To clarify the mechanisms underlying the therapeutic benefits of 5-ASA@INP, the relationships among inulin fermentation, SCFA production, gut microbiome changes, and their combined effect with 5-ASA should be investigated. Beyond these, for future clinical translation, several further studies should be conducted. These include extended stability studies, long-term safety evaluation, direct comparison with currently approved oral 5-ASA products, and more advanced preclinical validation.

## Supplementary Material

Supplementary experimental procedures, full datasets, and supporting figures that provide further validation of the results presented in the main manuscript.

## Figures and Tables

**Table 1:** Therapeutic potential of various dietary fiber-based colon targeting carriers. The colon release efficiency (%) is calculated from (amount of drug released in colon / amount of drug remaining in the carrier at the time of colon entry) × 100.

Type of model disease	Dietary fiber	Carrier	Drug	Size	PDI	Zeta potential (mV)	Loading efficiency (%)	Colon targeting strategy	Colon release efficiency (%)	Reference
UC	Alginate, Chitosan	Alginate-chitosan nanoparticle	Berberine	257 ± 3.5 nm	0.166 ± 0.02	-39.1	83.6	pH-dependent	77.8	38
UC	Chitosan, Pectin	BSA-chondroitin sulfate-chitosan/pectin nanoparticle	Tofacitinib	308.1 ± 4.8 nm	0.065	-23.1	49.7	pH-dependent, Enzymatic degradation	30.56	39
Dysbiosis	Alginate	Alginate/Protamine microcapsule	Escherichia coli MG1655	800 ± 200 μm	N/A	N/A	N/A	Enzymatic degradation	50	40
N/A	Pectin	Pectin gel bead	Curcumin	2 mm	N/A	N/A	40	Enzymatic degradation	50.66	41
IBD	Chitosan	Hyaluronate-chitosan/Eudragit S100/PLGA nanoparticle	Methotrexate	202.4 ± 3.8 nm	0.10 ± 0.01	-18.7	50.6	pH-dependent	73.18	42
IBD	Inulin	Acetylated inulin pellet	5-ASA	Few millimeters	N/A	N/A	92	Enzymatic degradation	75.93	23
IBD	Inulin	Inulin nanoparticles	5-ASA	236.5 ± 20.7 nm	0.276 ± 0.005	-29.9	97.02	Enzymatic degradation	99.02	This study

**Abbreviations:** UC: ulcerative colitis; IBD: Inflammatory bowel disease; 5-ASA: 5-aminosalicylic acid; PDI: polydispersity index; N/A: not applicable.

## Data Availability

The data that support the findings of this study are available from the corresponding author upon reasonable request.

## Abbreviations

5-ASA: 5-Aminosalicylic acid
5-ASA@INP: 5-ASA-loaded inulin nanoparticle
CCK-8: Cell Counting Kit-8
CLSM: Confocal laser scanning microscopy
DLS: Dynamic light scattering
DiR: 1,1'-Dioctadecyl-3,3,3',3'-tetramethylindotricarbocyanine iodide
DMSO: Dimethyl sulfoxide
DMEM: Dulbecco’s modified Eagle’s medium
DIW: Deionized water
DSS: Dextran sulfate sodium
FBS: Fetal bovine serum
GI: Gastrointestinal
GRAS: Generally recognized as safe
H&E: Hematoxylin and eosin
IBD: Inflammatory bowel disease
IFN-γ: Interferon-gamma
IL: Interleukin
INP: Inulin nanoparticle
JAK: Janus kinase
L.C.: Loading content
L.E.: Loading efficiency
LPS: lipopolysaccharide
MFI: Mean fluorescence intensity
MTT: 3-(4,5-Dimethylthiazol-2-yl)-2,5-diphenyltetrazolium bromide
NF-κB: Nuclear factor kappa B
NO: Nitric oxide
PDI: Polydispersity index
PBS: Phosphate-buffered saline
PS: Penicillin-streptomycin
RFI: Relative fluorescence intensity
ROI: Region of interest
ROS: Reactive oxygen species
SCF: Simulated colonic fluid
SCFAs: Short-chain fatty acids
SD: Standard deviation
SIF: Simulated intestinal fluid
SGF: Simulated gastric fluid
TNF-α: Tumor necrosis factor-alpha
Treg: Regulatory T cell
TSA: Tyramide signal amplification
UC: Ulcerative colitis
ALT: Alanine aminotransferase
AST: aspartate aminotransferase
BUN: blood urea nitrogen
Crea: Creatinine
