# Supplementary material for: Microbial enzyme-responsive inulin nanoparticles for colon-targeted oral delivery of 5-ASA toward improved therapy of inflammatory bowel disease
Source: Theranostics. 2026 Aug 24;16(15):8892–913. doi: 10.7150/thno.127560 (PMC13549376; doi:10.7150/thno.127560)
Supplement: Supplementary file 1 [file thnov16p8892s1.pdf]

Supporting Information

Microbial Enzyme-Responsive Inulin Nanoparticles for Colon-Targeted Oral Delivery of 5-ASA toward Improved Therapy of Inflammatory Bowel Disease

Haena Park[#], Panmo Son[#], Chiwoo Oh[#], Hyeryeon Oh, Jee-Eun Hwang, Kim Seo Young, Jeongcheol Lee, Jae Kyoo Lee, Won Il Choi, and Hyung-Jun Im**

In vitro cytotoxicity and bioactivity of INP

NIH-3T3 fibroblast cells were cultured in a mixture containing DMEM supplemented with 10% FBS and 1% PS at 37 °C in a humidified atmosphere containing 5% CO_2. The cytotoxicity, ROS scavenging, and anti-inflammatory activity of INP were examined via CCK-8, H2DCFDA, and NO assays following previously reported methods. Cells were seeded into 96-well plates at a density of 1×10^4 cells per well and incubated for 24 h at 37 °C under 5% CO_2. For cytotoxicity tests, INP was added at concentrations ranging from 50 to 5,000 μg/mL and incubated for 24 h. The supernatants were then removed and replaced with 10% CCK-8 containing medium. After 0.5 h, the absorbance of the medium in the wells was measured using a microplate reader at 450 nm. For ROS scavenging evaluation, cells were treated with INP at concentrations ranging from 1 to 1,000 ng/mL with 10 μM H_2O_2. After 4 h, the supernatants were removed and replaced with PBS containing H2DCFDA. After 20 min of incubation, the fluorescence of the medium in the wells was analyzed using a microplate reader at ex = 490 and em = 530 nm. For the NO assay, cells were treated with INP at ranging concentrations from 1 to 100 ng/mL along with 100 ng/mL LPS. After 24 h, the supernatants were removed and replaced with Griess reagent. After incubation for 10 min, the absorbance of the medium in the wells was measured at 560 nm using a microplate reader.

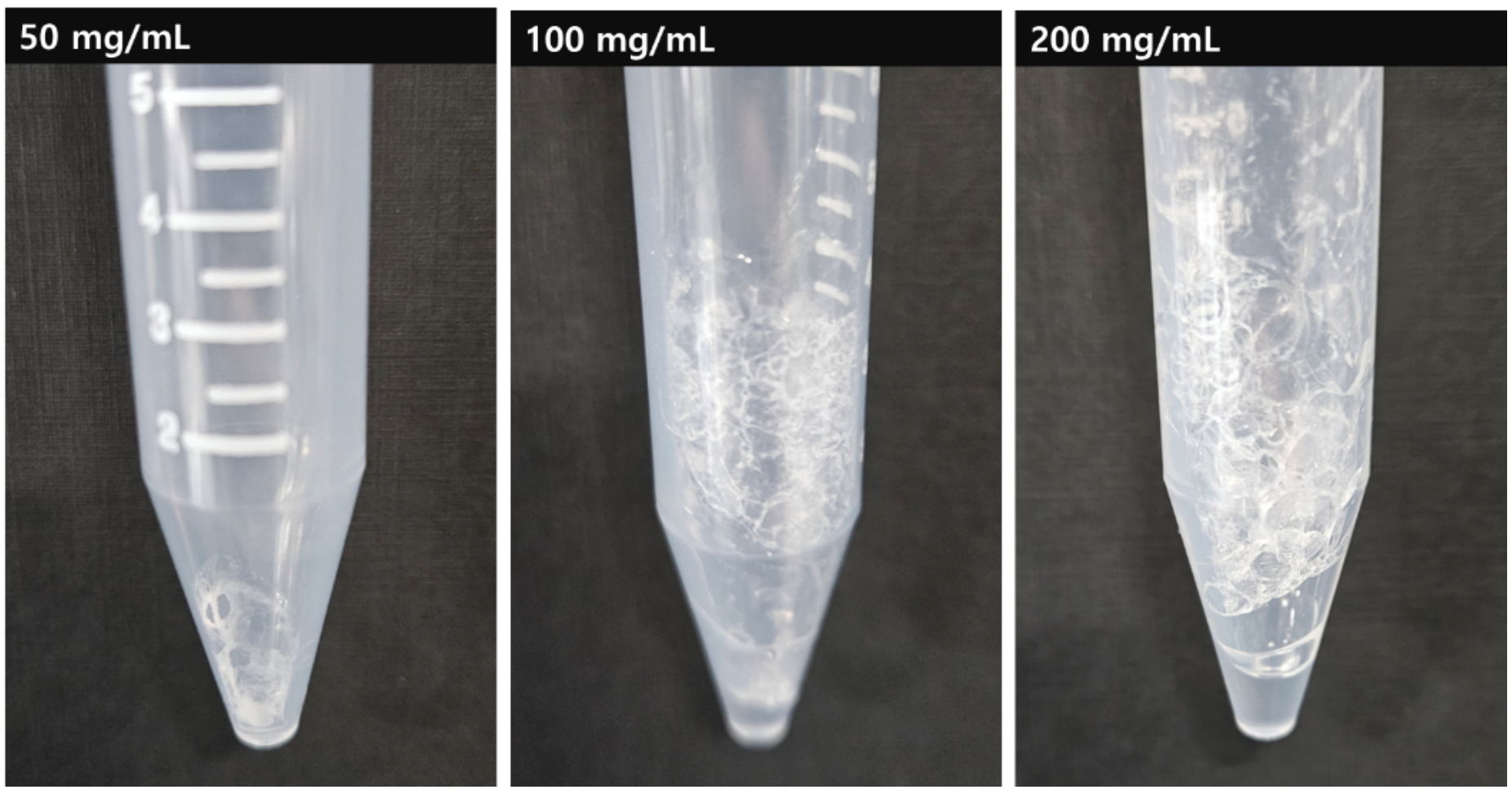

Figure S1. **Photographs of lyophilized INP prepared at various inulin concentrations (50, 100, and 200 mg/mL).** Higher inulin concentration produced sticker, more viscous samples, and foam-like structures and aggregates during lyophilization.

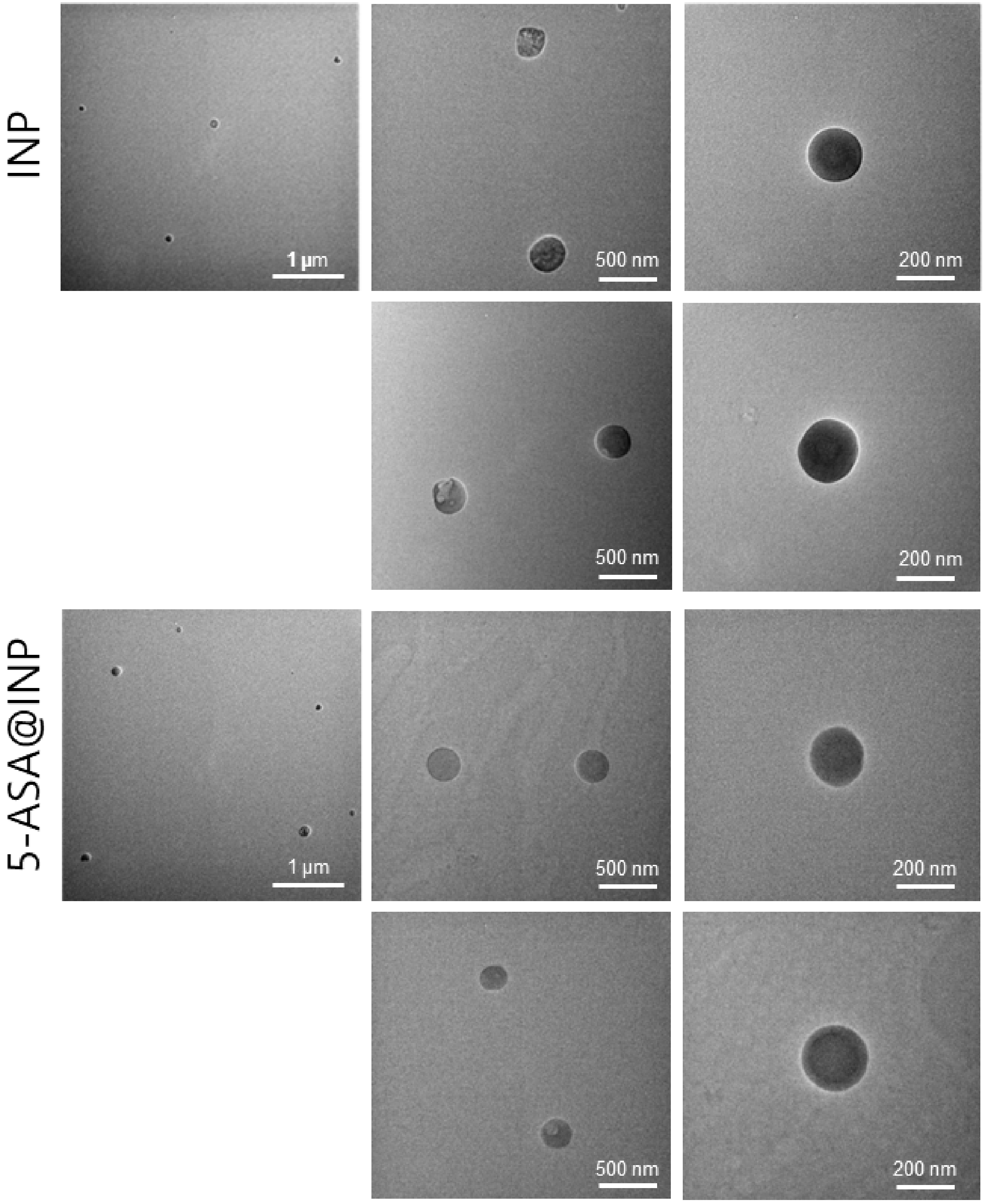

Figure S2. Representative TEM images of INP and 5-ASA@INP at different magnifications. The images support the spherical morphology and overall consistency of the nanoparticle formulation, while bulk size distribution was assessed by DLS. Scale bars = 1 μm, 500 nm, and 200 nm.

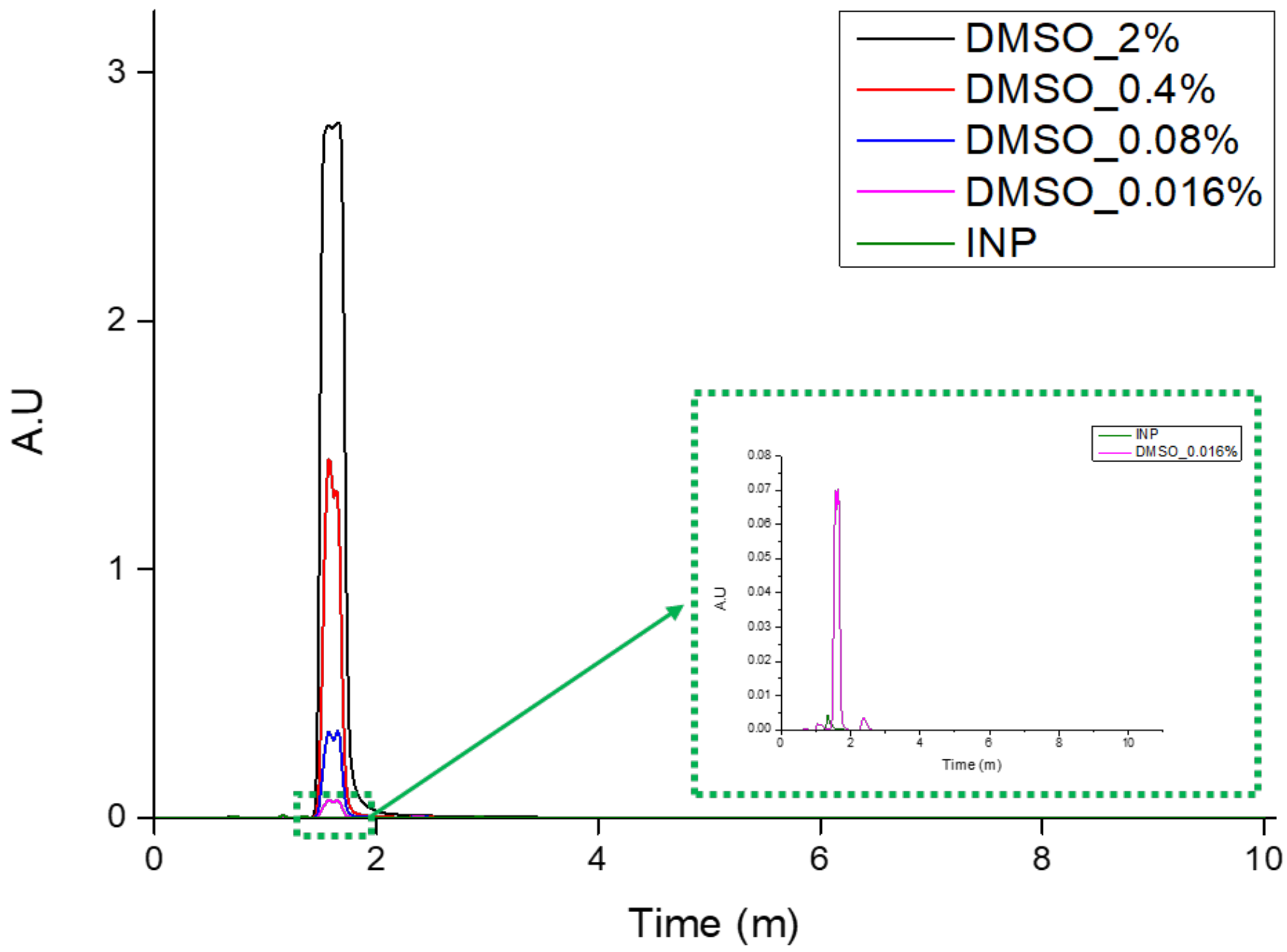

Figure S3. Residual DMSO concentration in INP analyzed via high-performance liquid chromatography. Chromatograms of DMSO standards (2%, 0.4%, 0.08%, and 0.016%) and INP are shown. The residual DMSO content in INP and 5-ASA@INP was determined to be 0.004%.

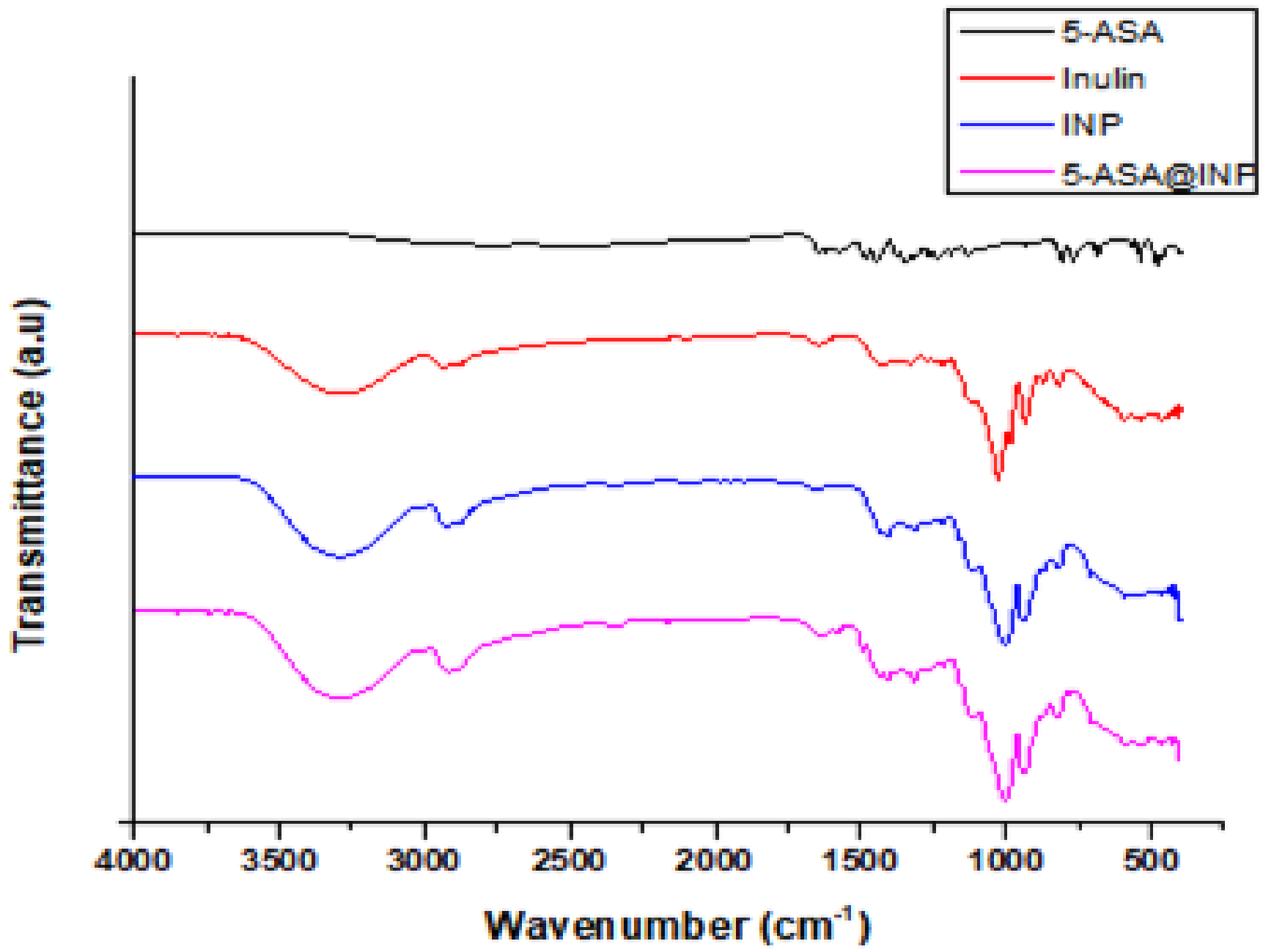

Figure S4. **FT-IR spectra of 5-ASA, Inulin, INP, 5-ASA@INP.** The FT-IR spectra of INP and 5-ASA@INP display the characteristic peaks of inulin at approximately 3300 cm^{-1} (O-H stretching) and 1030 cm^{-1} (C-O-C stretching). The spectrum of 5-ASA@INP contains overlapping absorption bands corresponding to both 5-ASA and inulin components.

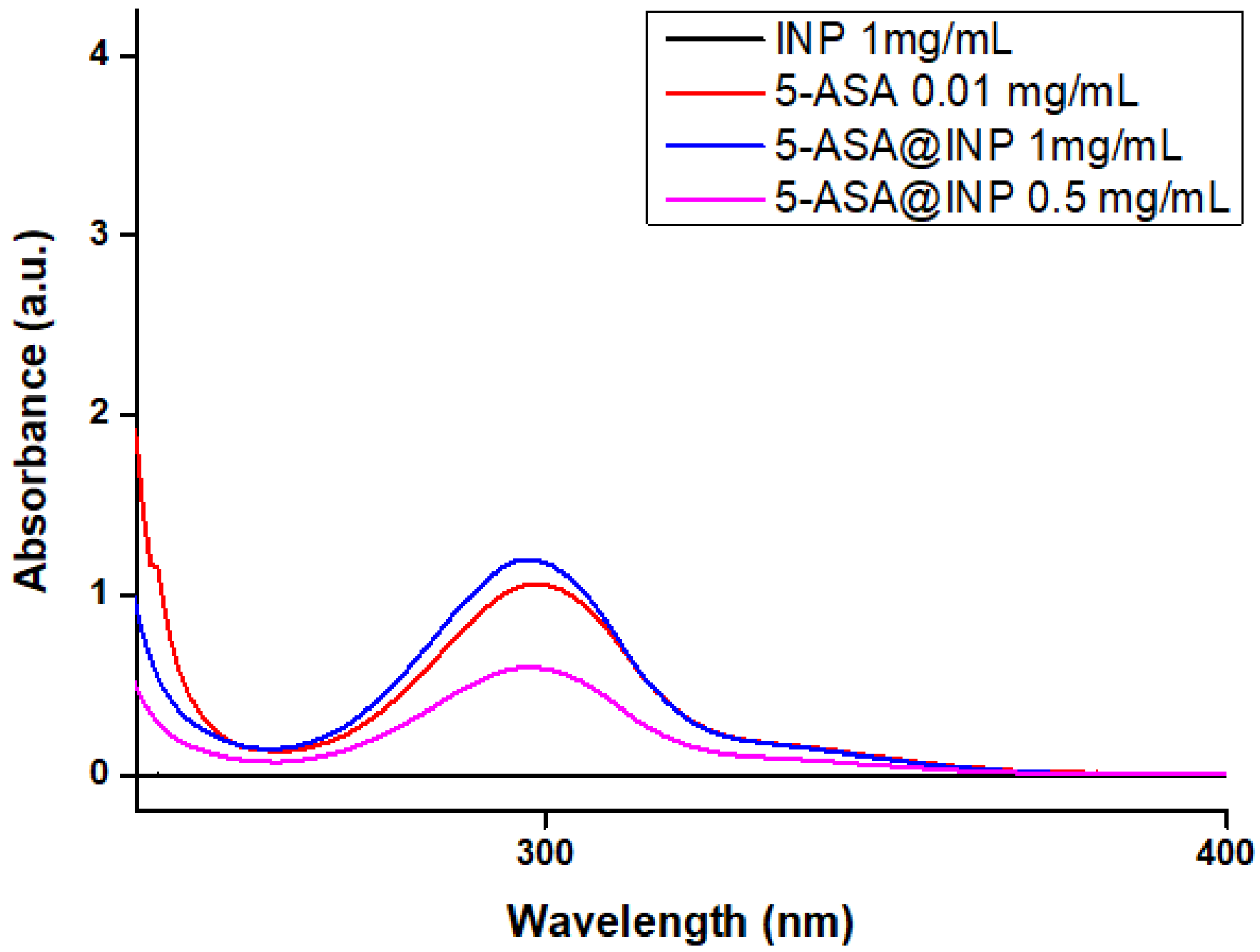

Figure S5. **UV-VIS absorbance spectra of INP and 5-ASA@INP.** The absorbance spectra were measured for INP and 5-ASA@INP at concentrations of 0.5 and 1.0 mg/mL. 5-ASA@INP exhibits a distinct absorption peak at approximately 300 nm. INP shows no specific absorption peak within the 200-400 nm wavelength range.

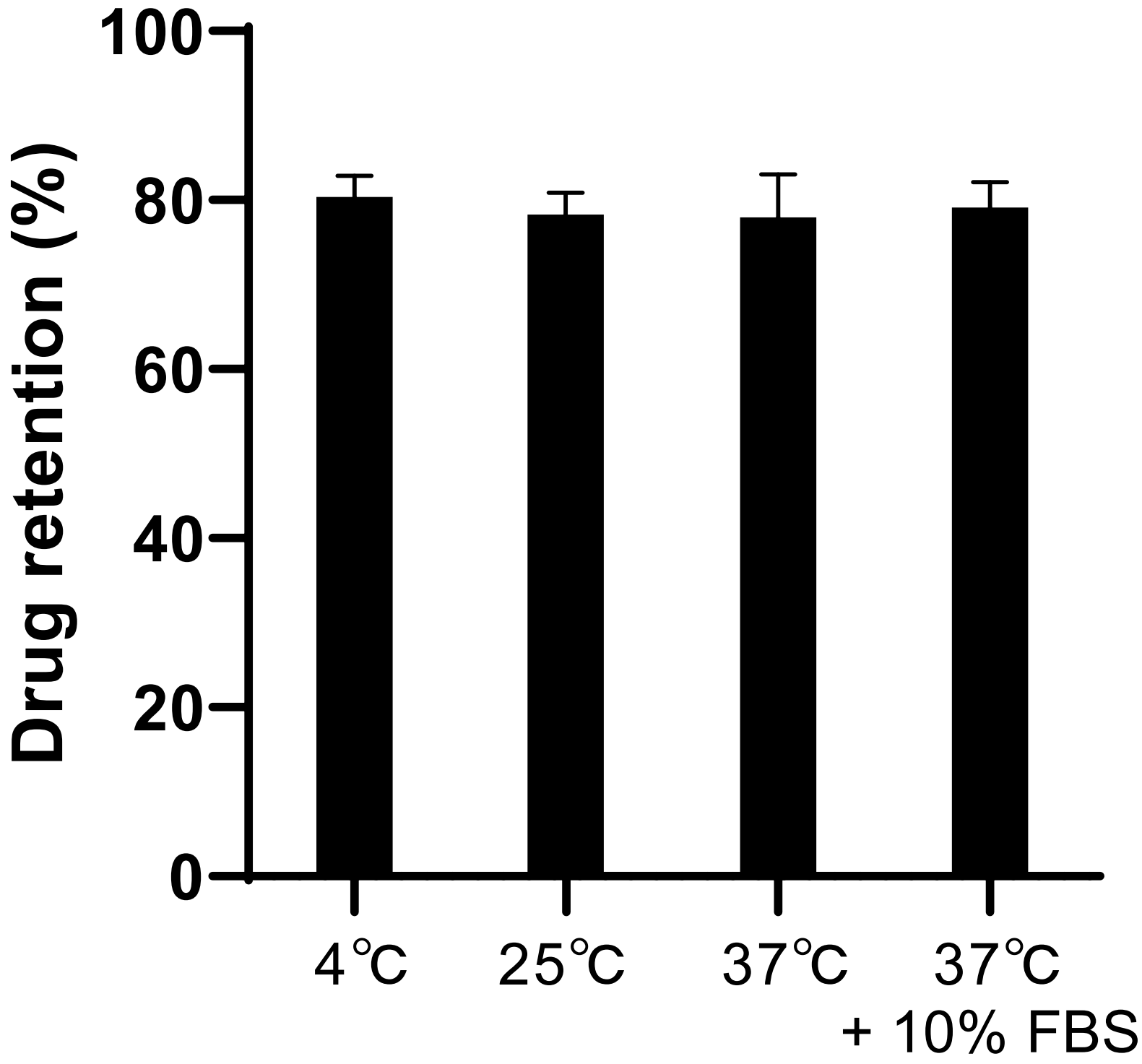

Figure S6. Drug retention of 5-ASA@INP under different storage conditions (4 °C, 25 °C, 37 °C, and 37 °C + 10% FBS). The retained drug content remained at 80.36%, 78.26%, 79.13%, and 77.94%, respectively. Data are presented as mean ± SD (n = 3).

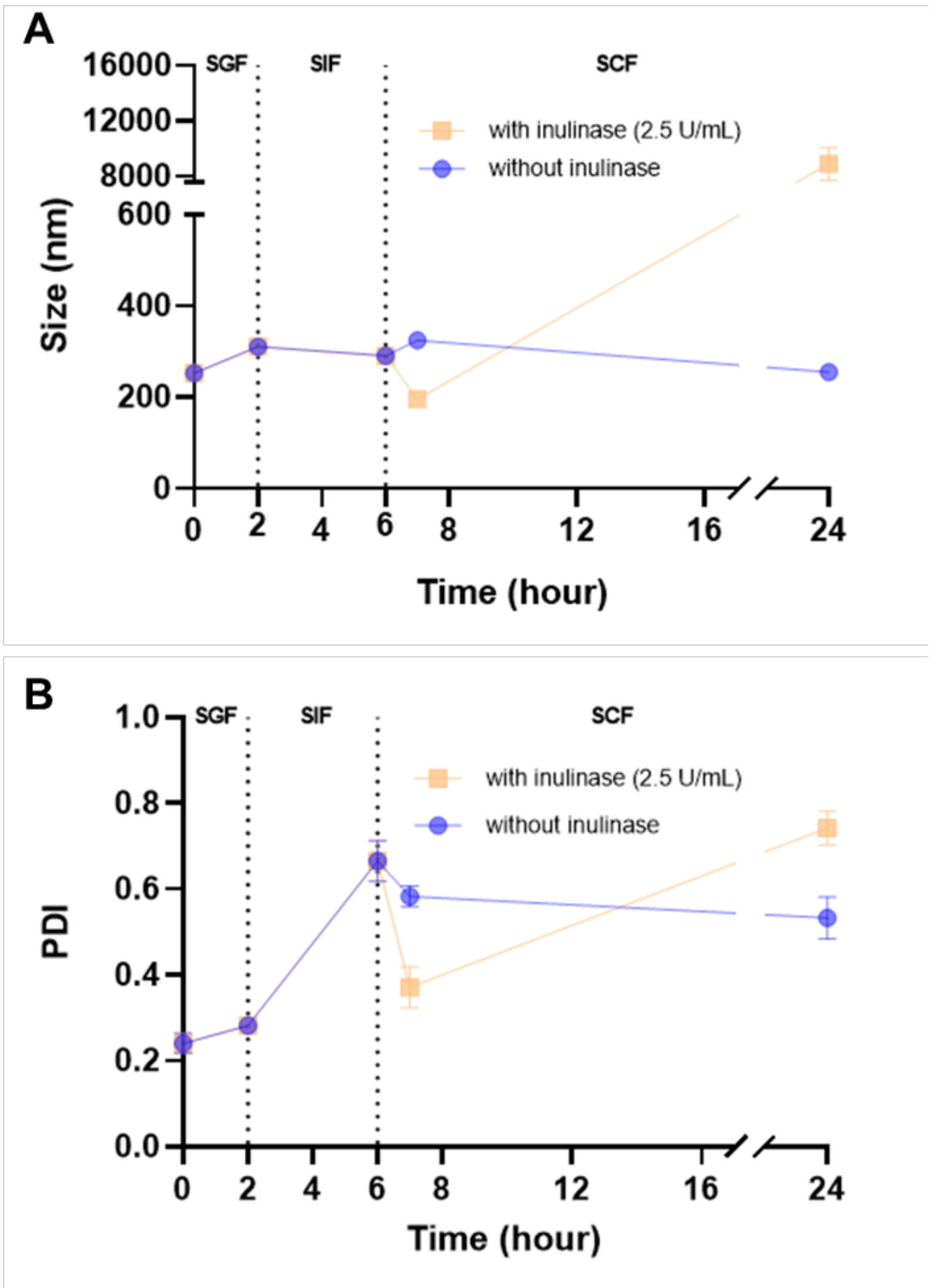

Figure S7. **Physicochemical stability and enzyme-responsive behavior of 5-ASA@INP during sequential simulated gastrointestinal incubation.** (A) Changes in the hydrodynamic diameter (size) and (B) polydispersity index (PDI) of 5-ASA@INP. The nanoparticles were incubated sequentially in simulated gastric fluid (SGF, pH 1.2, 2 h), simulated intestinal fluid (SIF, pH 6.8, 4 h), and simulated colonic fluid (SCF, pH 7.4, 18 h) to mimic the gastrointestinal transit. SCF incubation was performed either in the presence (+ inulinase) or absence (- inulinase) of the enzyme. Particle size maintained within the nanoscale range during the SGF and SIF stages, whereas incubation in SCF containing inulinase resulted in a marked increase in the apparent particle size, indicating enzyme-triggered destabilization of the inulin matrix. In contrast, particles incubated in enzyme-free SCF remained relatively stable. Data are presented as mean ± SD (n = 3).

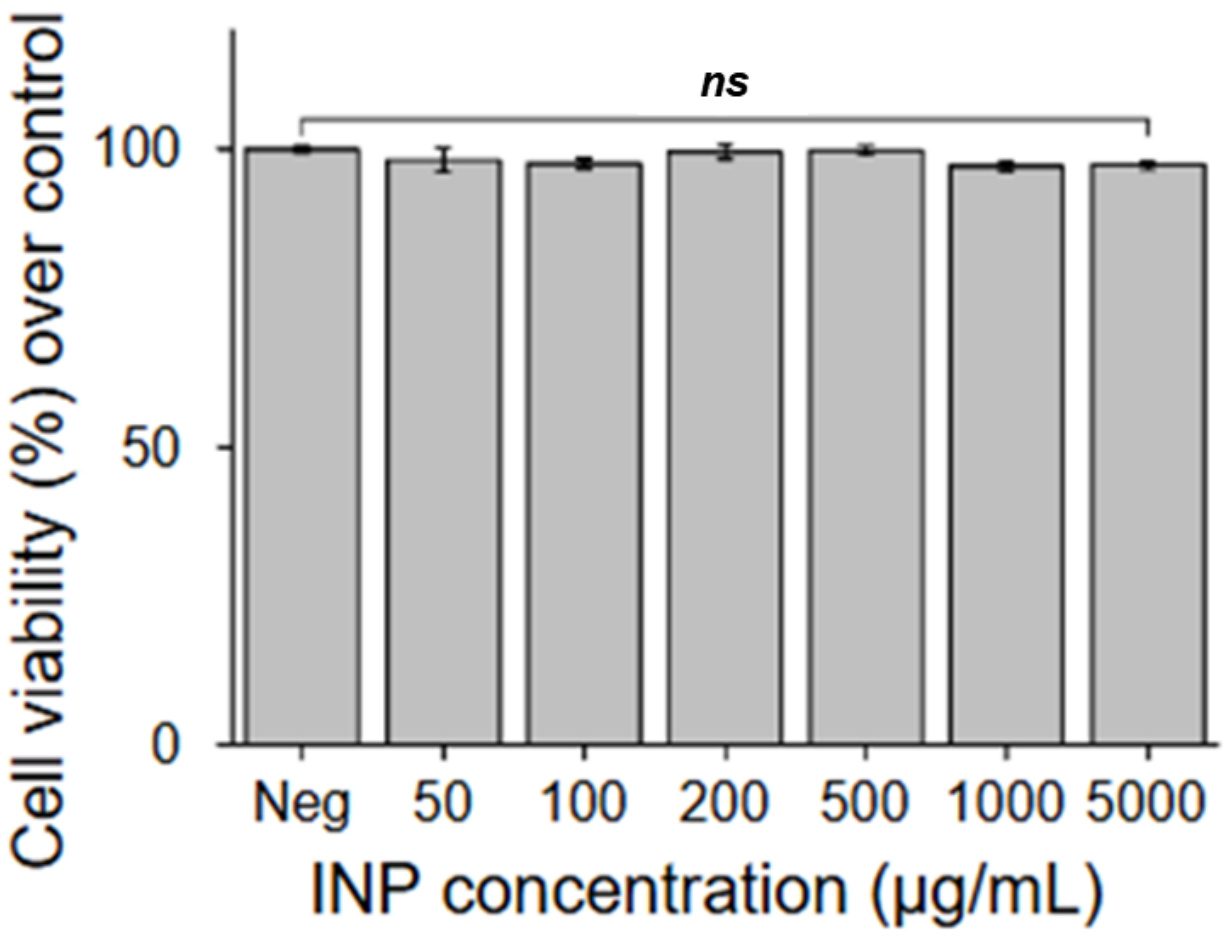

Figure S8. **Cytotoxicity of INP in NIH/3T3.** Cell viability was evaluated after 24 h incubation with various concentrations of INP (50-5,000 µg/mL) using the CCK-8 assay. The "Neg" group represents the untreated control (0 µg/mL INP). Data are presented as mean ± standard deviation (SD, n = 3). Statistical significance between the Neg group and the highest INP concentration group (5,000 µg/mL) was analyzed using Student's *t*-test. *ns*, not significant difference ($p > 0.05$).

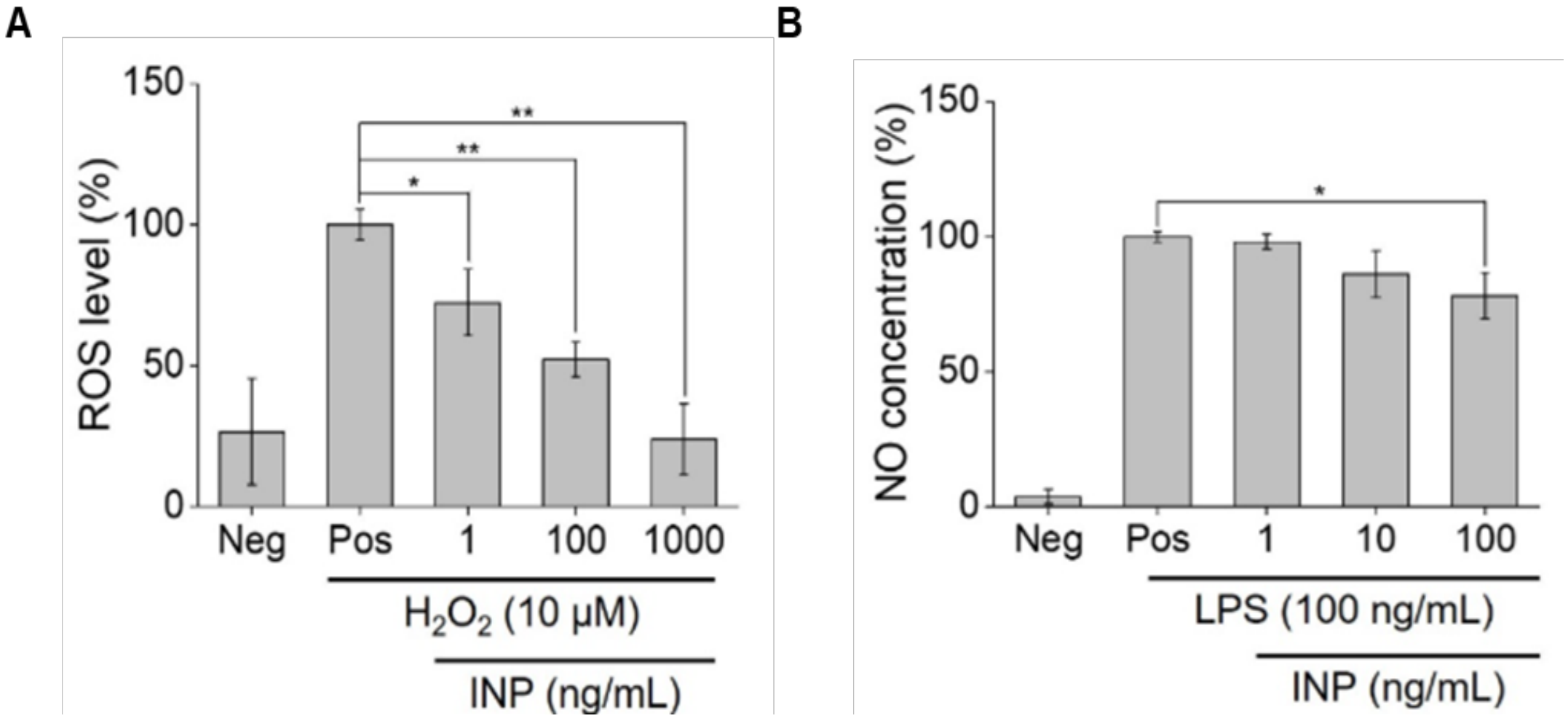

Figure S9. **Antioxidant and anti-inflammatory evaluation of INP in NIH/3T3 and RAW 264.7 cells.** (A) ROS scavenging activity was assessed in H_2O_2-stimulated NIH/3T3 cells treated with different concentrations of INP (1-1,000 ng/mL). (B) NO production was measured in LPS-stimulated RAW 264.7 cells after treatment with INP (1-100 ng/mL). Data are presented as mean ± standard deviation (SD, n = 3). Statistical significance was analyzed using Student's *t*-test. "Neg" and "Pos" represent untreated and stimulated control groups, respectively. *$p < 0.05$ and **$p < 0.01$ indicate statistically significant differences compared with the positive control.

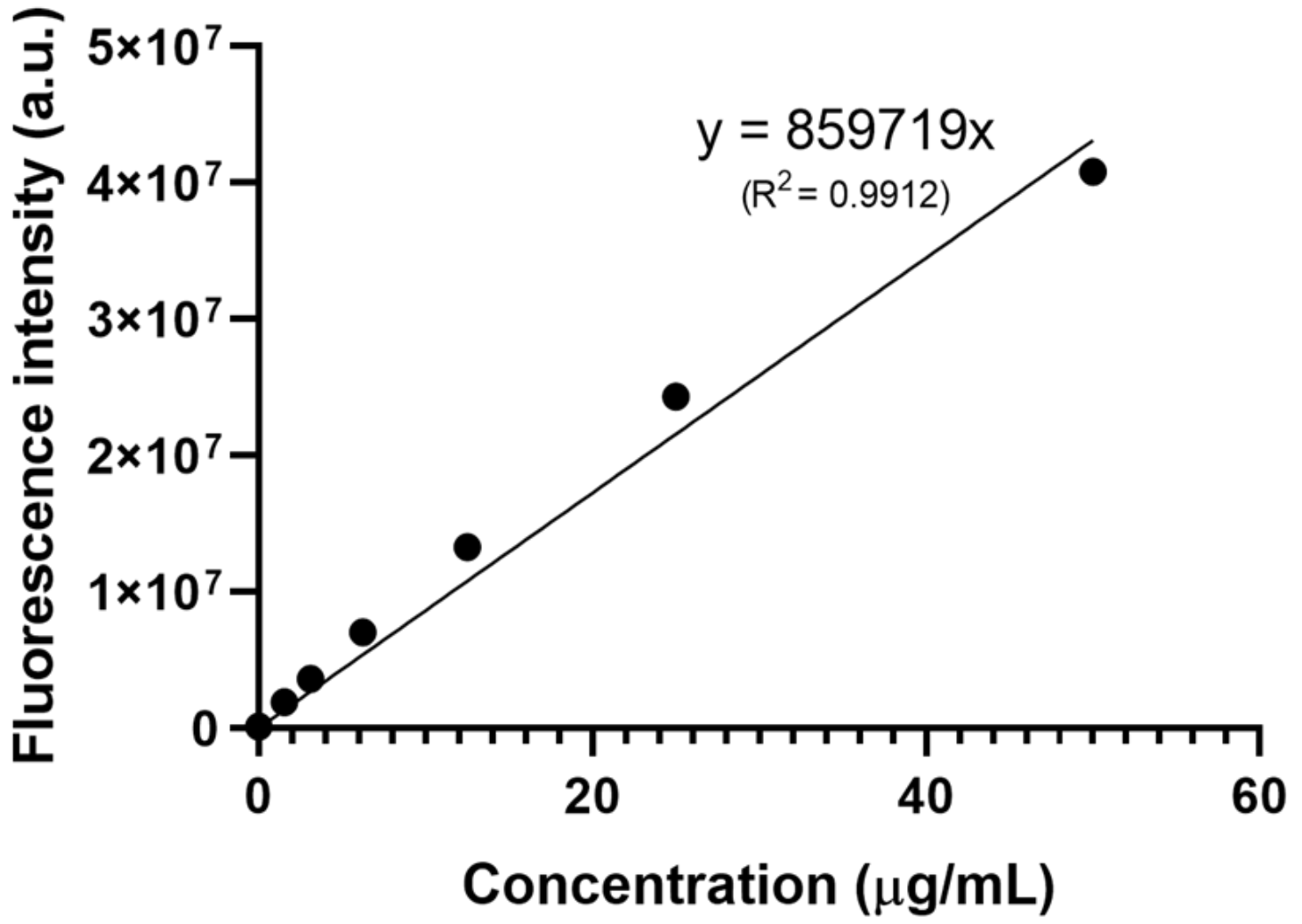

Figure S10. **Standard fluorescence calibration curve of 5-ASA.** The fluorescence intensity of 5-ASA was measured in PBS (pH 7.4) to evaluate its optical behavior in a physiological-like environment. The curve demonstrates a linear relationship between 5-ASA concentration and fluorescence intensity ($R^2 = 0.9912$, y = 859719x) within the tested range. Data are presented as mean ± SD (n = 3).

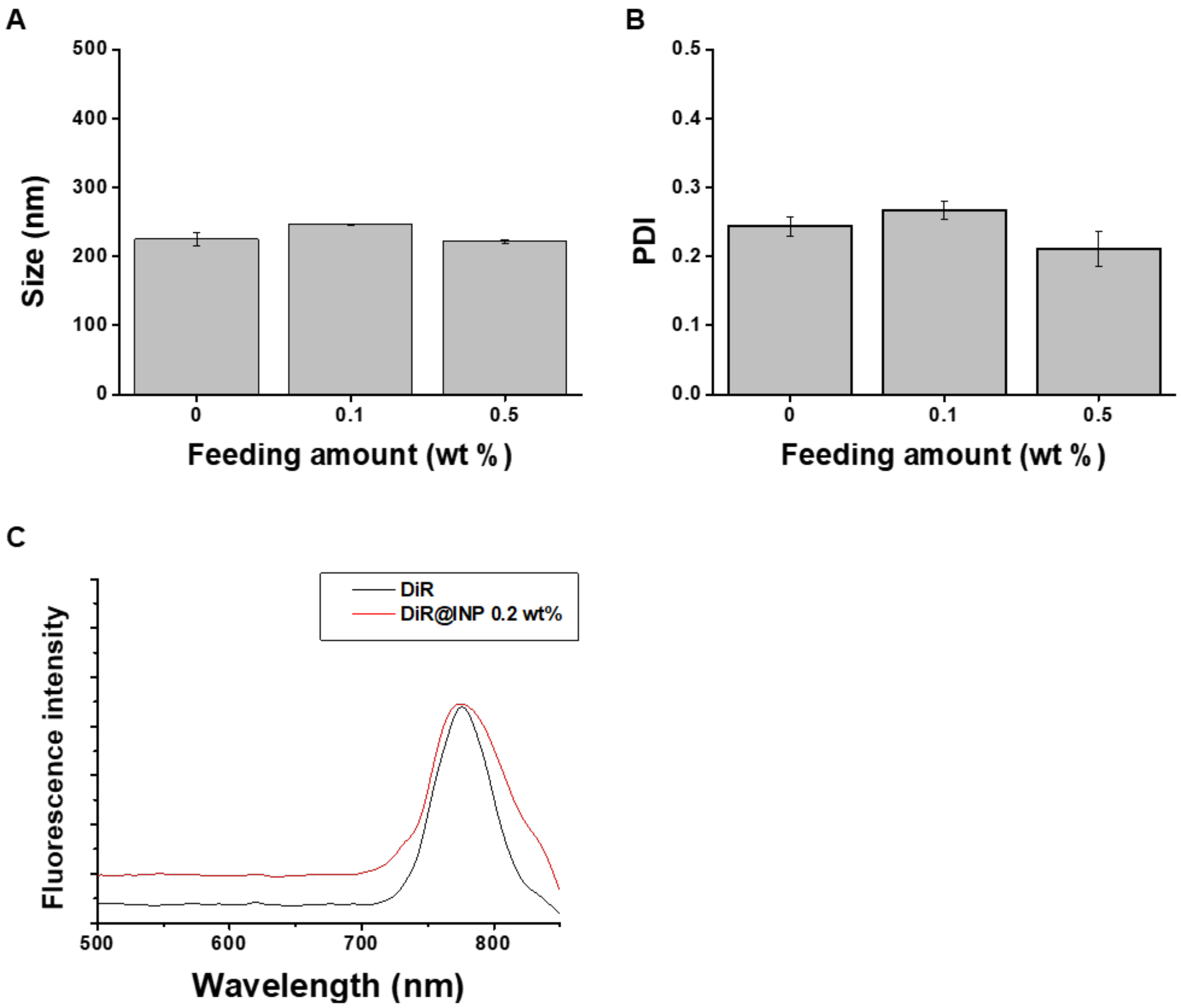

Figure S11. **Physicochemical characterization of DiR@INP.** (A) Particle size and (B) PDI of DiR@INP prepared with different DiR feeding amounts (0-0.5 wt%). (C) Fluorescence spectra of free DiR and DiR@INP (0.2 wt%) showing comparable fluorescence emission profiles. Data are presented as mean ± standard deviation (SD, n = 3).

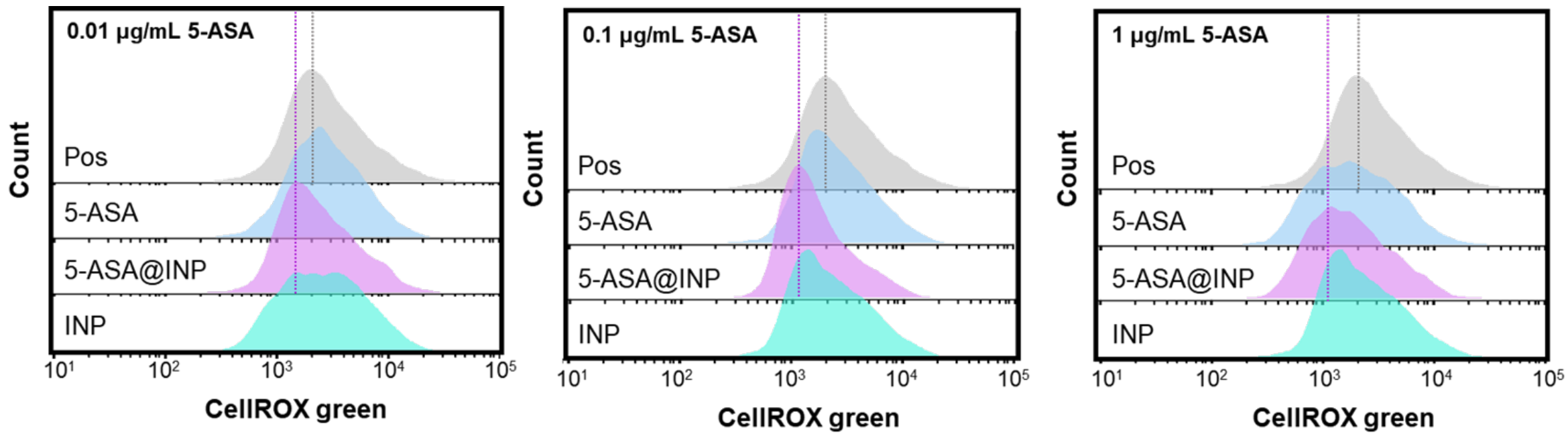

Figure S12. **Flow cytometry analysis of intracellular ROS levels in Caco-2 cells treated with free 5-ASA, 5-ASA@INP, and INP.** Representative flow cytometry histograms showing CellROX^{TM} green fluorescence intensity in Caco-2 cells under H_2O_2-induced oxidative stress conditions. Caco-2 cells were treated with free 5-ASA (0.01-1 μg/mL), 5-ASA@INP (0.01-1 μg/mL, equivalent to 5-ASA), or INP alone (0.1-10 μg/mL).

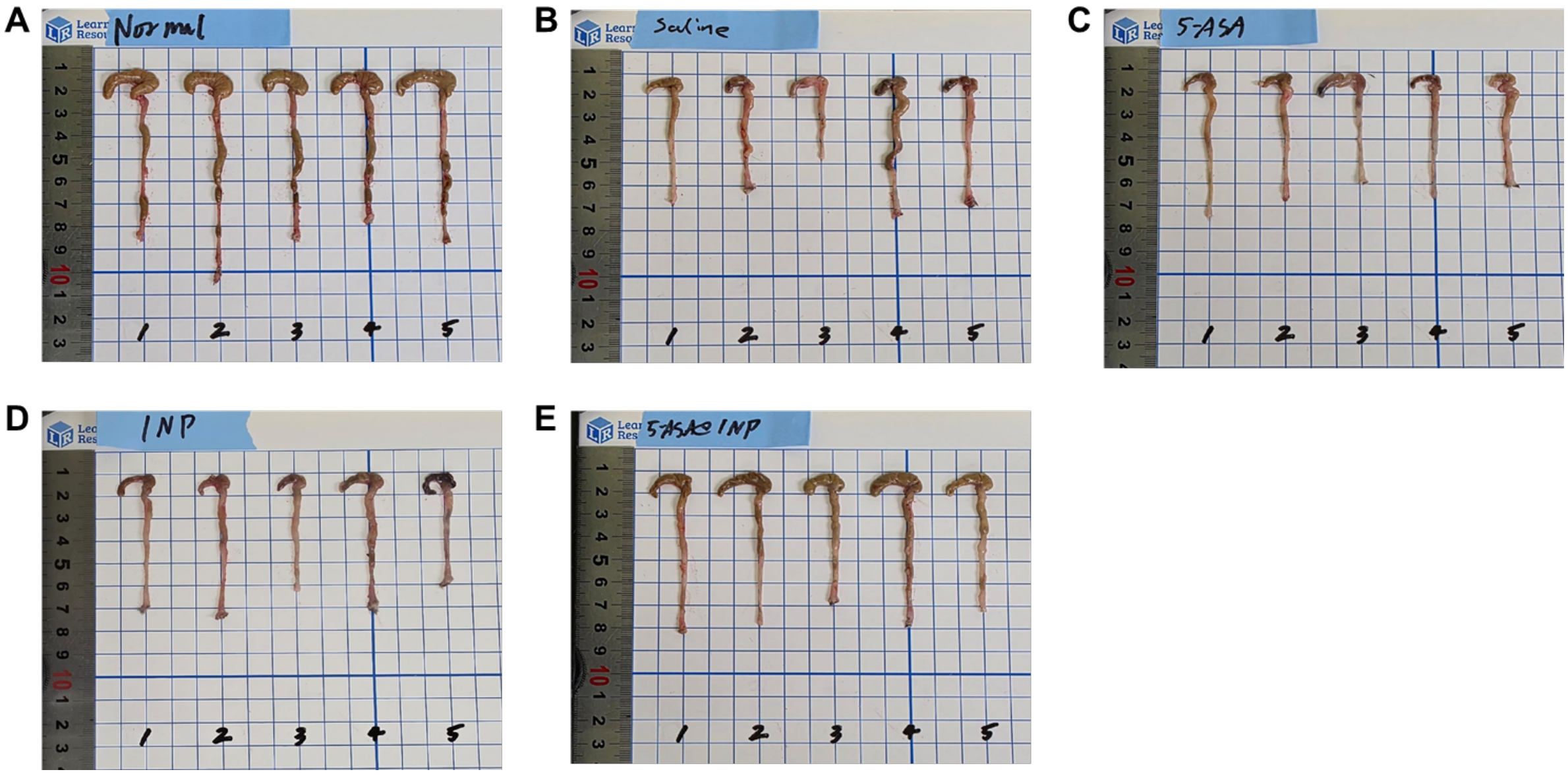

Figure S13. Complete image set of colons from all experimental groups in the DSS-induced colitis mouse model. Gross images of colons collected from all individual mice (n = 5 per group) are shown. Groups include (A) Normal, (B) Saline, (C) 5-ASA, (D) INP, and (E) 5-ASA@INP. Colon length was measured at sacrifice and used for quantitative analysis in Figure 6E.

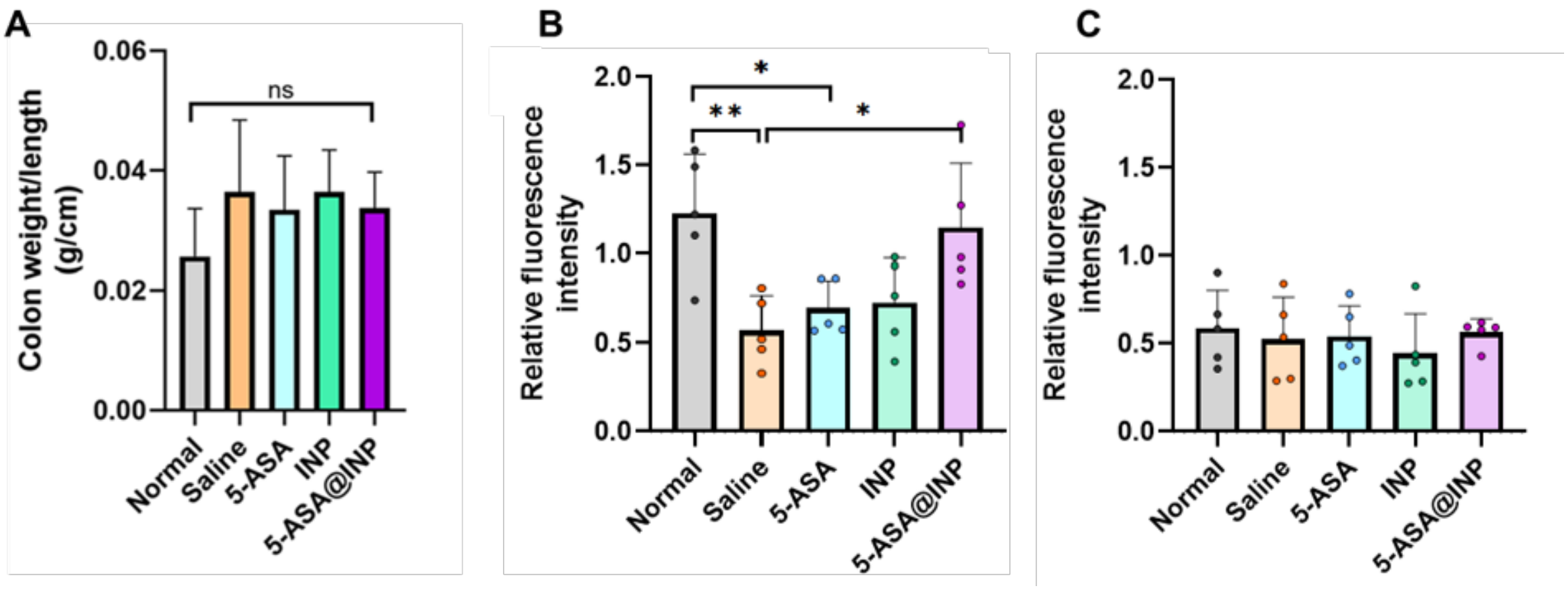

Figure S14. Colon weight-to-length ratio and tight junction protein expression in DSS-induced colitis. (A) Colon weight-to-length ratio (g/cm) measured at sacrifice. (B-C) Quantification of tight junction proteins occludin (B) and ZO-1 (C) in colon tissues. Fluorescence intensity was calculated from immunofluorescence images using ImageJ. Data are presented as mean ± standard deviation (SD, n = 5 per group). Statistical significance was determined using one-way ANOVA, followed by Tukey's post hoc multiple comparison tests. *ns*, not significant; *$p < 0.05$, **$p < 0.01$, ***$p < 0.001$.

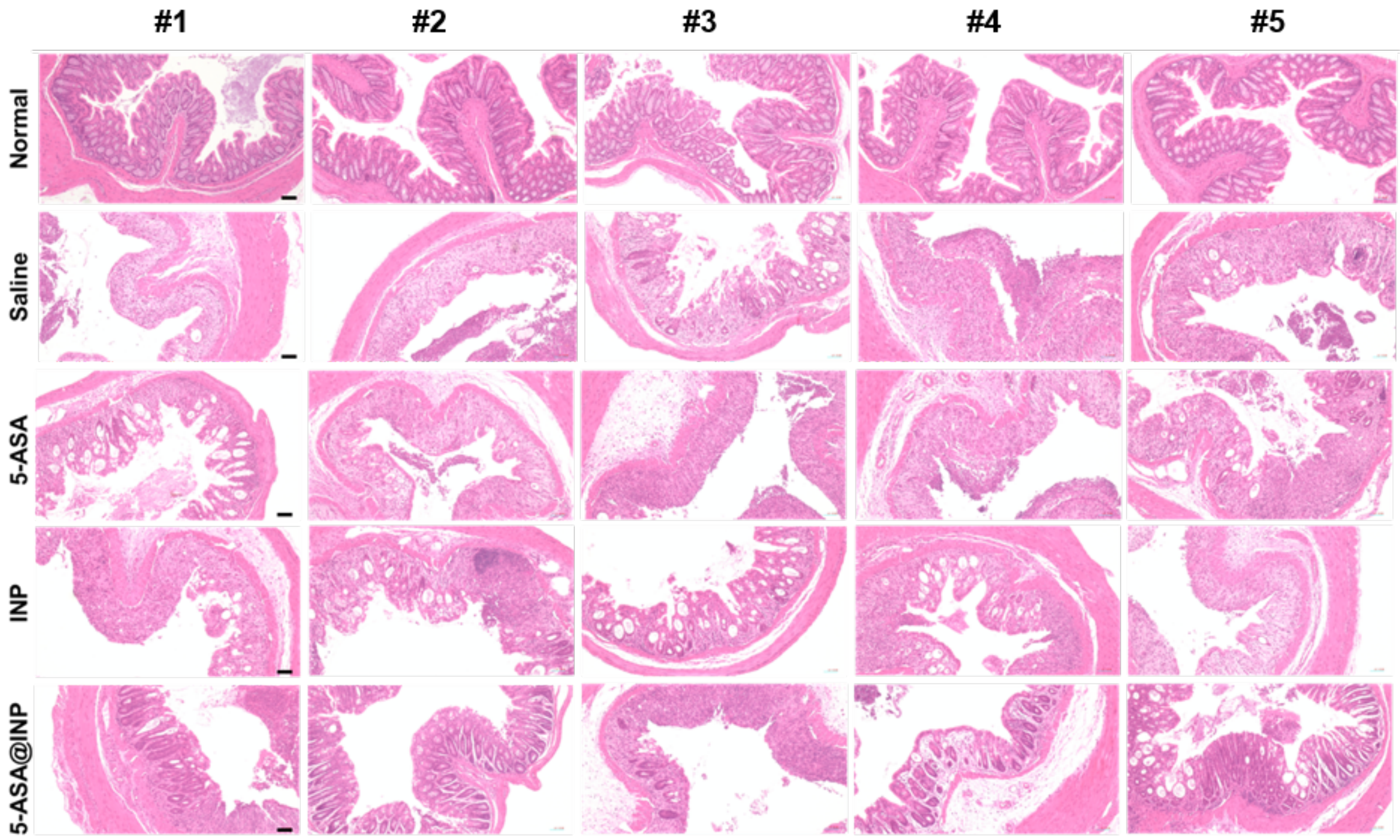

Figure S15. Complete set of H&E-stained colon sections from all experimental groups in the DSS-induced colitis mouse model. Each row represents a different experimental group (Row 1: Normal; Row 2: Saline; Row 3: 5-ASA; Row 4: INP; Row 5: 5-ASA@INP), and each column (#1-#5) corresponds to individual mice within the same group (n = 5 per group). Scale bars = 60 μm; Magnification, ×100.

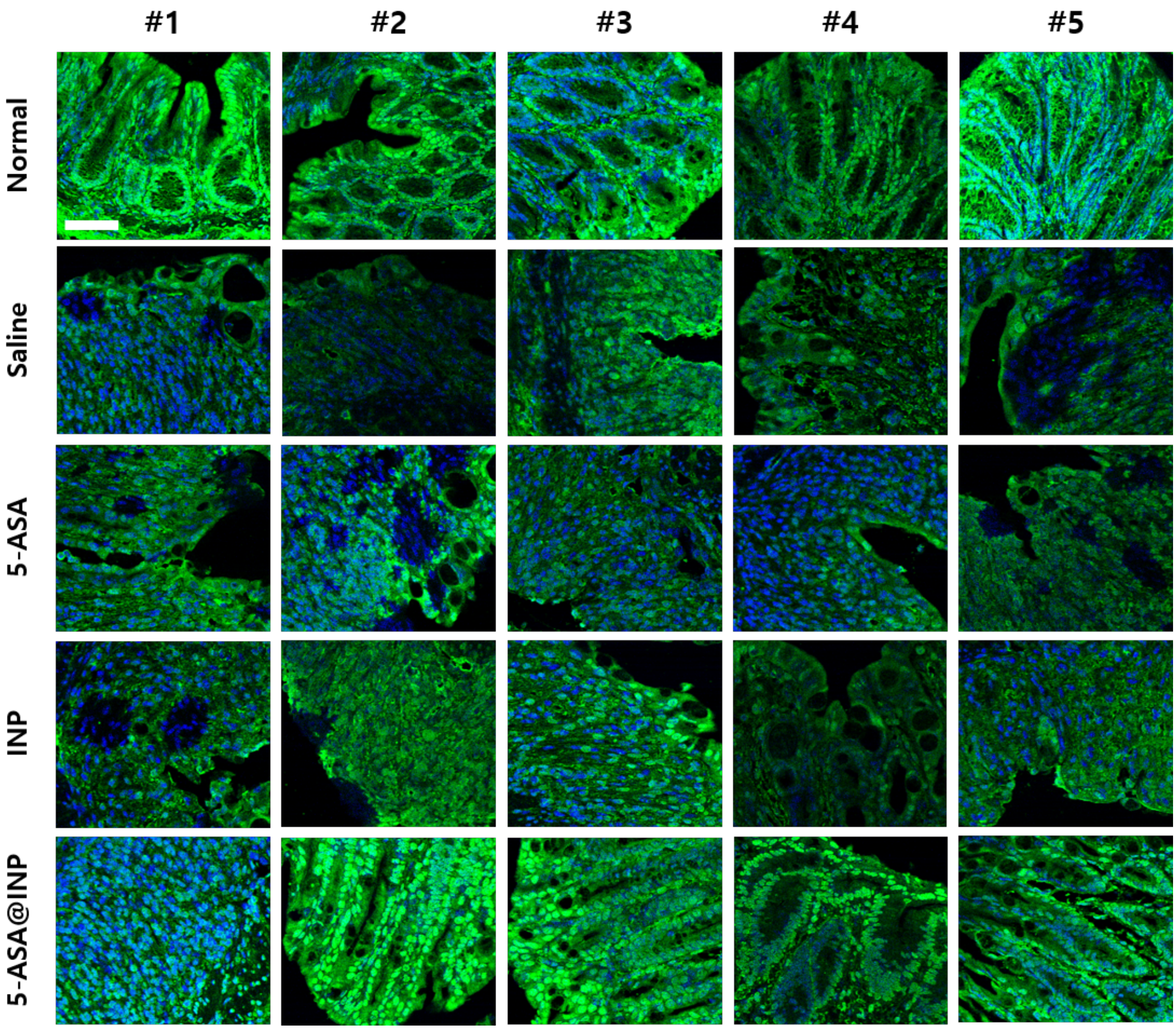

Figure S16. Complete set of immunofluorescence images of the tight junction protein Occludin in colon sections from all experimental groups in the DSS-induced colitis mouse model. Immunofluorescence staining of Occludin (green) with nuclear counterstaining (DAPI, blue) is shown for all individual mice. Each row represents a different experimental group (Row 1: Normal; Row 2: Saline; Row 3: 5-ASA; Row 4: INP; Row 5: 5-ASA@INP), and each column (#1-#5) corresponds to individual mice within the same group (n = 5 per group). Scale bars = 50 μm; Magnification, ×200.

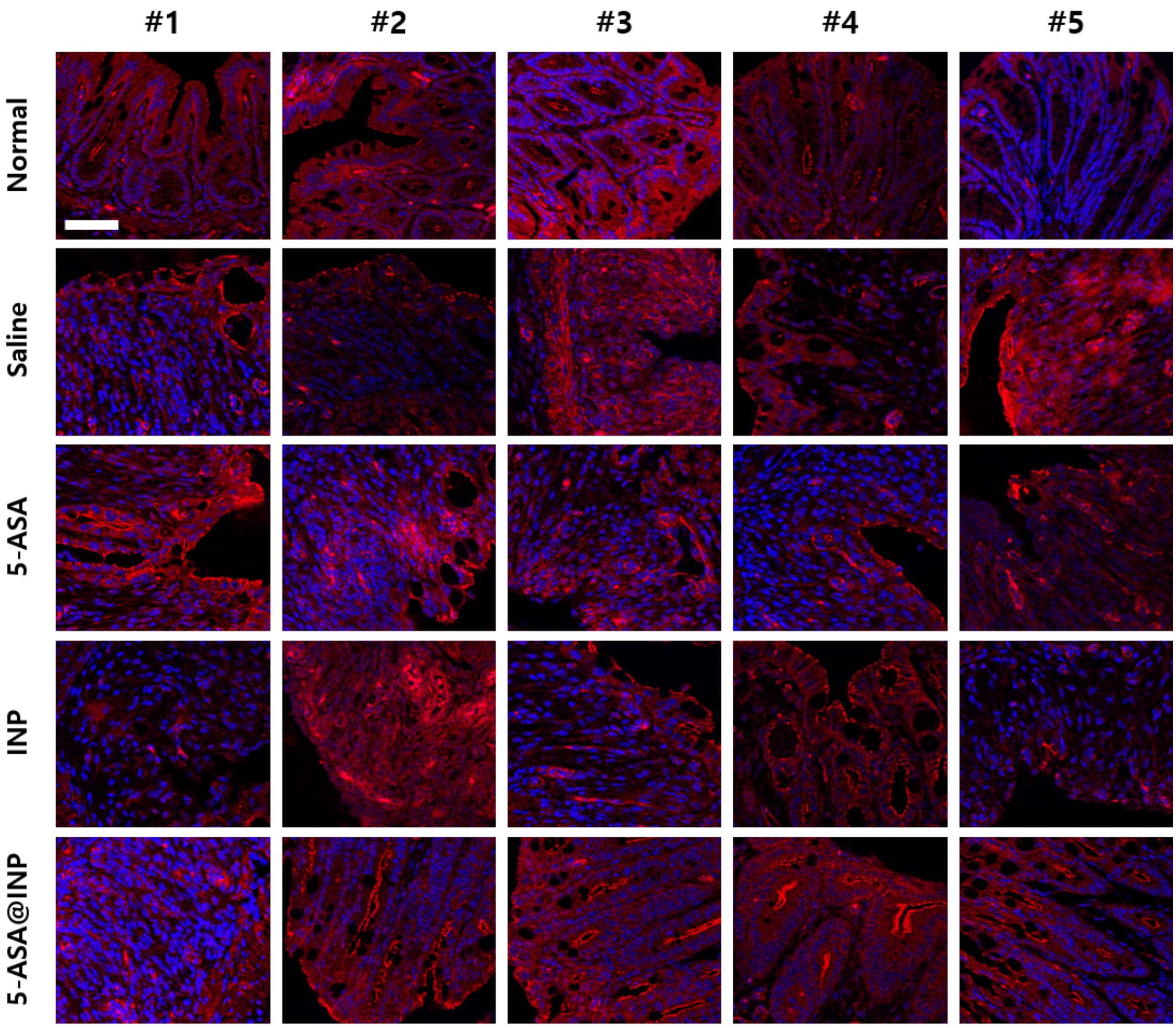

Figure S17. **Complete set of immunofluorescence images of the tight junction protein ZO-1 in colon sections from all experimental groups in the DSS-induced colitis mouse model.** Immunofluorescence staining of ZO-1 (red) with nuclear counterstaining (DAPI, blue) is shown for all individual mice. Each row represents a different experimental group (Row 1: Normal; Row 2: Saline; Row 3: 5-ASA; Row 4: INP; Row 5: 5-ASA@INP), and each column (#1-#5) corresponds to individual mice within the same group (n = 5 per group). Scale bars = 50 μm; Magnification, ×200.

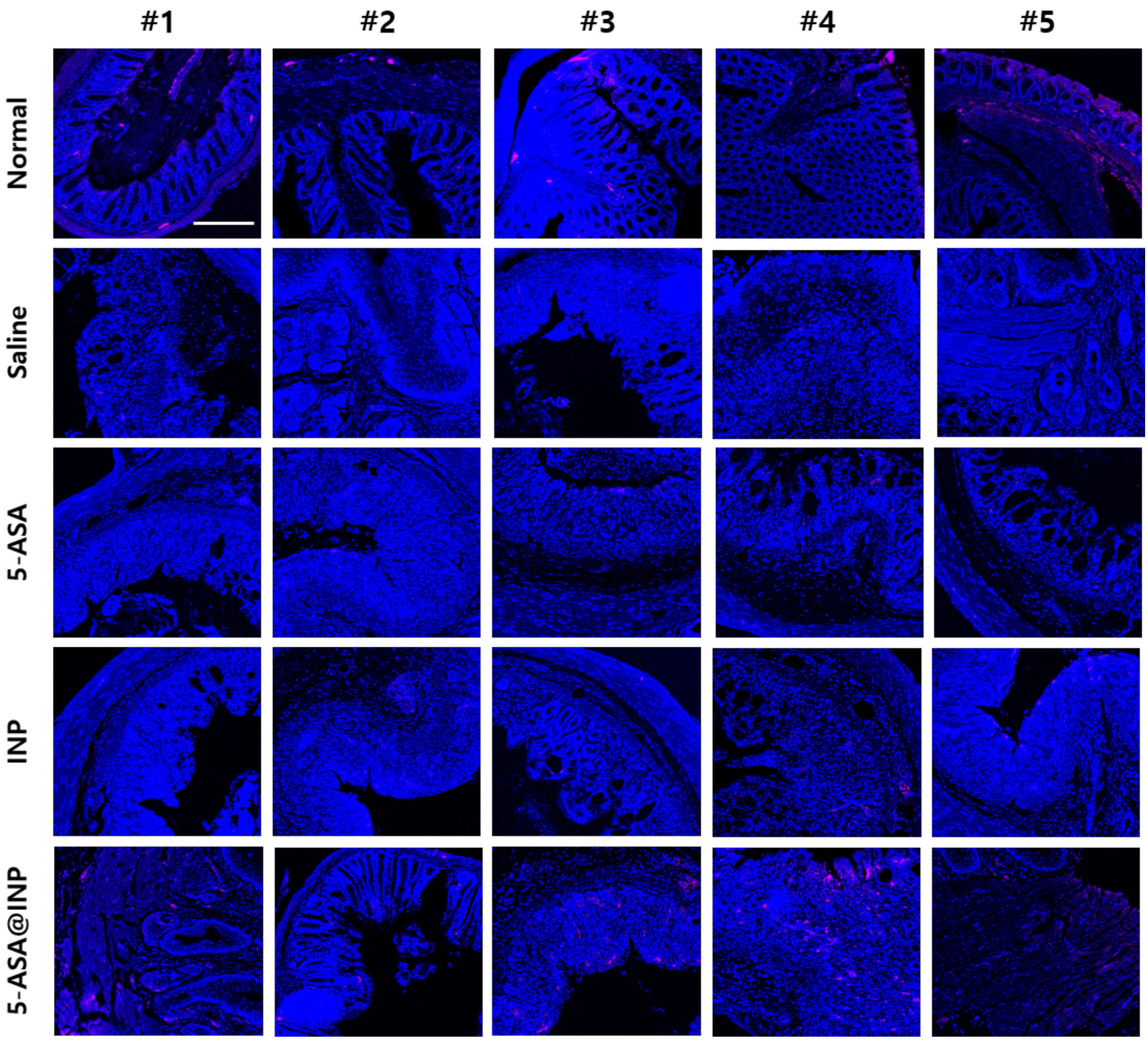

Figure S18. **Complete set of FOXP3 immunofluorescence images in colon tissues from all experimental groups in the DSS-induced colitis mouse model.** Immunofluorescence staining of FOXP3 (red) with nuclear counterstaining (DAPI, blue) is shown for all individual mice. Each row represents a different experimental group (Row 1: Normal; Row 2: Saline; Row 3: 5-ASA; Row 4: INP; Row 5: 5-ASA@INP), and each column (#1-#5) corresponds to individual mice within the same group (n = 5 per group). Scale bars = 200 μm; Magnification, ×100.

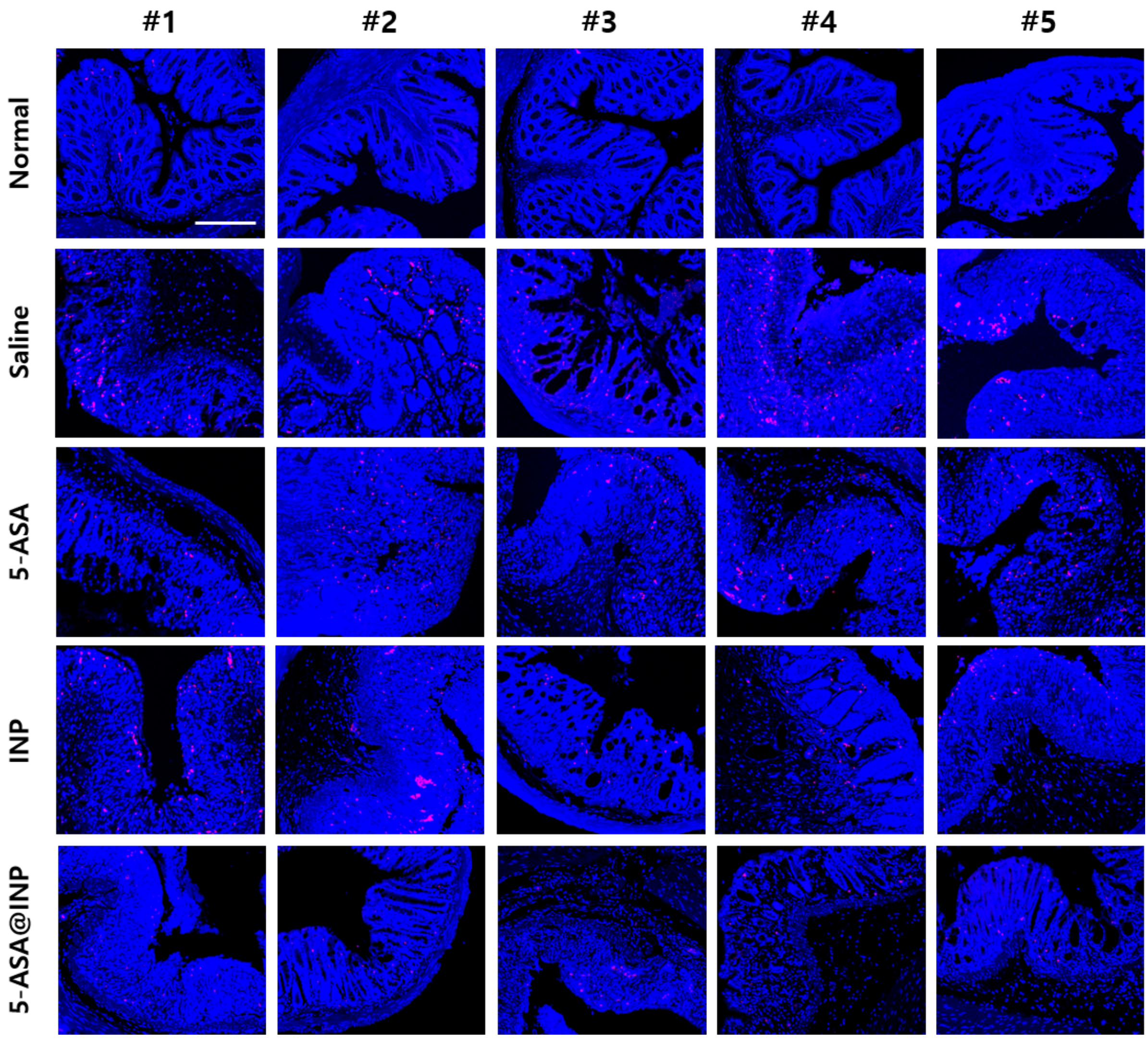

Figure S19. **Complete set of IL-17A immunofluorescence images in colon tissues from all experimental groups in the DSS-induced colitis mouse model.** Immunofluorescence staining of IL-17A (green) with nuclear counterstaining (DAPI, blue) is shown for all individual mice. Each row represents a different experimental group (Row 1: Normal; Row 2: Saline; Row 3: 5-ASA; Row 4: INP; Row 5: 5-ASA@INP), and each column (#1-#5) corresponds to individual mice within the same group (n = 5 per group). Scale bars = 200 μm; Magnification, ×100.

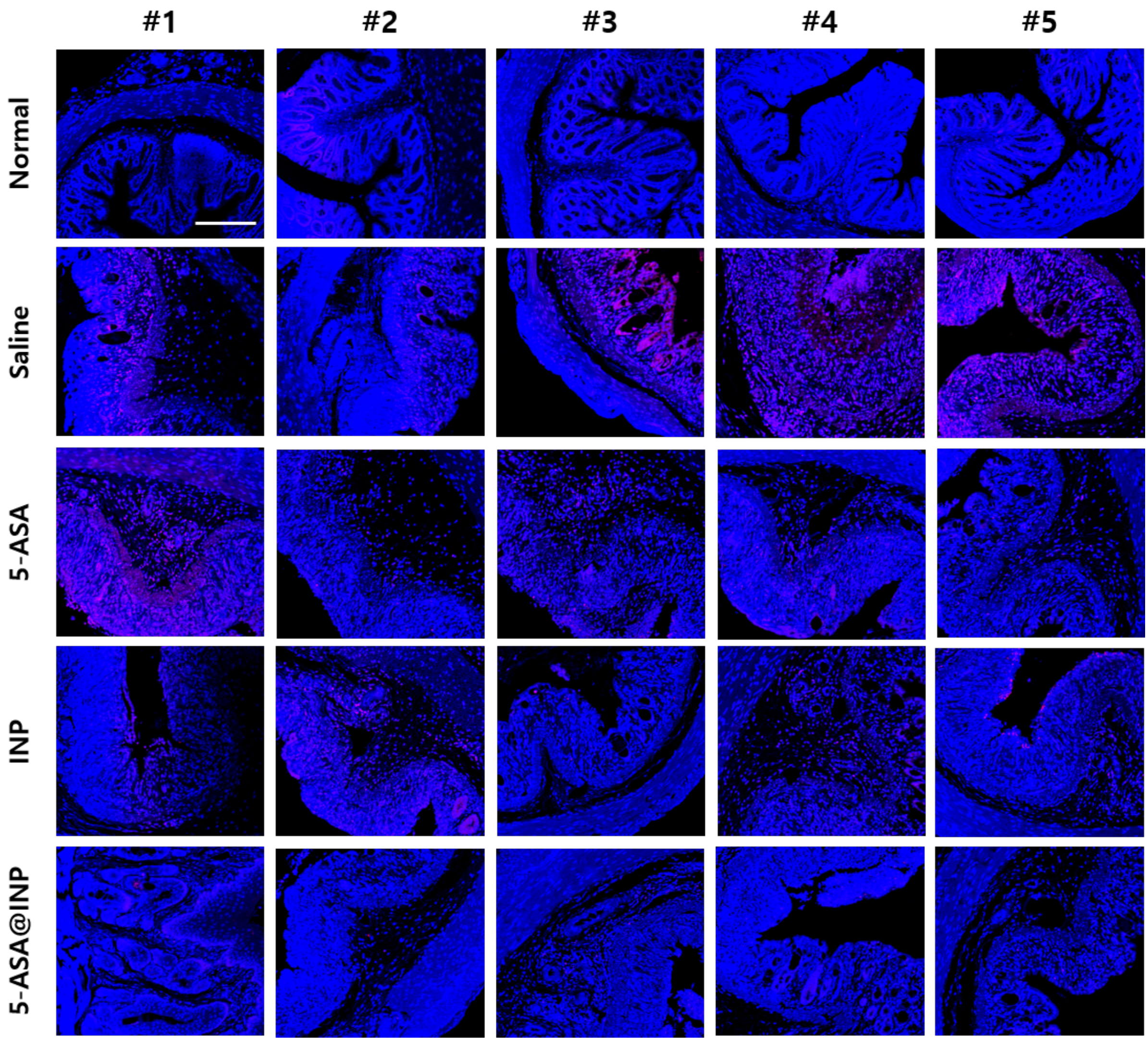

Figure S20. **Complete set of TNF-α immunofluorescence images in colon tissues from all experimental groups in the DSS-induced colitis mouse model.** Immunofluorescence staining of TNF-α (red) with nuclear counterstaining (DAPI, blue) is shown for all individual mice. Each row represents a different experimental group (Row 1: Normal; Row 2: Saline; Row 3: 5-ASA; Row 4: INP; Row 5: 5-ASA@INP), and each column (#1-#5) corresponds to individual mice within the same group (n = 5 per group). Scale bars = 200 μm; Magnification, ×100.

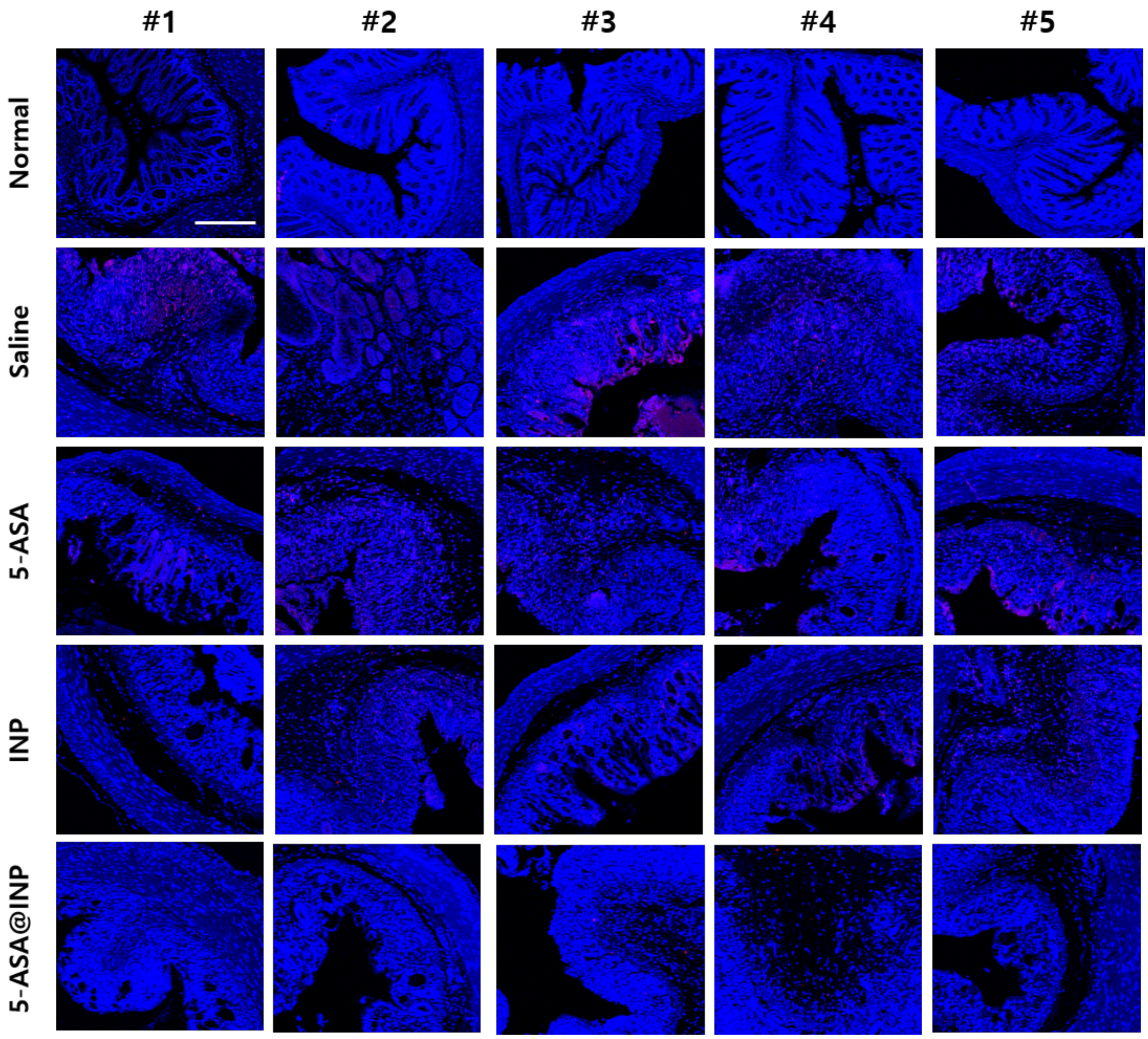

Figure S21. **Complete set of IL-1β immunofluorescence images in colon tissues from all experimental groups in the DSS-induced colitis mouse model.** Immunofluorescence staining of IL-1β (red) with nuclear counterstaining (DAPI, blue) is shown for all individual mice. Each row represents a different experimental group (Row 1: Normal; Row 2: Saline; Row 3: 5-ASA; Row 4: INP; Row 5: 5-ASA@INP), and each column (#1-#5) corresponds to individual mice within the same group (n = 5 per group). Scale bars = 200 μm; Magnification, ×100.

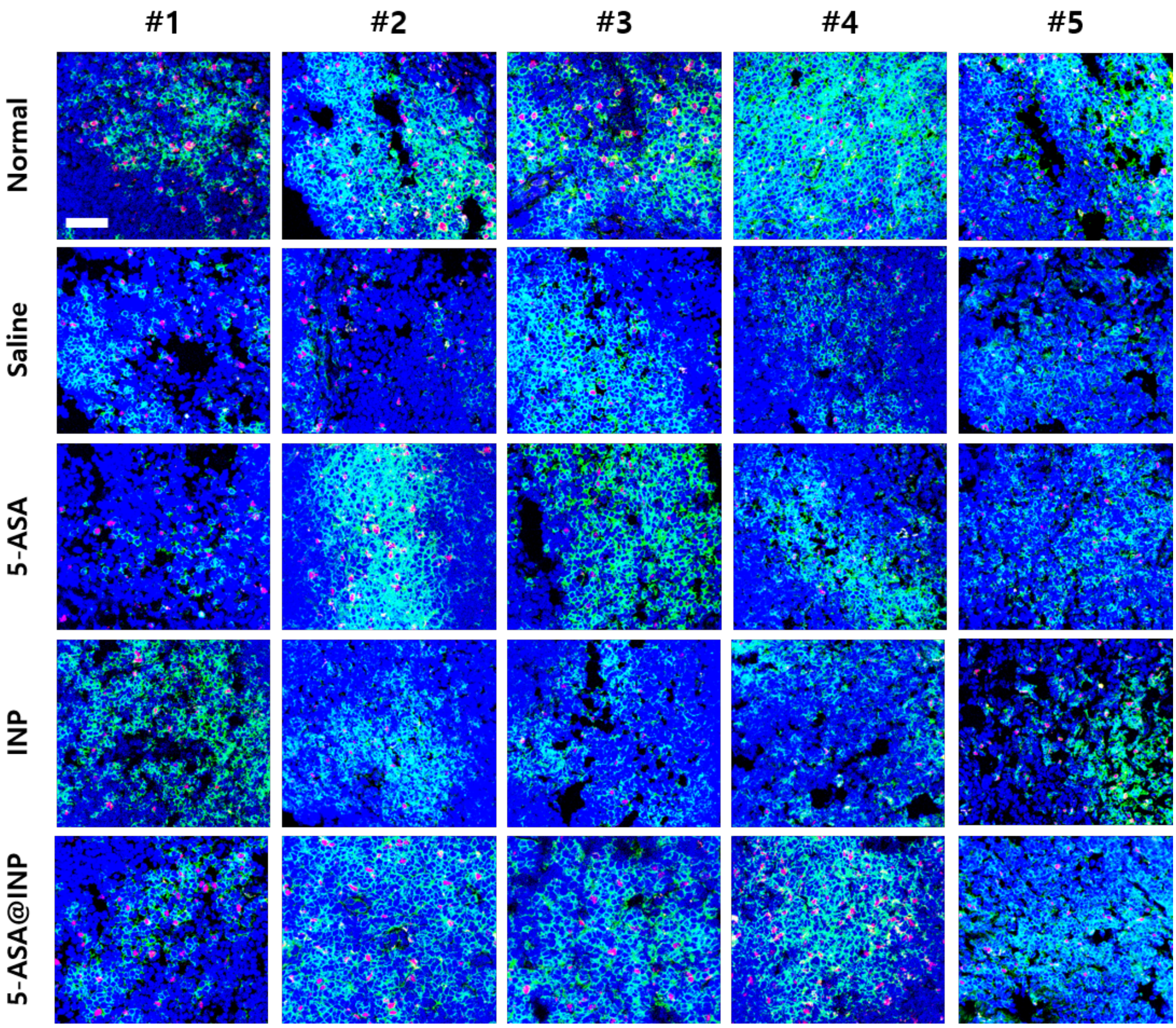

Figure S22. **Complete set of immunofluorescence images of $CD3^{+}FOXP3^{+}$ Treg cells in Peyer's patches (PPs) from DSS-induced colitis.** Immunofluorescence staining of CD3 (green) and FOXP3 (red) with nuclear counterstaining (DAPI, blue) is shown for all individual mice. Each row represents a different experimental group (Row 1: Normal; Row 2: Saline; Row 3: 5-ASA; Row 4: INP; Row 5: 5-ASA@INP), and each column (#1-#5) corresponds to individual mice within the same group (n = 5 per group). Scale bars = 25 μm; Magnification, ×400.

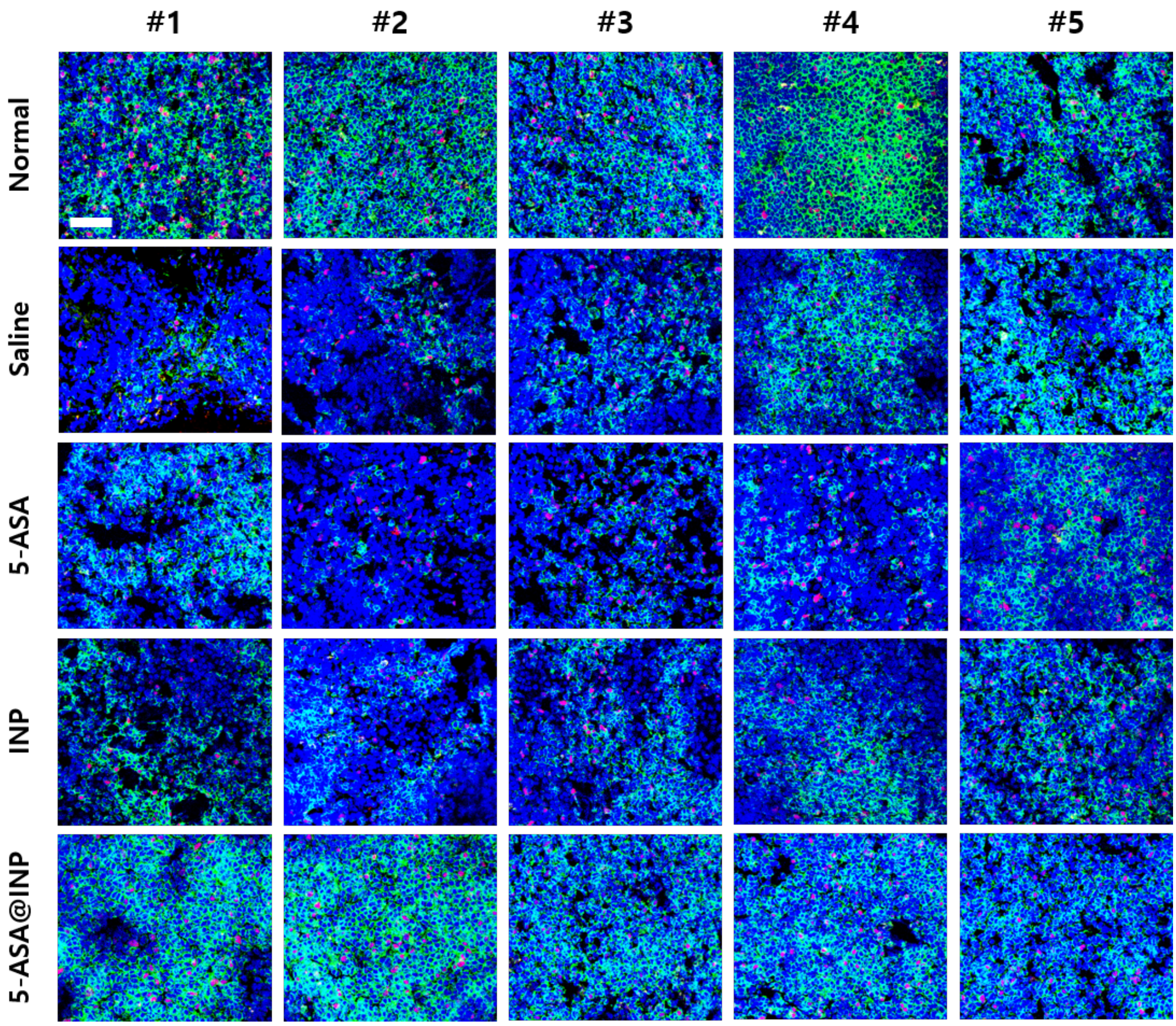

Figure S23. **Complete set of immunofluorescence images of CD3$^+$FOXP3$^+$ Treg cells in mesenteric lymph nodes (MLNs) from DSS-induced colitis.** Immunofluorescence staining of CD3 (green) and FOXP3 (red) with nuclear counterstaining (DAPI, blue) is shown for all individual mice. Each row represents a different experimental group (Row 1: Normal; Row 2: Saline; Row 3: 5-ASA; Row 4: INP; Row 5: 5-ASA@INP), and each column (#1-#5) corresponds to individual mice within the same group (n = 5 per group). Scale bars = 25 μm; Magnification, ×400.

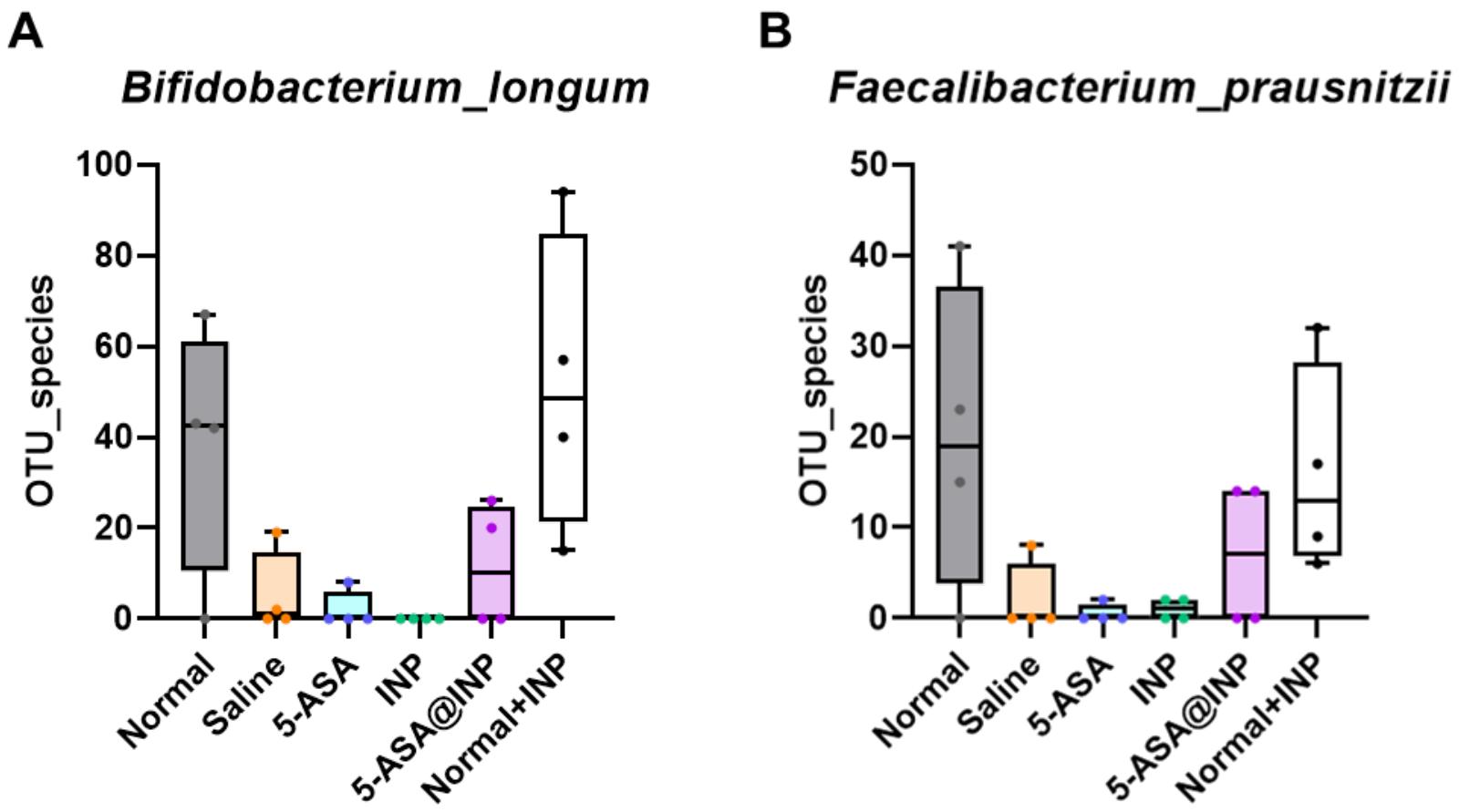

Figure S24. **Taxonomic profiling of key gut microbiota at the species level via 16S rRNA gene sequencing.** Relative abundance of (A) *Bifidobacterium longum* and (B) *Faecalibacterium prausnitzii* expressed as the number of observed OTUs in fecal samples. Data are presented as mean ± standard deviation (SD, n = 4 per group).

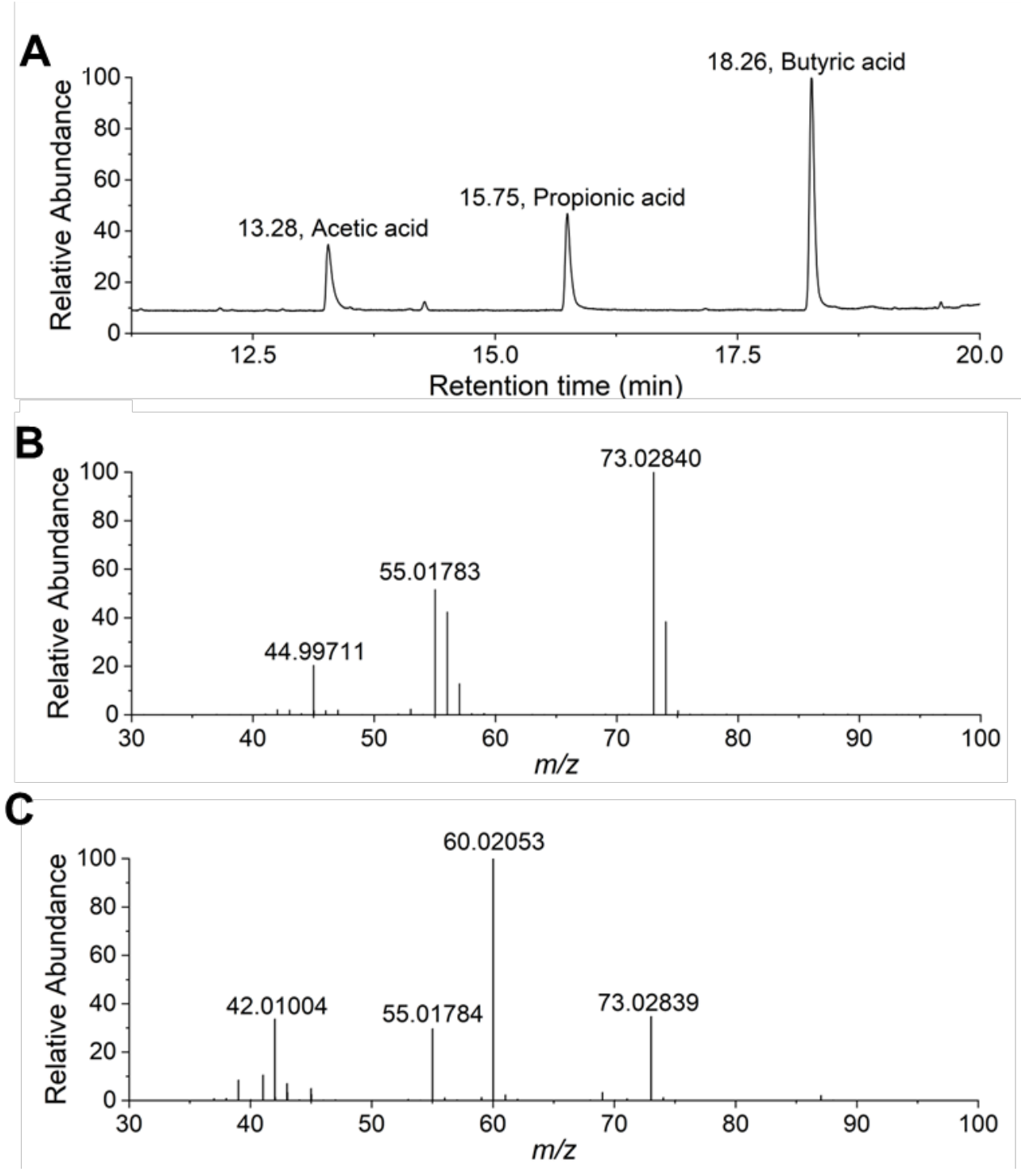

Figure S25. **GC-MS analysis and mass spectral identification of short-chain fatty acids.** (A) Representative gas chromatogram profile showing the separation of acetic acid (13.28 min), propionic acid (15.75 min), and butyric acid (18.26 min). (B, C) Mass spectra of (B) propionic acid and (C) butyric acid, with characteristic fragment ions (m/z) used for structural identification.

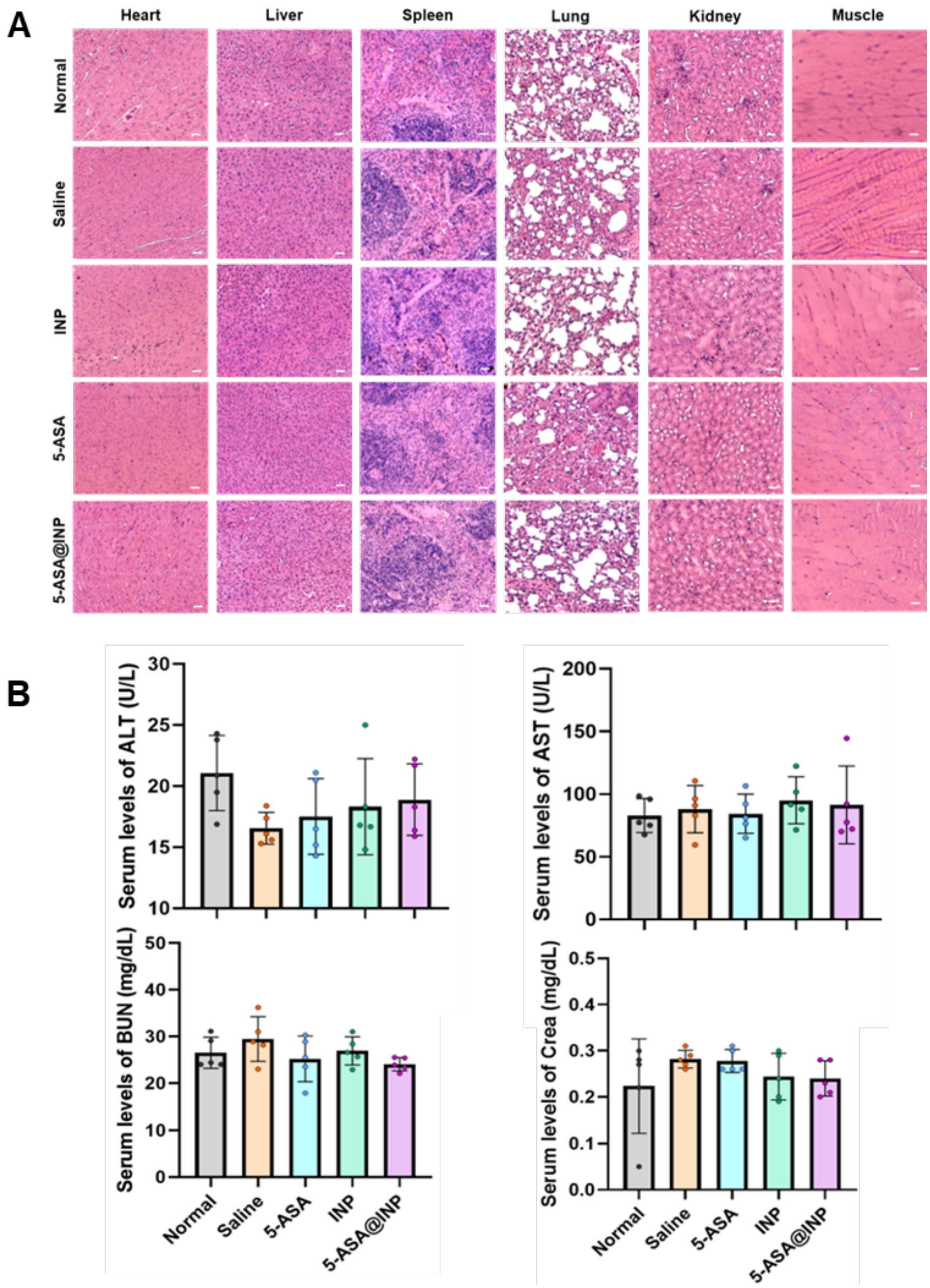

Figure S26. **Histological and biochemical evaluation of systemic safety after oral administration of 5-ASA, INP and 5-ASA@INP in DSS-induced colitis mice**. (A) Representative H&E-stained sections of major organs including heart, liver, spleen, lung, kidney, and muscle. No apparent pathological abnormalities were observed across treatment groups. Scale bars = 100 μm; Magnification, ×200. (B) Serum biochemical parameters related to hepatic and renal function, including alanine aminotransferase (ALT), aspartate aminotransferase (AST), blood urea nitrogen (BUN), and creatinine (Crea). Data are presented as mean ± standard deviation (SD, n = 5 per group). Statistical analysis was performed using one-way ANOVA followed by Tukey's multiple comparison test. No statistically significant differences were observed among the groups.

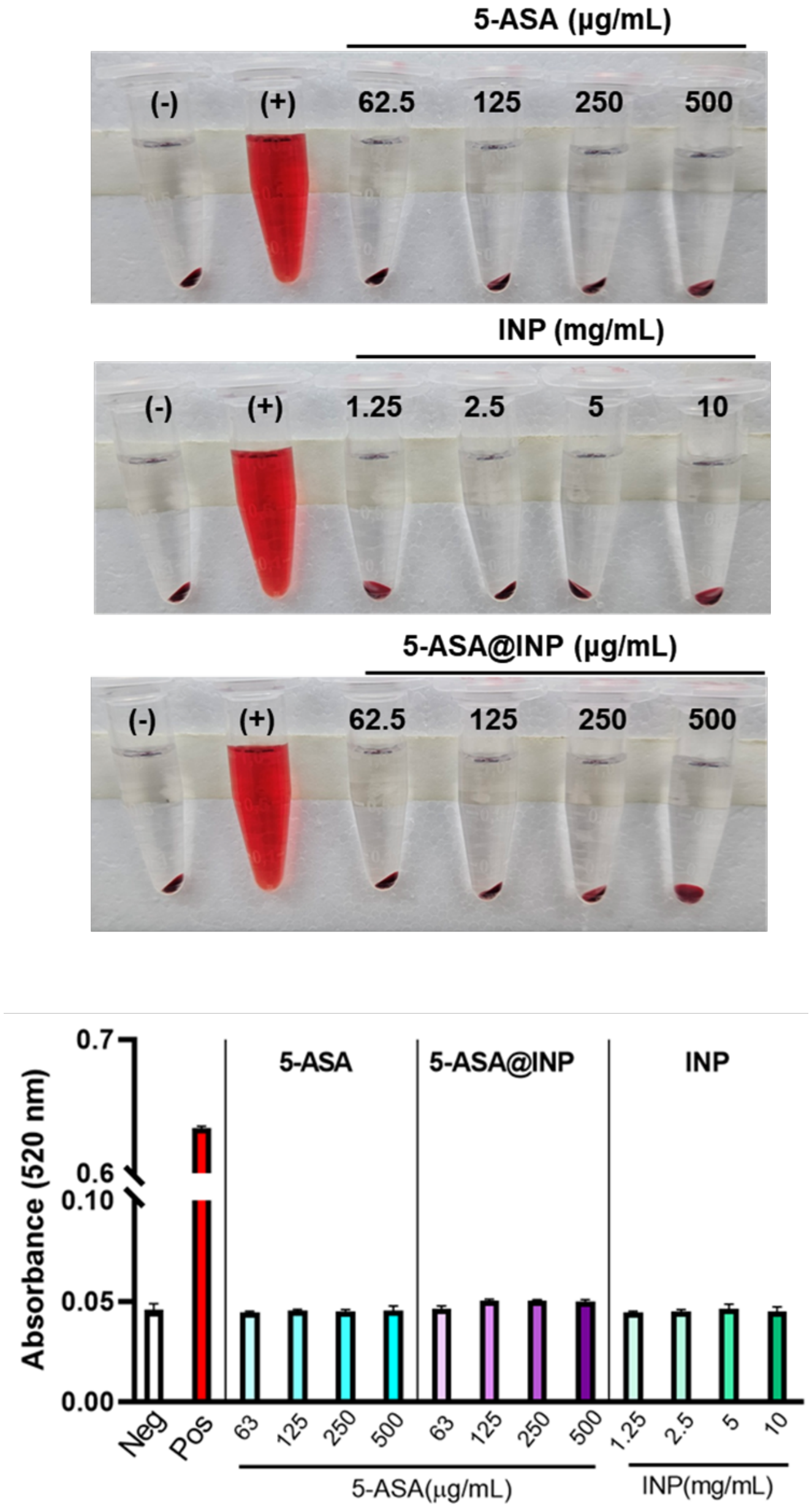

Figure S27. ***In vitro* hemolysis test of 5-ASA, INP, and 5-ASA@INP.** Red blood cells were incubated with 5-ASA or 5-ASA@INP at 62.5-500 μg/mL. The concentration of 5-ASA@INP was expressed as the equivalent concentration of loaded 5-ASA. INP was tested at 1.25-10 mg/mL based on nanoparticle mass. PBS and 0.1% Triton X-100 served as the negative (Neg; -) and positive (Pos; +) controls, respectively. Data are presented as mean ± standard deviation (SD, n = 3).

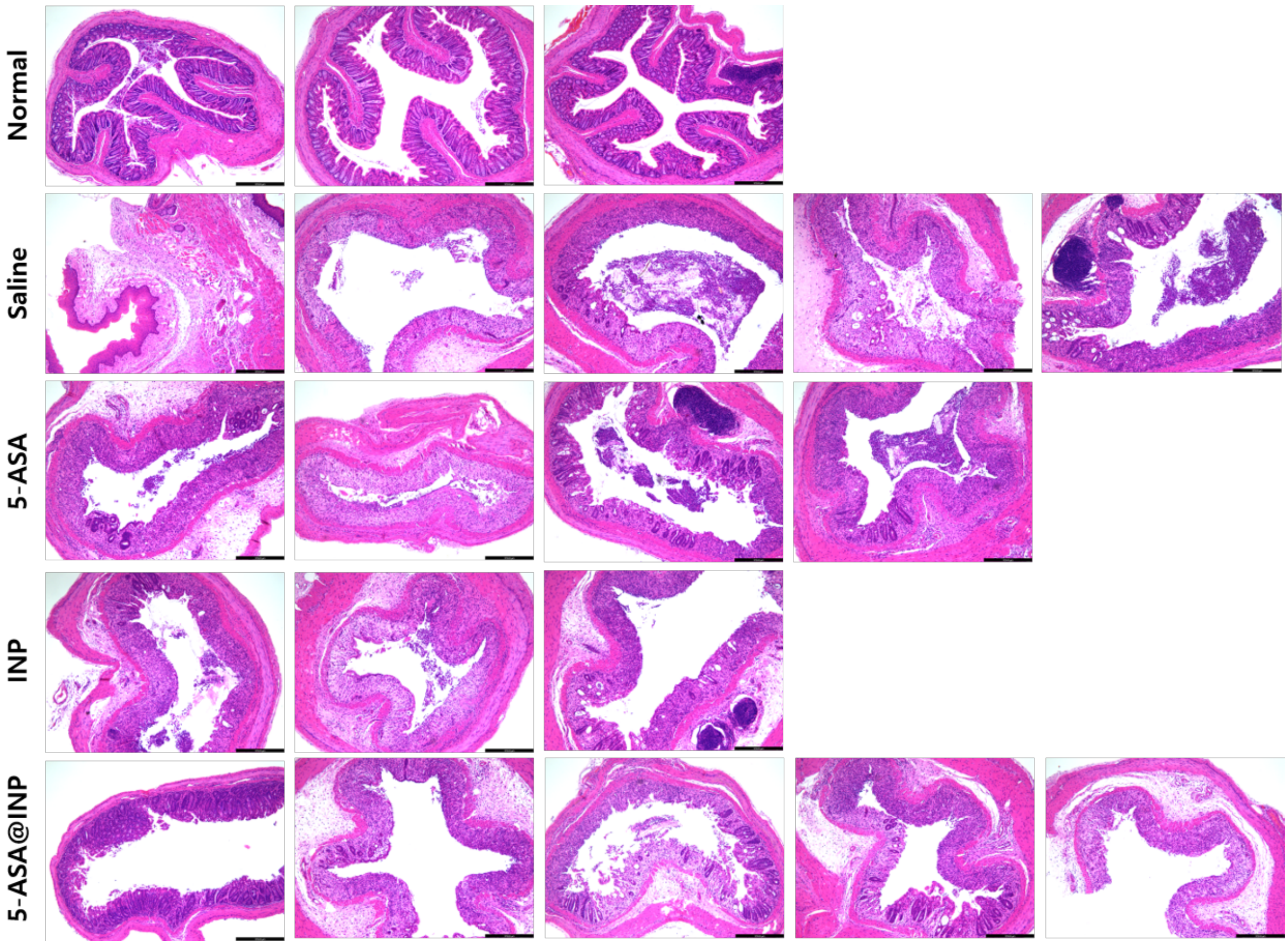

Figure S28. **Complete set of available H&E-stained colon sections from the original experimental cohort**. To ensure transparency and demonstrate the reproducibility of the therapeutic effects, this figure presents the complete set of histological sections from the initial animal study (prior to the confirmatory repeat experiment). Each row represents a different experimental group (Row 1: Normal (n = 3); Row 2: Saline (n = 5); Row 3: 5-ASA (n = 4); Row 4: INP (n = 3); Row 5: 5-ASA@INP (n = 5)). Scale bars = 316.9 μm; Magnification, ×100.
